# NSUN2 promoted tumor growth and metastatic via m^5^C-regulation of YAP through ALYREF/YBX1 axis in NSCLC

**DOI:** 10.1038/s41419-025-08353-x

**Published:** 2026-03-07

**Authors:** Rui Li, Dan Jin, Shuang Shao, Jiwei Guo

**Affiliations:** 1 Gastroenterology Department, BinZhou Medical University Hospital, BinZhou, China; 2 Medical Research Center, BinZhou Medical University Hospital, BinZhou, China

**Keywords:** Growth factor signalling, Cancer epigenetics

## Abstract

NSUN2-dependent RNA m^5^C methylation is essential for RNA stability, cellular metabolism, and intracellular transport. Aberrant *YAP* expression is closely associated with tumorigenesis and progression in human cancers. However, the molecular mechanism by which m⁵C regulates the growth and metastasis of NSCLC through modulating *YAP* expression remains incompletely understood. Our results demonstrate that YAP and NSUN2 play analogous roles in regulating NSCLC cell growth, migration, invasion, and EMT. NSUN2 increased m^5^C modification of *YAP* mRNA. ALYREF and YBX1 combined and then interacted with *YAP* mRNA in an m^5^C-dependent manner to increase *YAP* stability and translation. Importantly, NSUN2, ALYREF and YBX1 bind to each other and affected their interaction with *YAP* mRNA. Mechanistically, NSUN2 first initiates the m^5^C within *YAP* mRNA and then ALYREF recognizes m^5^C modification on *YAP* mRNA, YBX1 was more likely to bind to the transitive m^5^C from ALYREF and then promoted *YAP* mRNA stability through impeding the combination between AGO2 and *YAP* mRNA whereby increasing the expression of *YAP* with interaction with eIF3a and thus excessive cell growth and metastasis via regulation of YAP’s target genes of CTGF, Cyr61 MMP2, MMP9 in NSCLC. Moreover, NSUN2 is transcriptionally activated by the YAP-TEAD2 complex, forming a positive feedback loop that promotes tumor growth and metastasis, a process effectively suppressed by m^5^C inhibitors both in vivo and in vitro. Furthermore, our presented findings suggest that NSUN2 promotes tumor growth and metastasis by increasing ALYREF/YBX1-mediated *YAP* expression in NSCLC and effective inhibition of m^5^C modification might provide a potential treatment strategy for NSCLC.

## Introduction

Non-small cell lung cancer (NSCLC) accounts for approximately 80–85% of all lung cancer cases worldwide [1]. NSCLC usually progresses more slowly than small cell lung cancer (SCLC), but it can also rapidly spread to other parts of the body. Particularly, the five-year survival rate for NSCLC is about 24%, but it can vary widely depending on the stage. The prognosis of NSCLC depends on many factors, such as the grade, subtype, and response to treatment. The earlier NSCLC is detected and treated, the better the chances of survival. Despite multimodal therapy combining surgery and chemotherapy, patients with NSCLC exhibit poor survival outcomes due to metastatic dissemination and local invasion [2]. Consequently, identifying effective therapeutic targets to suppress tumor growth and invasive progression in NSCLC remains a critical imperative.

Currently, 170 RNA modifications have been successfully identified, of which methylation modification accounts for about two-thirds of the total RNA modification [3]. The m^5^C is the form of methylation within the fifth carbon atom of cytosine (C) [4, 5]. Functioning as a key epitranscriptomic mark in eukaryotic mRNAs, m⁵C deposition is enriched in GC-rich loci, particularly at prestart codon regions within CDS domains [6]. The m^5^C modification is dynamic and reversible, which’s formation and removal are regulated by methyltransferase and demethylase [7–9]. Recent research had shown that m^5^C modification exists in many types of RNAs and is catalyzed by a variety of “Writers” and “Eraser” [10]. The writer is highly conserved in prokaryotes and eukaryotes, which included the NSUN family protein (NSUN1, NSUN2, NSUN3, NSUN4, NSUN5, NSUN6 and NSUN7) and methyltransferase homolog 2 (DNMT2) [11]. NSUN2 is an S-adenosylmethionine (SAM)-dependent methyltransferase featuring an N-terminal RNA recognition motif (RRM) and a C-terminal Rossmann-fold catalytic domain that binds SAM cofactors [12, 13]. Moreover, the m^5^C modification mediated by NSUN2 can specifically regulate mRNA expression at different sites and then play an important role in the stability of mRNA [14–16]. Consequently, NSUN2 dysregulation drives many human pathologies, with compelling evidence implicating it as a pathogenic driver in tumorigenesis [17–21]. On the other hand, most RNA methylation modifications required the recognition proteins identified as “Readers” [10]. The m^5^C recognized proteins, such as ALYREF and YBX1, play a biological role in the tumor occurrence and development through binding to m^5^C sites initialized by NSUN2 [22, 23]. ALYREF drives nuclear export of mRNPs through preferential binding to mRNA termini [24], while functioning as a bZIP transcription factor coactivator that modulates hematopoietic differentiation programs, including erythropoiesis and leukemic transformation [25]. As another m^5^C “Readers”, YBX1, a core member of the Y-box protein family, functions as a dual nucleic acid-binding factor through its evolutionarily conserved cold shock domain (CSD) [26]. As a central regulator of nucleic acid metabolism, YBX1 plays an important role in transcription initiation, DNA damage response, mRNA lifecycle control, and oncogenic signaling pathways modulating proliferation, differentiation, autophagy, and stress-induced carcinogenesis [27, 28]. However, the molecular mechanisms by which ALYREF and YBX1 cooperatively regulate gene expression in an m⁵C-dependent manner, and their precise roles in tumorigenesis and progression was required to be further investigated in NSCLC. Thus, screening and further elucidating the biological effects of m^5^C inhibitors in NSCLC would have significant economic and societal impacts on therapeutic against lung cancer.

As a considerable growth-regulatory pathway, YAP signaling coordinates organ size control through an MST-LATS kinase cascade that inactivates oncogenic coactivators YAP/TAZ via phosphorylation-dependent cytoplasmic sequestration [29]. Deregulated nuclear YAP/TAZ-TEAD complexes drive transcriptional programs enabling uncontrolled proliferation [30, 31]. YAP target genes, such as *CTGF*, *CYR61*, *OCT4*, *TP73*, and *ZEB1*, along with their regulatory mechanisms, have been extensively studied and are known to control processes such as proliferation and metastasis [31]. While recent studies have placed growing focus on elucidating novel molecular components and alternative regulatory modalities within the YAP pathway, the intrinsic regulatory mechanisms governing *YAP*, the central effector protein of this pathway, remain incompletely understood. Therefore, the mechanisms underlying *YAP* activation, nuclear translocation, and downstream transcriptional regulation, particularly those involving m⁵C modification on *YAP* transcripts, need to be further explored.

Herein, we found that YAP expression is positively correlated with NSUN2 expression, and that these two proteins play similar roles in the regulation of NSCLC tumor growth and metastasis. Moreover, m^5^C-modified *YAP* mRNA is first recognized by ALYREF, and then YBX1 bind ALYREF to regulate *YAP* mRNA stability through impeding the interaction between AGO2 and *YAP* RNA whereby increasing the expression of *YAP* with interaction with eIF3a. Importantly, our data showed that *NSUN2* is a direct transcriptional target of the YAP-TEAD2 complex, establishing a positive feedback loop between NSUN2 and YAP-TEAD2 in NSCLC. Furthermore, the inhibitors of NSUN2 and YBX1 synergistically inhibits NSCLC tumor growth and metastasis by m^5^C mediated regulation of *YAP* in vitro and in vivo. Our study identifies inhibition of *YAP* RNA m⁵C modification as a promising therapeutic target for NSCLC, providing mechanistic insights into epitranscriptional regulation of oncogenesis.

## Materials And Methods

### Molecular biology

Myc-tagged YAP and Flag-tagged NSUN2, ALYREF, and YBX1 constructs were made using the pcDNA 3.1 vector (Invitrogen, Carlsbad, CA, USA). Sequences encoding the Myc epitope (EQKLISEEDL) and Flag epitope (DYKDDDDK) were added by PCR through replacement of the first Met-encoding codon in the respective cDNA clones.

### Cell lines and culture

Human lung normal cell line HBEC and NSCLC cell lines A549, H1299, H520, and H358 were purchased from American Type Culture Collections (Manassas, VA). Cell lines were cultivated in RPMI-1640 medium supplemented with 10% FBS (Hyclone, USA), penicillin/streptomycin (100 mg/mL). Culture flasks were kept at 37 ˚C in a humid incubator with 5% CO_2_.

### RNA m^5^C quantification using HPLC–tandem mass

Spectrometry mRNA was isolated from total RNA by using a Dynabeads mRNA Purification Kit (Thermo Fisher Scientific), and rRNA contaminants were removed by using a Ribo-Minus Eukaryote Kit (Thermo Fisher Scientific). Subsequently, mRNA was digested into nucleosides by using nuclease P1 and alkaline phosphatase and was then filtered with a 0.22 mm filter. The amount of m^5^C was measured according to HPLC–tandem mass spectrometry, following the published procedure [32]. Quantification was performed by using the standard curve obtained from pure nucleoside standards that were run with the same batch of samples. The ratio of m^5^C to C was calculated based on the calibrated concentrations.

### RNA immunoprecipitation assay

RNA immunoprecipitation (RIP) was performed using Magna RIP^TM^ RNA-Binding Protein Immunoprecipitation Kit (Millipore) according to the manufacturer′s instructions. Briefly, cells were collected and lysed in complete RIPA buffer containing a protease inhibitor cocktail and RNase inhibitor. Next, the cell lysates were incubated with RIP buffer containing magnetic bead conjugated with indicated antibody (Abcam) or control normal human IgG. The samples were digested with proteinase K to isolate the immunoprecipitated RNA. The purified RNA was finally subjected to qPCR to demonstrate the presence of the binding targets.

### MS2 coat protein system to enrich mRNA

The MS2 coat protein system was performed as described previously [33]. Briefly, stably expressed pcDNA3.1-*YAP*-*MS2*–12X (YAP-MS2) A549 cells were co-transfected with pcDNA3.1-MS2-GFP and relevant genes then cultivated in RPMI-1640 medium supplemented with 10% FBS (Hyclone, USA), penicillin/streptomycin (100 mg/mL). Culture flasks were kept at 37 °C for 48 h in a humid incubator with 5% CO_2_. The cell lysate from these transfected cells was immunoprecipitated by GFP antibody to enrich equal *YAP* mRNA then performed the following experiment.

### RNA pulldown

The RNA pulldown assay was performed following the protocol described in a previously published study. Briefly, harvested cells were rinsed and sonicated in NET-2 buffer (50 mM Tris-HCl, pH 7.4, 150–300 mM NaCl, 0.05% NP40, PMSF, Benzamidine). Cell lysates were incubated with biotin-labeled probes synthesized from Sangon (Shanghai, China) and then pulled down with streptavidin beads (Sigma-Aldrich). Precipitated RNA and proteins were subsequently subjected to RT-PCR and western blot analyses, respectively.

### Total m^5^C measurement

The total m^5^C content of Total RNA was determined using an m^5^C methylation quantification kit (ab185912). Briefly, after total RNA was isolated and purified, the bind RNA was planted to the assay wells and cultured with the capture antibody. After that, the wells were washed, and the detection antibody and enhancer solution were added. The m^5^C level was detected according to the absorbency.

### Analysis of publicly available datasets

To evaluate the correlation between the expression levels of NSUN2, ALYREF, YBX1, and YAP and the prognostic outcomes of patients, Kaplan-Meier survival curves were generated for NSCLC patients with low and high expression of these genes using the Kaplan-Meier Plotter tool (https://www.cancer.gov/tcga, www.kmplot.com/analysis and www.oncolnc.org) [34].

### In vivo experiments

To assess the in vivo effects of NSUN2 and YAP, 3- to 5-week-old female BALB/c athymic nude mice were housed in a level 2 biosafety laboratory and raised according to the institutional animal guidelines of BinZhou Medical University. All animal experiments were carried out with the prior approval of the BinZhou Medical University Committee on Animal Care. For the experiments, mice were randomly assigned to groups and subsequently injected with 5 × 10^6^ A549 cells with stable expression of relevant plasmids and randomly divided into indicated groups (five mice per group). To assess the in vivo effects of NSUN2 and YAP, the xenografted tumors had reached approximately 5 mm in diameter from mice. Tumor volume was estimated as 0.5 × a^2^ × b (where a and b represent a tumors short and long diameter, respectively). Mice were euthanized after six weeks, and the tumors were measured at the final time. Experiments were terminated when subcutaneous tumors in mice reached a diameter of 20 mm or a volume of 2000 mm³, in accordance with animal ethics guidelines for minimizing potential suffering. Tumor and organ tissue were then collected from xenograft mice and analyzed by immunohistochemistry.

### Statistical analysis

Each experiment was repeated at least three times. The statistical analyses of the experiment data were performed by using a two-tailed Student’s paired T-test and one-way ANOVA. Statistical significance was assessed at least three independent experiments, and significance was considered. Prior to conducting parametric tests, we assessed data normality via the Shapiro–Wilk test and subsequently employed the Pearson correlation coefficient to analyze the correlation between variables. *P*-value < 0.05 was considered statistically significant and highlighted an asterisk in the figures, while *P*-values < 0.01 were highlighted using two asterisks and *P*-values < 0.001 highlighted using three asterisks in the figures.

## Results

### NSUN2 and YAP collectively drive tumorigenesis and malignant progression in NSCLC

**Fig. 1:**
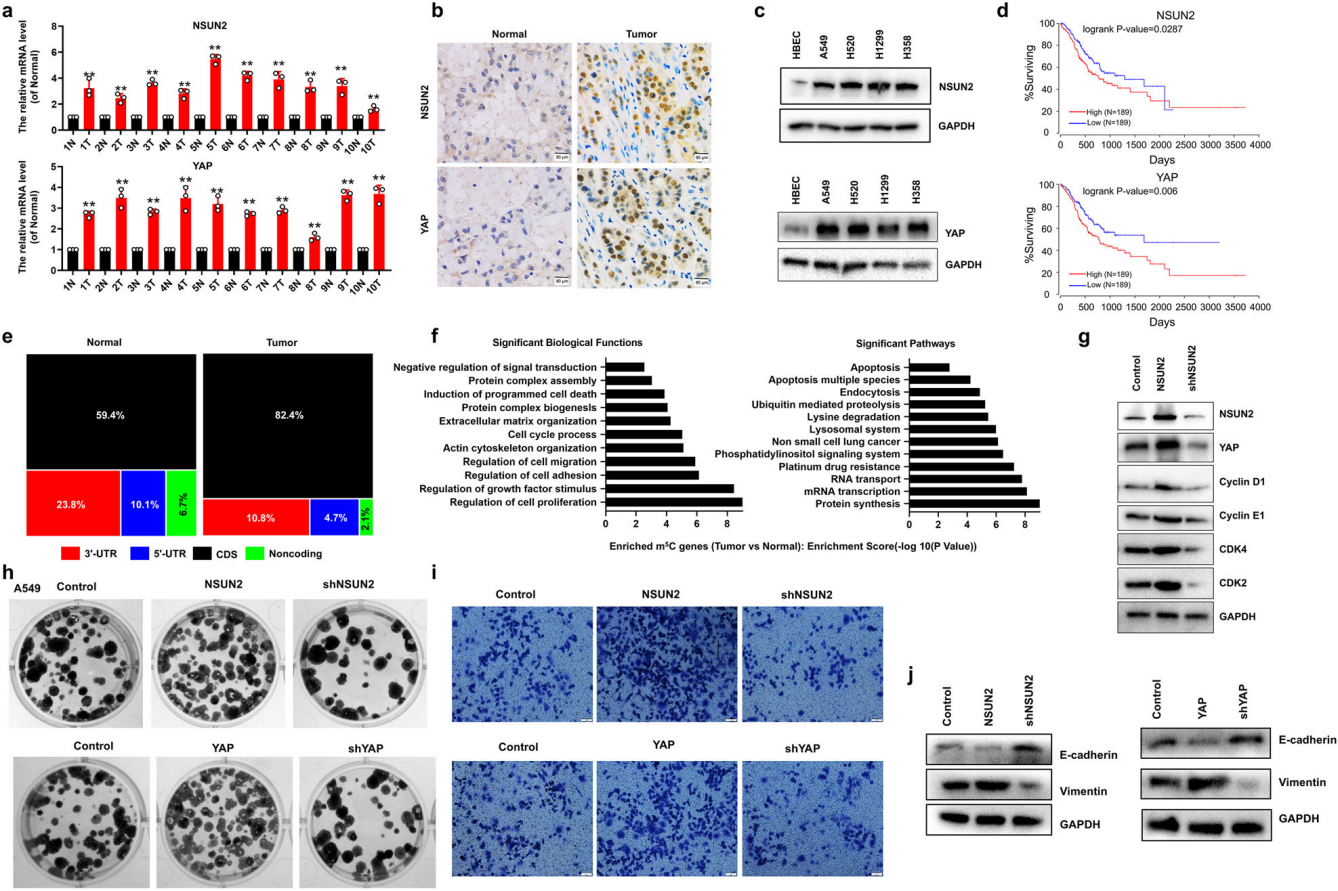
NSUN2 and YAP play a similar role in driving the occurrence and progression in NSCLC. **a**, **b** mRNA levels (*n* = 10**, a**) and immunohistochemical staining (*n* = 5**, b**) of NSUN2 and YAP in NSCLC samples. **c** Protein levels of NSUN2 and YAP in NSCLC cell lines. **d** High expression of NSUN2 or YAP was associated with the worse prognosis for NSCLC patients (*P* = 0.0287 for NSUN2; *P* = 0.006 for YAP). **e** Square chart depicting the fraction of m^5^C peaks in 4 transcript segments. **f** Gene ontology (GO) enrichment analysis of tumor methylated genes in NSCLC patients. **g–j** Protein levels of NSUN2, YAP, Cyclin D1, Cyclin E1, CDK4 and CDK2 (**g**), number of colon (**h**), cellular invasion growth (**i**) and EMT (**j**) in A549 cells. Results were presented as mean ± SD of three independent experiments. *P < 0.05 or **P < 0.01 indicates a significant difference between the indicated groups.

Fifty-six surgically resected NSCLC specimens were obtained from patients undergoing pulmonary resection at the Affiliated Hospital of BinZhou Medical University (BinZhou, Shandong, China) between January 2020 and January 2022. These specimens were utilized to evaluate the clinical significance of NSUN2 and YAP. Clinicopathological correlations of NSUN2 and YAP protein expression with tumor histopathological grades are summarized in Table 1. We found that NSUN2 and YAP expressions were strongly associated with tumor migration, size, and differentiation but not with age, gender, or smoking history (Table 1). Based on the analysis of the TCGA database, the expression levels of NSUN2 and YAP demonstrate a significant positive correlation with the progression grading of both lung adenocarcinoma (LUAD) and lung squamous cell carcinoma (LUSC). This finding suggests that these molecular markers may play a crucial role in the tumor progression of these two major histological subtypes of lung cancer (Figure S1a). In addition, the NSUN2 and YAP levels were higher in the lung cancer tissues than in the normal adjacent tissues (*n* = 10, Fig. 1a and *n* = 5, Fig. 1b). Moreover, the NSUN2 and YAP levels were increased in NSCLC cells referred to as A549, H520, H1299 and H358 compared to the normal lung cell HBEC (Fig. 1c and Figure S1b). Furthermore, analysis of publicly available TCGA-LUAD datasets (https://www.cancer.gov/tcga) revealed that higher expression levels of NSUN2 and YAP were significantly correlated with shorter overall survival (OS) (*P* = 0.0287 for NSUN2; *P* = 0.006 for YAP) (Fig. 1d). Importantly, RNA m^5^C quantification using HPLC–tandem mass indicted that the m^5^C level was significantly increased in the tumor tissues compared to the normal adjacent tissues (*n* = 10) (Figure S1c). m^5^C-seq technology was used in our study to clarify the distribution pattern of m^5^C peaks within the genome. We found that m^5^C peaks in tumor tissues were more enriched wherein CDS region compared to normal tissues (Fig. 1e). Dysregulated m⁵C-related genes in tumors exhibited Gene Ontology (GO) enrichment for proliferation, growth factor signaling, and adhesion pathways, paralleling the oncogenic characteristics associated with YAP overexpression-induced phenotypes. (Fig. 1f) [35]. Our data showed that cellular growth was increased in A549 cells with transfection of NSUN2 or YAP but decreased in A549 cells with transfection of shNSUN2 (using the shNSUN2-2) or shYAP (using the shYAP-2) compared to controls, respectively (Figure S1d–f). Additionally, the expressions of CyclinD1/CDK4 and CyclinE1/CDK2, the cell cycle regulatory proteins related to cellular promoted proliferation, were increased in the A549 cells with transfection of NSUN2 compared to control (Fig. 1g). Moreover, the corresponding contrary results of Cleaved-Caspse3 (Figure S1g), Annexin V expressions (Figure S1h) and corresponding similar results of the clone formation growth (Fig. 1h and Figure S1i), migration (Figure S1j), invasion (Fig. 1i) and EMT (Fig. 1j and Figure S1k) were obtained in A549 and H1299 cells undergoing these treatments, respectively. These results indicated that the strong positive correlation between YAP and NSUN2 levels reflects their important action in accelerating tumor growth and metastasis in NSCLC.

**Table 1:** Patient’s demographics and tumor characteristics and association of NSUN2 and YAP levels with clinicopathological features.

Characteristics	No. of patients, *N* = 56 (%)	*P*-value
Patients Parameter		
Age (years)		0.971
Average [range]	55 [35–81]	
<53	19 (33.9)	
≥53	37 (66.1)	
Gender		0.614
Male	39 (69.6)	
Female	17 (30.4)	
Smoking history		
Smoker	37 (66.1)	0.120
Non-smoker	19 (33.9)	
Tumor Characteristics		
Tumor size (cm)		0.005**
<4	9 (16.1)	
≥4	47 (83.9)	
Differentiation		0.011*
Poor	41 (73.2)	
Well-moderate	15 (26.8)	
Lymph node metastasis		0.013*
N-	8 (14.2)	
N+	48 (85.0)	
Distant metastasis		0.004**
M-	11 (19.6)	
M+	45 (80.4)	
Expression of NSUN2		
Protein level		
high	50 (89.2)	0.004**
median	5 (8.9)	0.198
low	1 (1.9)	0.989
mRNA level		
high	49 (87.5)	0.001**
median	4 (7.1)	0.693
low	3 (5.4)	0.865
Expression of YAP		
Protein level		
high	51 (91.1)	0.002**
median	4 (7.1)	0.787
low	1 (1.8)	0.904
mRNA level		
high	48 (80.4)	0.001**
median	3 (5.4)	0.103
low	4 (7.2)	0.689

Differences between experimental groups were assessed by Student’s t-test or one-way analysis of variance. Data represent mean ± SD. *p < 0.05; **p < 0.01.

### NSUN2 promotes cellular proliferation, invasion and EMT through m⁵C-dependent regulation of YAP signaling in NSCLC

**Fig. 2:**
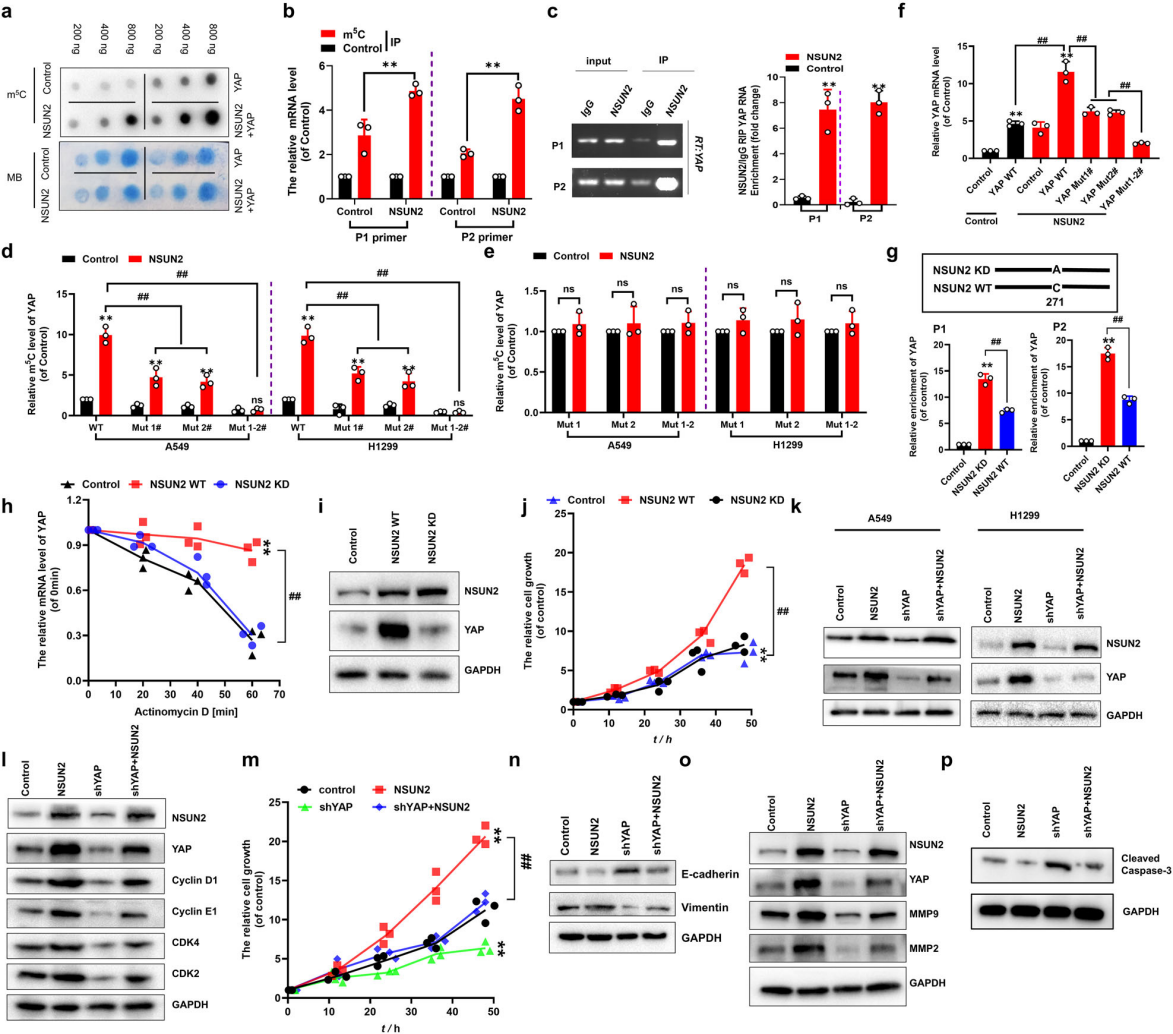
NSUN2 effected NSCLC growth and metastasis via regulation of *YAP* in a m^5^C-dependent manner. **a** Dot blot assay of m^5^C methylation in transfected A549 cells. **b** m^5^C level of *YAP* mRNA detected by m^5^C-RIP-qPCR assay. **c** RIP assay of interaction between NSUN2 and *YAP* mRNA using the Primer 1 (P1) detected the S1 site and Primer 2 (P2) detected the S2 site. **d**, **e** m^5^C level determined by m^5^C-RIP-qPCR assay. **f** mRNA levels of *YAP* in the A549 cells. **g** RIP assay of the interaction between NSUN2 WT/KD and *YAP* mRNA. **h–j** Stability of *YAP* mRNA (**h**), protein levels of NSUN2 and YAP (**i**) and cellular growth (**j**) in A549 cells. **k–p** protein levels of NSUN2, YAP (**k**, **l**), CyclinD1, CyclinE1, CDK4 and CDK2 (**l**), cellular growth (**m**), EMT (**n**, **o**) and apoptosis (**p**) in A549 cells with co-transfection of NSUN2 and sh*YAP*. Results were presented as mean ± SD of three independent experiments. *P < 0.05, **P < 0.01 or ^*#*^*P* < 0.05 or ^*##*^*P* < 0.01 indicates a significant difference between the indicated groups.

Our result showed that NSUN2 increased the methylation in A549 cells and more importantly, NSUN2 could directly increase the methylation for *YAP* RNA (Fig. 2a and Figure S2a). Genome-wide mapping revealed m⁵C deposition in evolutionarily conserved coding regions [36], with CCCGGG emerging as the top conserved motif—validating previously reported methylation signatures [37]. Bioinformatic analysis identified two evolutionarily conserved motifs (herein referred to as S1: 403-ATGGAT**CCCGGG**CAGCAG-420 and S2: 1336-TCTTCT**CCCGGG**ATGTCT-1353) within *YAP* RNA transcripts, potentially mediating regulatory functions and we engineered synonymous mutations at predicted m⁵C sites within the *YAP* transcript (Figure S2b). Unambiguously, NSUN2-mediated m⁵C deposition was significantly enriched at two functional sites within *YAP* RNA determined by the m^5^C-RIP-qPCR assay (Fig. 2b). To rule out potential interference from m^5^C site mutations on NSUN2-*YAP* binding efficiency, we assessed their interaction and found that the binding affinity remains consistent between NSUN2 and *YAP*-WT (Fig. 2c and Figure S2c, d) as well as *YAP* Muts# (Figure S2e). Notably, *YAP* Muts# contains a single mutant m^5^C site while maintaining the normal function of the remaining m^5^C sites within *YAP* RNA transcripts. While NSUN2 elevated m⁵C levels in all *YAP* variants, but *YAP* Muts# diminished enhancement efficacy compared to *YAP*-WT, confirming motif-dependent modification (Fig. 2d). However, NSUN2 overexpression failed to elevate m⁵C modification levels in the YAP-Mut1-2# transcript, which carries synonymous mutations at all predicted m⁵C sites (Fig. 2d). Consistent with these observations, NSUN2 knockdown in A549 and H1299 cells significantly reduced m⁵C modification levels compared to scrambled shRNA controls (Figure S2f). Complete m⁵C site mutagenesis (*YAP* Muts) rendered *YAP* transcripts refractory to modification changes upon NSUN2 overexpression compared to controls, confirming site-directed ablation of regulatory capacity (Fig. 2e and Figure S2g). Our data also showed that m⁵C modification directly modulated *YAP* transcript level, as NSUN2 overexpression enhanced *YAP*-WT but not *YAP*-Muts# levels (Fig. 2f and Figure S2h, i). The NSUN2 KD (C271A), which functionally declines the release of target genes from NSUN2-compound substance [38], was used to explore whether influence on *YAP* expression in NSCLC cells (Fig. 2g). Our result indicated that NSUN2 KD indeed decreased the release of *YAP* mRNA from the NSUN2 KD/YAP component determined by the RIP (Fig. 2g and Figure S2j) and RNA pulldown (Figure S2k) assays. The stability of *YAP* mRNA (Fig. 2h) and the YAP protein (Fig. 2i) levels were increased in the A549 cells with transfection of NSUN2 WT compared to the control but repressed with transfection of NSUN2 KD. Similar as the YAP expression, the results of cell growth (Fig. 2j), viability (Figure S2l) and clone formation (Figure S2m) were obtained in the A549 cells under these treatments, respectively. Importantly, NSUN2 enhanced *YAP* mRNA stability (Figure S2n) and protein expression (Fig. 2k) in the A549 and H1299 cells. sh*YAP* co-transfection reversed NSUN2-driven YAP elevation (Fig. 2k), establishing epistatic regulation of oncogenic phenotypes. NSUN2-driven YAP activation concomitantly modulated cell cycle regulators (Fig. 2l) then promoted proliferation (Fig. 2m) and invasion (Figure S2o). But phenotypic rescue by sh*YAP* confirmed pathway dependency. NSUN2 promoted EMT (Fig. 2n, o and Figure S2p) but suppressed apoptosis (Fig. 2p) through YAP expression. These data showed that NSUN2 promotes cellular proliferation, invasion and EMT through m⁵C-dependent regulation of YAP signaling in NSCLC.

### m^5^C readers ALYREF and YBX1 regulated NSCLC cell growth, migration, invasion and EMT through YAP

**Fig. 3:**
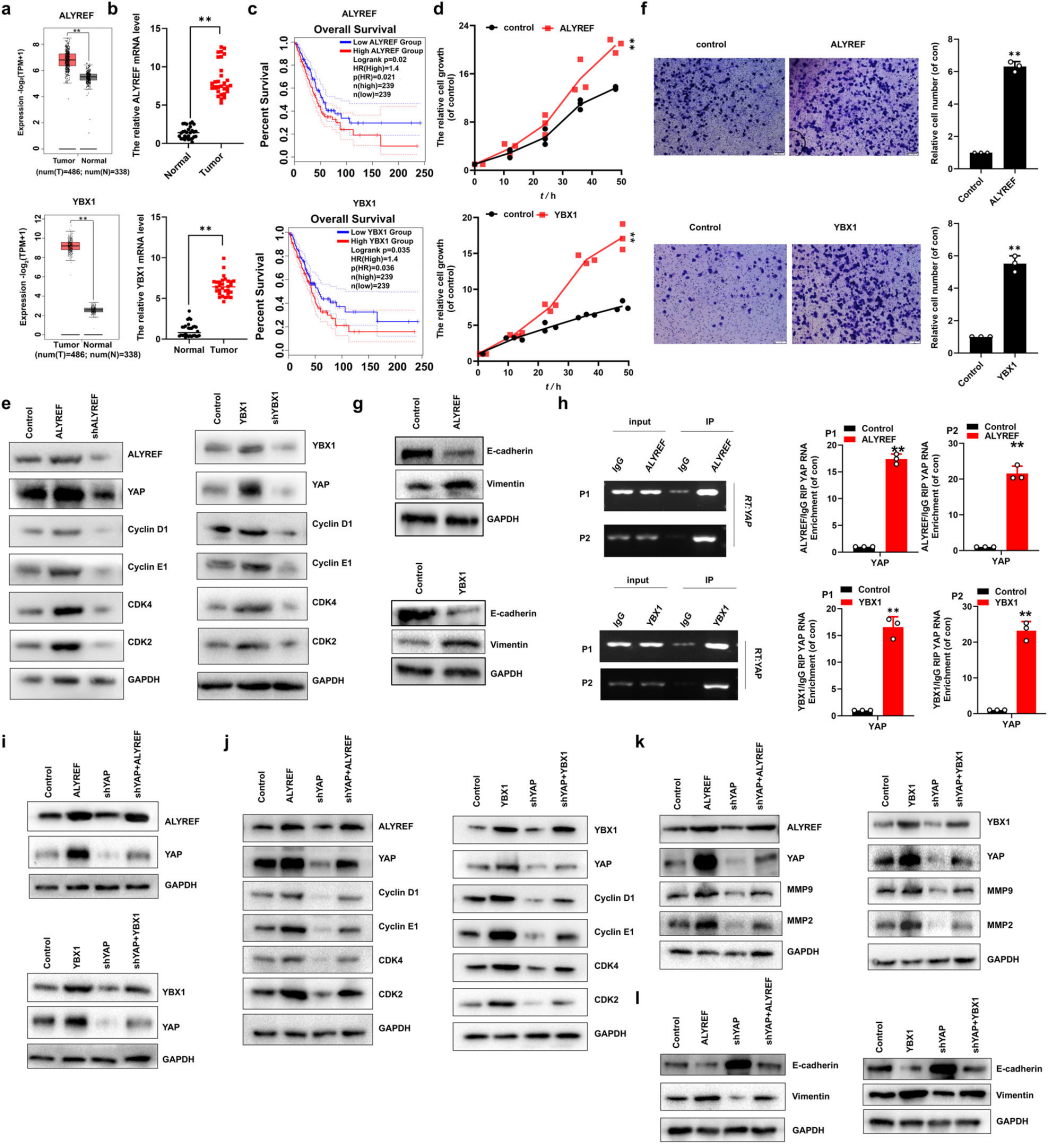
m^5^C readers ALYREF/YBX1 regulated NSCLC cell growth, migration, invasion and EMT through YAP. **a** The TCGA database of ALYREF and YBX1 levels (N = 338, T = 486). **b** qPCR assay of *ALYREF* and *YBX1* mRNA levels in NSCLC samples (*n* = 30). **c** High expression of ALYREF or YBX1 was associated with worse prognosis for NSCLC patients (*P* = 0.021 for ALYREF; *P* = 0.036 for YBX1). **d–g** cellular growth (**d**), immunoblotting of ALYREF, YBX1, YAP, CyclinD1, CyclinE1, CDK4 and CDK2 (**e**), cellular invasion (**f**), EMT (**g**) in the A549 cells. **h** RIP assay of interactions between ALYREF or YBX1 and *YAP* mRNA. **i–k** immunoblotting of ALYREF, YBX1, YAP (**i**), CyclinD1, CycliE1, CDK4 and CDK2 (**j**), MMP9, MMP2 (**k**) and EMT (**l**) in the A549 cells with co-transfection of ALYREF/YBX1 and sh*YAP*. Results were presented as mean ± SD of three independent experiments. *P < 0.05 or **P < 0.01 indicates a significant difference between the indicated groups.

TCGA analysis showed elevated ALYREF/YBX1 expression in NSCLC tumors (*n* = 486) relative to patient-matched normal tissues (*n* = 338) (Fig. 3a). Our RT-PCR data showed similar results in the NSCLC samples (*n* = 30, Fig. 3b). Moreover, ALYREF/YBX1 expressions were increased in the NSCLC cells than in their normal control cell, HBEC (Figure S3a). Analysis of publicly available TCGA-LUAD datasets revealed that the ALYRFE/YBX1 expressions were negative correlation with the patient overall survival (*P* = 0.021 for ALYREF; *P* = 0.036 for YBX1) (Fig. 3c). Moreover, over-expressed ALYREF/YBX1 (Figure S3b) increased but shALYREF/shYBX1 (Figure S3b) decreased cellular growth (Fig. 3d) and viability (Figure S3c) in the A549 cells. Concordantly, similar results of the expressions of the CyclinD1/CDK4 and CyclinE1/CDK2 (Fig. 3e), cellular migration (Figure S3d), clone formation (Figure S3e), invasion (Fig. 3f) and EMT (Fig. 3g and Figure S3f) were obtained in NSCLC cells undergoing these treatments, respectively. Furthermore, the TCGA data showed that ALYREF expression was positively related to YBX1 (Figure S3g). Our data also showed that ALYREF/YBX1 directly binds to the *YAP* mRNA analyzed by RIP (Fig. 3h) and RNA pulldown (Figure S3h) assays. The stability of *YAP* mRNA was increased or decreased in Actinomycin D-treated A549 cells with transfection of YBX1 or shYBX1, respectively (Figure S3i). The protein and mRNA level of YAP was increased in the A549 cells with transfection of ALYREF/YBX1 but back down these levels with co-transfection of shYAP in the A549 cells (Fig. 3i and Figure S3j). Consistent with YAP expression, the cell viability and growth (Figure S3k, l), the expressions of CyclinD1/CDK4 and CyclinE1/CDK2 (Fig. 3j), the migration growth (Figure S3m, n), the expressions of MMP2, MMP9 (Fig. 3k) and EMT (Fig. 3l and Fig. S3o) were obtained the corresponding similar results in the A549 cells undergoing these treatments. These results showed that ALYREF and YBX1 promoted cell growth, invasion and EMT via regulation of YAP.

### NSUN2 controls tumor growth and metastasis through m⁵C-dependent regulation of the ALYREF/YBX1 molecular axis in NSCLC

**Fig. 4:**
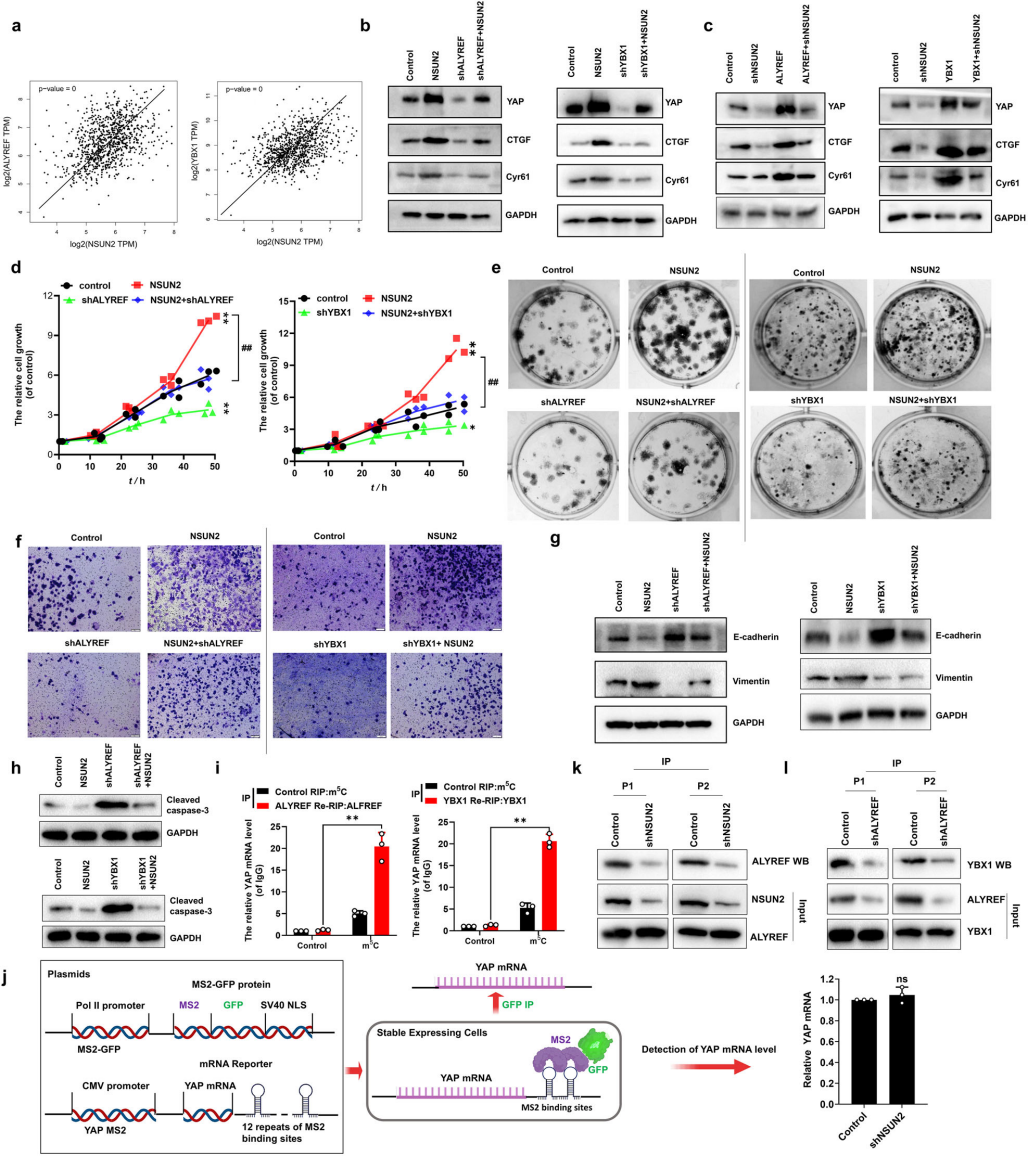
NSUN2 regulated cell growth and metastasis through m^5^C-mediated ALYREF/YBX1 axis in NSCLC. **a** The positive correlation between ALYREF/YBX1 and NSUN2 from TCGA database. **b–h** immunoblotting of YAP, CTGF and Cyr61 (**b**, **c**), cellular growth (**d**), colon formation growth (**e**), cellular invasion growth (**f**), EMT (**g**) and apoptosis (**h**) in A549 cells with transfected the relevant genes. **i** RIP assay of m^5^C-mediated interactions between the ALYREF/YBX1 and *YAP* mRNA. **j** The diagram of MS2 coat protein system in A549 cells to enrich the equivalent *YAP* mRNA (elements from BioRender.com.). **k**, **l** RNA pulldown assay of interaction between ALYREF (**k**) or YBX1 (**l**) and *YAP* mRNA using the MS2 coat protein system. Results were presented as mean ± SD of three independent experiments. *P < 0.05, **P < 0.01 or ^#^*P* < 0.05, ^##^*P* < 0.01 indicates a significant difference between the indicated groups. ns, not significant.

The TCGA database revealed a positive correlation between NSUN2 and ALYREF/YBX1 levels in NSCLC tissues (Fig. 4a). Moreover, the expressions of YAP and its target genes *CTGF* and *Cyr61* were increased in the A549 cells with transfection of *NSUN2* but recovered by co-transfection of sh*ALYREF* or sh*YBX1*, respectively (Fig. 4b). Clearly, the expressions of YAP and its target genes *CTGF* and *Cyr61* were decreased in the A549 cells with transfection of sh*NSUN2* but raised by co-transfection of *ALYREF* or *YBX1* (Fig. 4c). Consistent with YAP expression, the corresponding similar results of the cellular growth (Fig. 4d and Figure S4a), clone formation (Fig. 4e and Figure S4b), invasion (Fig. 4f and Figure S4c), MMP2, MMP9 (Fig. S4d) and EMT (Fig. 4g and Figure S4e) but contrasting results regarding the expression of Cleaved Caspase 3 (Fig. 4h) were obtained in the A549 cells undergoing these treatments, respectively. Moreover, RIP assays showed that ALYREF/YBX1 specifically recognizes m⁵C-modified *YAP* mRNA (Fig. 4i). Next, the constructs containing *YAP* mRNA transcripts combined with the MS2 binding site were generated and co-transfected into A549 cells with a construct containing MS2-binding protein and GFP (Fig. 4j). An anti-GFP RNA immunoprecipitation (RIP) assay was performed to enrich equivalent endogenous *YAP* mRNA (Figure S4f). RNA pulldown combined with western blot analysis demonstrated that ALYREF specifically binds to m^5^C-modified *YAP* mRNA, an epigenetic modification catalyzed by NSUN2 (Fig. 4k). Interestingly, using the MS2 coat protein system, we further identified that YBX1 selectively interacts with m^5^C-modified *YAP* mRNA following its transfer from ALYREF in A549 cells (Fig. 4l). Collectively, these results demonstrate that NSUN2 catalyzes m^5^C modification of *YAP* mRNA, which facilitates YBX1 binding to the m^5^C-modified *YAP* transcript following its transfer from ALYREF, ultimately promoting tumor growth and metastasis in NSCLC.

### NSUN2, ALYREF and YBX1 synergistically regulate cellular growth and metastasis via regulation of *YAP* mediated by AGO2 and eIF3a in NSCLC cells

**Fig. 5:**
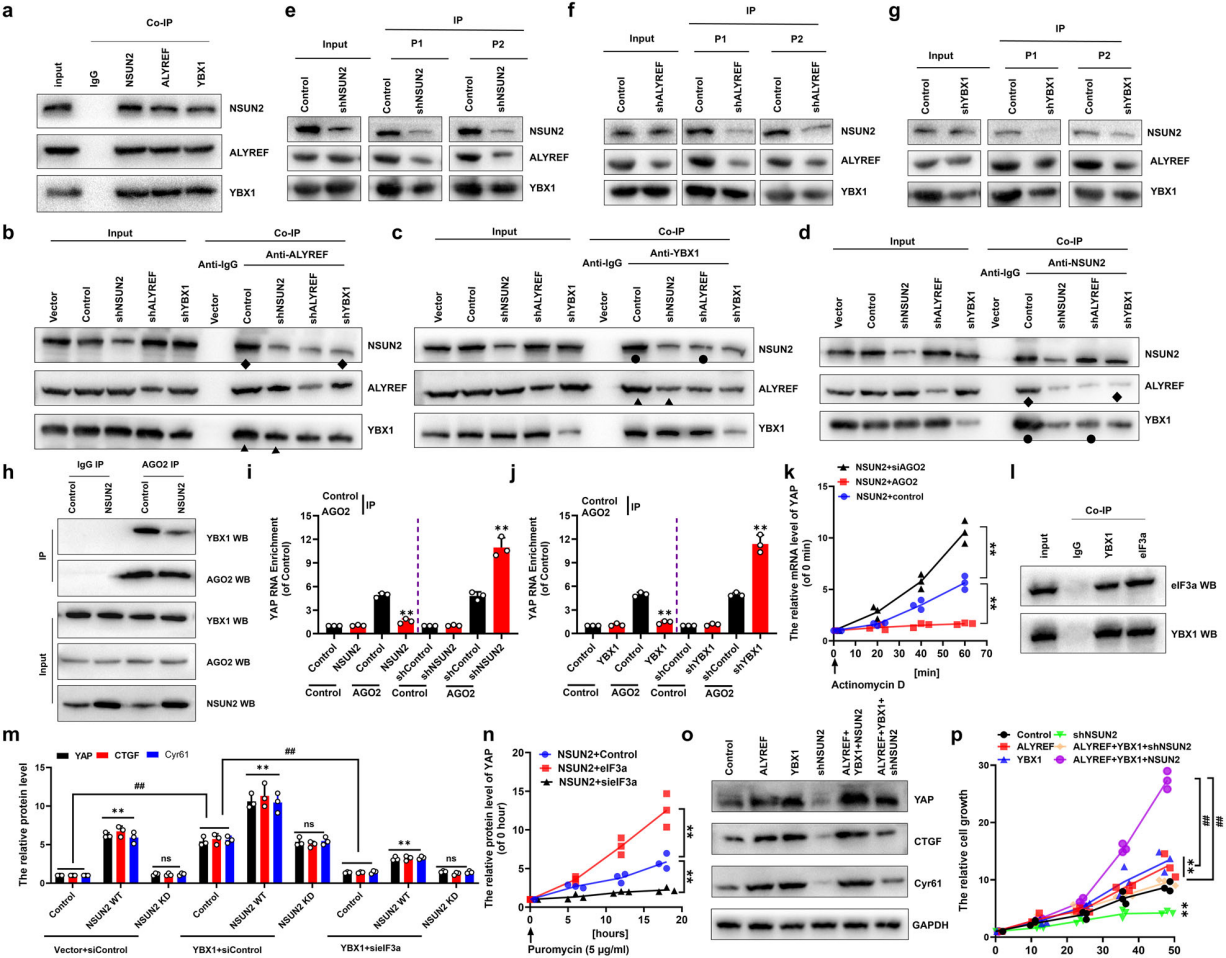
NSUN2, ALYREF and YBX1 synergistically regulate cellular growth and metastasis via regulation of YAP mediated by AGO2 and eIF3a in NSCLC cells. **a–d** Co-IP assay assessing endogenous interactions among NSUN2, ALYREF, and YBX1. **e–g** RNA pulldown assay assessing interactions between *YAP* mRNA and NSUN2, ALYREF, YBX1 used anti-GFP. **h** Co-IP assay assessing endogenous interactions between YBX1 and AGO2. **i**, **j** RIP assay of relation between AGO2 and *YAP* RNA. **k** qPCR assessing the stability of *YAP* mRNA. **l** Co-IP assay assessing endogenous interaction between YBX1 and eIF3a. **m–p** protein levels of YAP, CTGF and Cyr61 (**m–o**) and cellular growth (**p**) in the A549 cells with transfected the relevant genes. Results were presented as mean ± SD of three independent experiments. **P < 0.01 or ^##^*P* < 0.01 indicates a significant difference between the indicated groups.

The Co-IP data showed that NSUN2, ALYREF and YBX1 bind each other, respectively (Fig. 5a). Importantly, Co-IP assay showed that knockdown of NSUN2 reduced the combining capacity between ALYREF and YBX1, as denoted by triangular symbols (Fig. 5b, c). Concordantly, knockdown of ALYREF diminished the interaction between NSUN2 and YBX1, as evidenced by circular symbols (Fig. 5c, d). Similarly, knockdown of YBX1 reduced the interaction between NSUN2 and ALYREF, as indicated by diamond symbols (Fig. 5b, d), in A549 cells. Moreover, NSUN2 knockdown significantly attenuated ALYREF/YBX1 binding affinity for *YAP* mRNA in MS2-tagged RNA capture systems (Fig. 5e and Figure S5a). Consistently, ALYREF knockdown attenuated NSUN2 and YBX1 recruitment to *YAP* mRNA (Fig. 5f and Figure S5b) and, similarly, YBX1 depletion impaired NSUN2 and ALYREF binding to *YAP* mRNA (Fig. 5g and Figure S5c), demonstrating NSUN2-ALYREF-YBX1 codependent complex assembly on the *YAP* mRNA. Next, mechanistic studies revealed YBX1 cooperates with NSUN2 to enhance mRNA levels of *YAP*, *CTGF* and *Cyr61*, whereas AGO2, a known mRNA destabilizer [39], antagonized this stabilization (Figure S5d). Importantly, endogenous Co-IP assays demonstrated that elevated NSUN2 expression attenuates direct YBX1-AGO2 interactions (Fig. 5h). RIP assays demonstrated an inverse relationship between NSUN2/YBX1 expression and AGO2-*YAP* mRNA interactions: elevated NSUN2 (Fig. 5i) or YBX1 (Fig. 5j) expression significantly reduced AGO2 binding to *YAP* mRNA, whereas their depletion enhanced AGO2 recruitment to *YAP* mRNA. qPCR assay showed that AGO2 reduced the stability of *YAP* mRNA in the co-transfected A549 cells with NSUN2 (Fig. 5k) or YBX1 (Figure S5e) compared to control. Intriguingly, the endogenous co-IP assays revealed that YBX1 interacts with eIF3a, a core subunit of eukaryotic translation initiation factor 3 [40] (Fig. 5l). Consistent with this interaction, protein levels of YAP, CTGF, and Cyr61 were significantly reduced in A549 cells following co-transfection with YBX1 and sieIF3a (Fig. 5m). The quantitative ELISA assay showed that eIF3a improved the YAP protein level in the co-transfected A549 cells with NSUN2 (Fig. 5n) or YBX1 (Figure S5f) compared to control. Moreover, our data showed that co-transfection of ALYREF, YBX1, and NSUN2 synergistically enhanced YAP, CTGF, and Cyr61 expression (Fig. 5o). Conversely, co-transfection with ALYREF, YBX1, and shNSUN2 markedly suppressed these proteins (Fig. 5o and Figure S5g). Furthermore, consistent with YAP expression, the corresponding similar results of the cell growth (Fig. 5p), the cellular viability (Figure S5h–j), the clone formation (Figure S5k, l), the migration growth (Figure S5m) and the invasion growth (Figure S5n, o) were obtained in the A549 cells undergoing these treatments, respectively. These findings demonstrate that NSUN2, ALYREF, and YBX1 form a tripartite complex that synergistically binds *YAP* mRNA and enhances its stability by impeding AGO2-*YAP* RNA interaction then this stabilization facilitates eIF3a-mediated translation of YAP protein, ultimately driving excessive cell growth and metastasis in NSCLC through upregulation of downstream effectors CTGF and Cyr61.

### *NSUN2* is a direct target gene of YAP-TEAD2 to form a positive-feedback loop

**Fig. 6:**
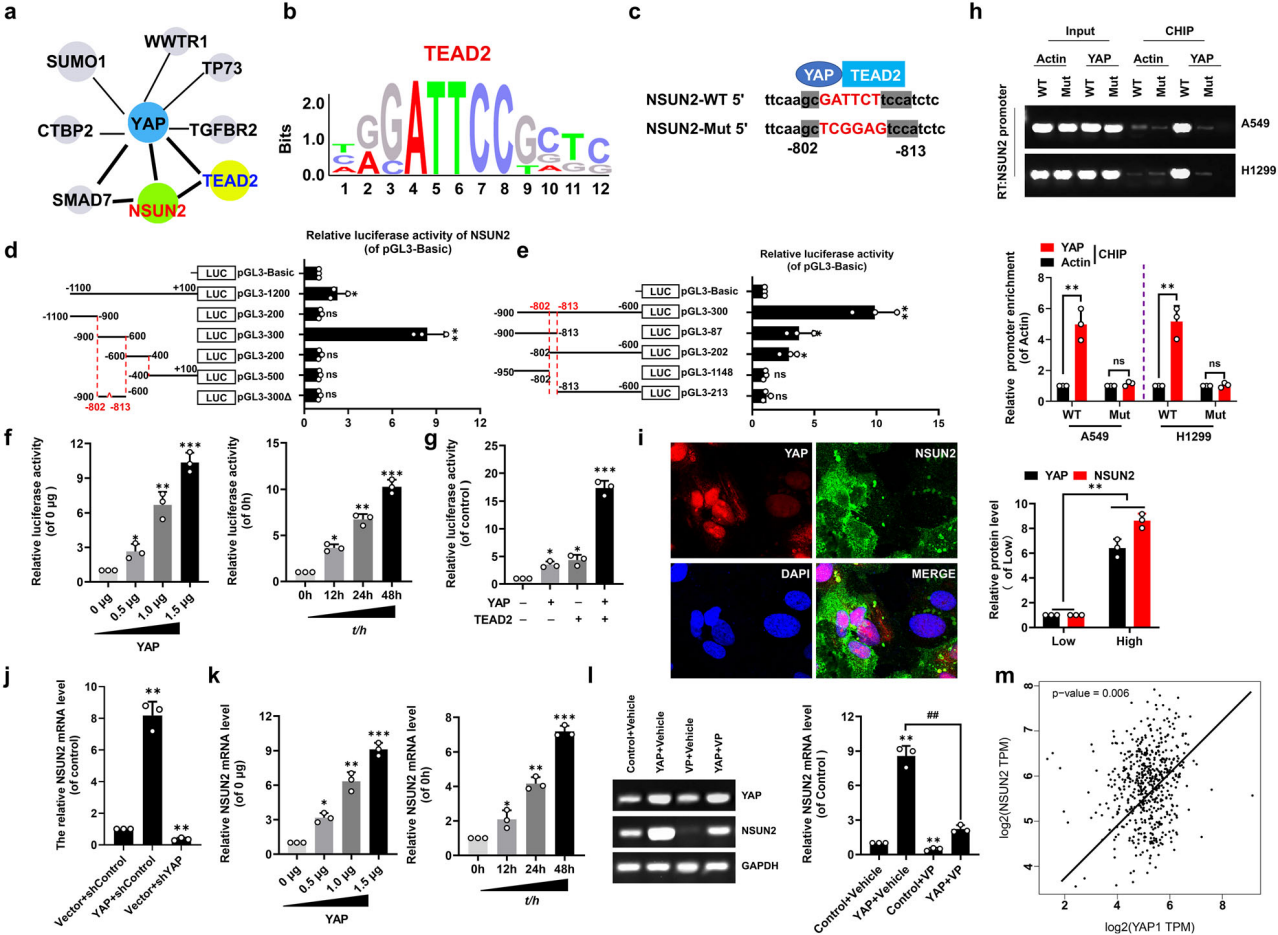
NSUN2 and YAP-TEAD2 form a positive-feedback loop. **a** Bioinformatics predicted the relation between YAP and NSUN2 (https://genemania.org/). **b**, **c** The JASPAR database showed the conservative binding motif of TEAD2 (**b**) and the binding sites within the *NSUN2* promotor (**c**). **d−g** LLuciferase reporter gene assays of *NSUN2* promoter in A549 cells. **h** ChIP assay analyzed the relation between YAP and *NSUN2* WT/Mut promoter in the A549 and H1299 cells. **i** immunofluorescence of YAP and NSUN2 in YAP-transfected A549 cells. **j−l** qPCR of *NSUN2* (**j**, **k**), RT-PCR of *YAP* and *NSUN2* (**l**) in A549 cells. **m** The TCGA database showed the positive relation between YAP and NSUN2. Results were presented as mean ± SD of three independent experiments. *P < 0.05, **P < 0.01, ***P < 0.001 or ^##^*P* < 0.01 indicates a significant difference between the indicated groups. *ns*, not significant.

Firstly, bioinformatics analysis (GeneMANIA, https://genemania.org/) suggested that YAP interacts with NSUN2 (Fig. 6a). Additionally, JASPAR analysis showed that TEAD2 (YAP transcription factor) preferentially binds to a universal consensus motif (Fig. 6b) inside the *NSUN2* promoter (−802 ~ −813) (Fig. 6c). Secondly, various lengths of the *NSUN2* 5’-flanking regions were cloned to determine the *NSUN2* promoter activity in the transfected A549 cells with over-expressing YAP (Fig. 6d, e). The consensus motif was in pGL3-300, where luciferase activity was highest, suggesting that the predicted region (−802 ~ −813) is the core *NSUN2* promoter site for interaction with YAP/TEAD2 (Fig. 6d, e). Moreover, YAP activated the pGL3-300 reporter in a dose- and time-dependent manner in A549 cells (Fig. 6f). Acting as a TEAD2 coactivator, YAP transcriptionally regulated *NSUN2* promoter activity (Fig. 6g). ChIP analysis demonstrated direct YAP-TEAD2 binding to the *NSUN2* WT promoter, with complete abrogation using the mutated *NSUN2* Mut in both A549 and H1299 cell lines (Fig. 6h). Accordingly, NSUN2 expression increased following YAP transfection but decreased upon shYAP transfection determined by immunofluorescence (Fig. 6i) and qPCR (Fig. 6j) assay as well as it was found to exhibit a dose- and time-dependent manner (Fig. 6k). Notably, verteporfin (VP), which disrupts YAP-TEAD2 complex formation, abolished YAP-driven *NSUN2* regulation (Fig. 6l). Thirdly, TCGA database indicated that the YAP protein has a positive correlation with NSUN2 protein in NSCLC (Fig. 6m). These data demonstrate that *NSUN2* is a direct transcriptional target of the YAP-TEAD2 complex, establishing a positive feedback loop between NSUN2 and YAP-TEAD2 in NSCLC.

### NSUN2 promoted tumor growth and metastasis by increasing the expression of YAP in vivo

**Fig. 7:**
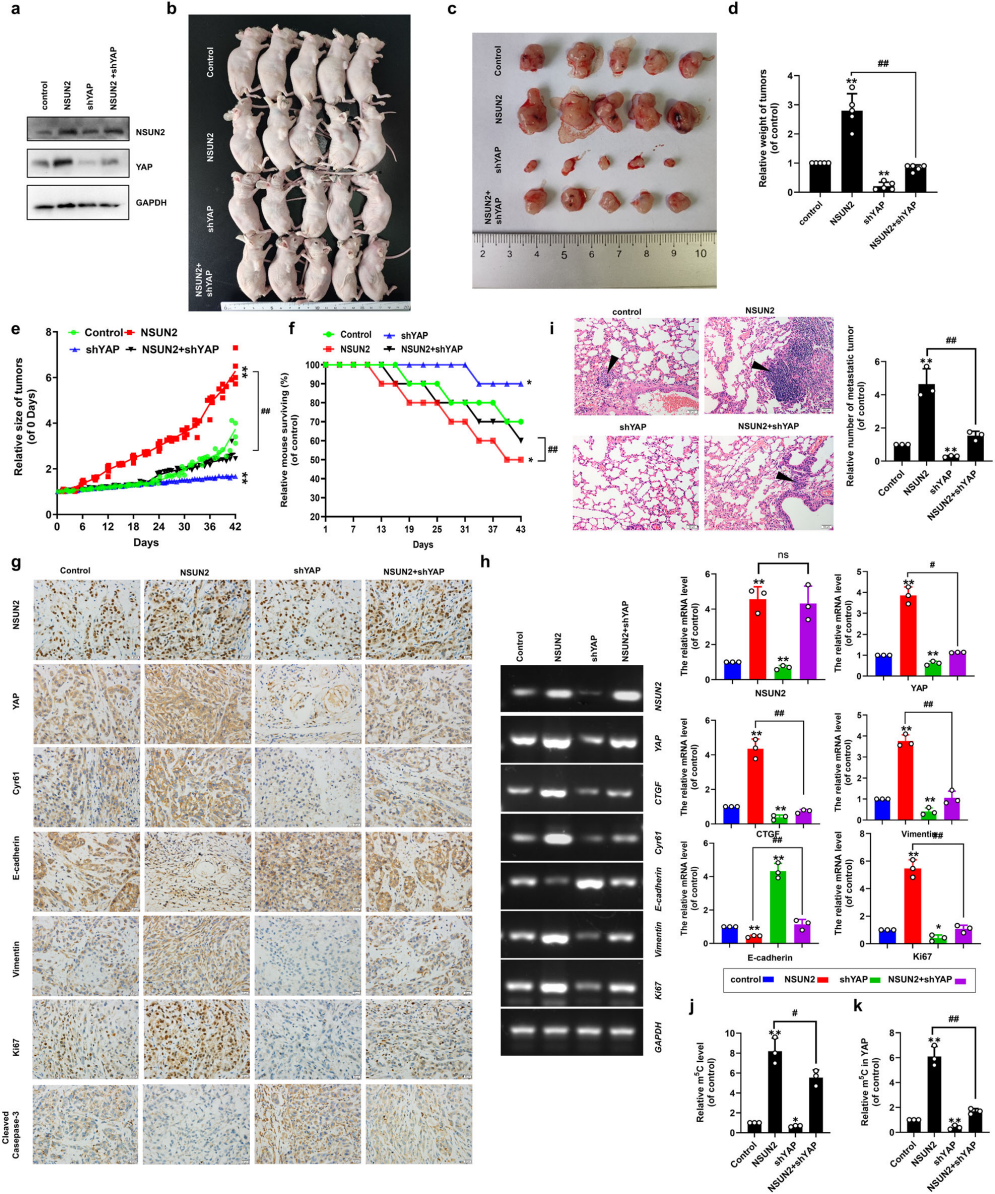
NSUN2 promoted tumor growth and metastasis via increasing the expression of YAP in vivo. **a** Immunoblotting of NSUN2 and YAP in the A549 stable-genes expression cells. **b**, **c** The tumors in xenografted mice. **d–f** The tumor weight (**d**), size (**e**) and overall survival (**f**) in the xenografted mice. **g**, **h** The protein and mRNA levels of indicated genes in xenografted mice (*n* = 5). **i** Representative H&E-stained microscopic images of metastatic lung tumors in xenografted mice. **j**, **k** The relative m^5^C level of total (**j**) and *YAP* mRNA (**k**) in the xenografted lung tumor. Results were presented as mean ± SD of three independent experiments. **P < 0.01 or ^#^*P* < 0.05, ^##^*P* < 0.01 indicates a significant difference between the indicated groups.

Given the functional synergy between NSUN2 and YAP in driving NSCLC pathogenesis, we established A549 cell lines with stable co-overexpression of *NSUN2* and *YAP*. Successful stable protein expression was confirmed by western blot before utilizing these cells to generate mouse xenograft tumor models (Fig. 7a). Six weeks post-subcutaneous implantation, NSUN2-overexpressing xenograft mice exhibited significantly larger tumors (Fig. 7b–d), accelerated tumor growth (Fig. 7e), and reduced survival compared to controls (Fig. 7f). Conversely, shYAP xenograft mice showed attenuated tumor burden, slower progression, and extended survival (Fig. 7b–f). NSUN2+shYAP groups partially rescued the oncogenic phenotype, yielding smaller tumors and improved survival relative to NSUN2-overexpressing xenograft mice (Fig. 7b–f). Moreover, quantitative IHC (*n* = 5, Fig. 7g and Figure S6a–g), RT-qPCR, and qPCR (Fig. 7h) analyses of xenograft tumor revealed elevated YAP, Cyr61, Vimentin, and Ki67 expression but reduced E-cadherin and cleaved caspase-3 levels in NSUN2-overexpressing tumors compared to controls. The contrary result with their expressions were obtained in the shYAP group compared to the control group (Fig. 7g, h and Figure S6a–g). Further, NSUN2+shYAP reversed these expressions compared to the NSUN2 group (Fig. 7g, h and Figure S6a–g). Furthermore, NSUN2-overexpressing xenograft mice developed significantly more numerous and larger lung metastatic lesions compared to controls, while shYAP xenograft mice exhibited attenuated metastatic activity (Fig. 7i). NSUN2+shYAP groups substantially reduced metastasis relative to NSUN2-overexpressing xenograft mice (Fig. 7i). Notably, global m⁵C levels (Fig. 7j) and *YAP* mRNA m^5^C level (Fig. 7k) were elevated in NSUN2-group lung metastatic tumor compared to controls. These observations indicated that m^5^C initiated by NSUN2 controls tumor growth and metastasis in a YAP-dependent manner in vivo.

### The inhibitors of NSUN2 and YBX1 synergistically impede NSCLC tumor growth and metastasis by regulation of *YAP*

**Fig. 8:**
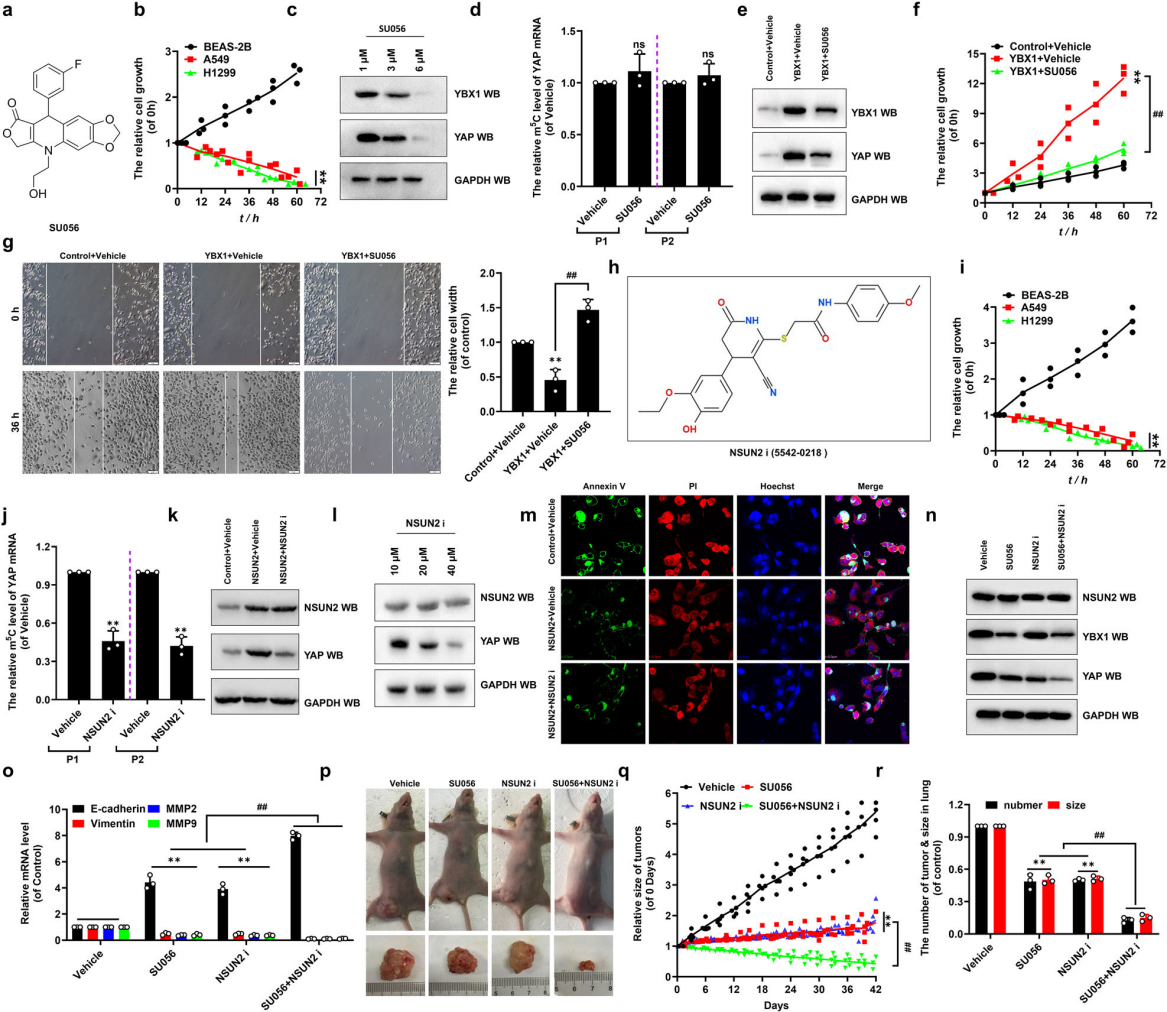
The inhibitors of NSUN2 and YBX1 synergistically impede NSCLC tumor growth and metastasis by regulation of YAP. **a** The chemical formula of YBX1 inhibitor SU056. **b** The cellular growth in the BEAS-2B, A549 and H1299 cells with treatment of SU056 at 3.25 μM, respectively. **c–g** Protein levels of YBX1 and YAP (**c**), m^5^C level (**d**), immunoblotting of YBX1 and YAP (**e**), cellular growth (**f**) and cellular migration growth (**g**) in the SU056-treated A549 cells. **h** The chemical formula of NSUN2 inhibitor NSUN2 i. **i** The cellular growth in the BEAS-2B, A549 and H1299 cells with treatment of NSUN2 i at 20.5 μM, respectively. **j**–**m** m^5^C level of *YAP* mRNA (**j**), protein levels of NSUN2 and YAP (**k, l**) and apoptosis (**m**) in the NSUN2 i-treated A549 cells. **n**, **o** protein levels of NSUN2, YBX1, YAP (**n**) and mRNA levels of *E-cadherin*, *Vimentin*, *MMP2* and *MMP9* (**o**) in A549 cells with co-treatment of SU056 and NSUN2. **p**, **q** The tumor size (**p**) and growth (**q**) in the xenografted mice. **r** The number of tumors in the xenografted lung. Results were presented as mean ± SD of three independent experiments. **P < 0.01 or ^##^*P* < 0.01 indicates a significant difference between the indicated groups.

Collectively, our findings establish YBX1 as the critical mediator linking the NSUN2-ALYREF-YBX1 complex to *YAP* mRNA regulation (Fig. 5). To functionally validate this axis, we assessed how YBX1 depletion impacts NSCLC pathogenesis in vitro and in vivo. The small-molecule compound SU056 (CAS No.:2376580-08-2), screened from ChemDiv, was shown to influence YBX1 (Fig. 8a). Critically, SU056 demonstrated selective anti-tumor activity, showing no cytotoxicity in normal bronchial epithelial cells while significantly suppressing proliferation in the A549 and H1299 cells (Fig. 8b). SU056 dose- and time-dependently suppressed A549 cell viability, with IC₅₀ values of 7.19 μM (36 h), 3.25 μM (48 h), and 2.94 μM (60 h) respectively (Figure S7a). Based on the established 48 h IC₅₀ (3.25 μM), all subsequent assays used SU056-treated A549 cells under these conditions. SU056 induced dose- and time-dependent downregulation of both YBX1 and YAP (Fig. 8c and Figure S7b). Crucially, m⁵C modification patterns on *YAP* mRNA showed no drug-induced alterations (Fig. 8d), demonstrating SU056’s selective action on protein expression without perturbing epigenetic regulation. Moreover, YAP expression was increased in *YBX1*-transfected A549 cells compared to controls but was restored upon co-treatment with SU056 (Fig. 8e). Consistent with YAP expression, the similar results for the expressions of *CTGF* and *Cyr61* (Figure S7c), cellular growth and viability (Fig. 8f and Figure S7d), migration (Fig. 8g) and invasion (Figure S7e) growth and EMT (Figure S7f) were obtained in the same treated A549 cells, respectively. These results demonstrate that SU056 inhibits proliferation, migration, and EMT by downregulating YBX1-mediated YAP expression independent of m⁵C modification in NSCLC cells. Interestingly, our data also showed that the small-molecule compound NSUN2 i (ChemDiv:5542-0218) affected the functions of NSUN2 (Fig. 8h). CCK-8 assays revealed that NSUN2 i exhibited no cytotoxicity in normal cells (BEAS-2B) but significantly suppressed proliferation in A549 and H1299 cells (Fig. 8i) with IC₅₀ values of 45.68 μM (36 h), 20.52 μM (48 h), and 15.47 μM (60 h), respectively (Figure S7g). Subsequent experiments used 20.52 μM NSUN2 i (48 h IC₅₀) for A549 treatments. Crucially, NSUN2 i treatment significantly reduced *YAP* mRNA m⁵C levels compared to vehicle controls (Fig. 8j) without altering NSUN2 protein expression (Fig. 8k), demonstrating selective inhibition of catalytic activity rather than protein stability of NSUN2 in NSCLC cells. Additionally, NSUN2i dose-dependently reduced YAP levels (Fig. 8l). Moreover, NSUN2 transfection elevated YAP expression in A549 cells compared to vector controls, while NSUN2i co-treatment reversed this effect (Fig. 8k). Concordantly, similar results of the mRNA levels of *CTGF* and *Cyr61* (Figure S7h), cellular viability (Figure S7i), the migration and invasion growth (Figure S7j) and the EMT-related genes of Vimentin, MMP2 and MMP9 (Figure S7k) but the contrary results of the EMT-related marker E-cadherin (Figure S7k) and apoptosis-related marker Annexin V (Fig. 8m and Figure S7l) were obtained in the A549 cells with the same treatments. Our findings demonstrate that the NSUN2 inhibitor suppresses proliferation, migration, and EMT in NSCLC cells through m⁵C-dependent regulation of the NSUN2-YAP axis. Given that both SU056 and NSUN2i suppress proliferation, migration, invasion, and EMT in A549 cells via regulation of YAP, we investigated their synergistic potential against NSCLC progression. NSUN2i solely reduced m⁵C levels compared to vehicle controls, whereas SU056 co-treatment did not further potentiate this effect (Figure S7m). Importantly, combinatorial treatment dramatically decreased YAP and its target genes compared to either individual treatment (Fig. 8n and Figure S7n). Consistent with YAP expression, the similar results for the cellular viability (Figure S7o), clone formation growth (Figure S7p) and EMT (Fig. 8o) were obtained in the same treated A549 cells, respectively. Notably, combinatorial SU056 and NSUN2i treatment attenuated tumor progression (Fig. 8p, q), reduced final tumor weight (Figure S7q) but extended overall survival (Figure S7r) in A549 cell xenograft mice compared to monotherapy. qPCR analysis revealed that combinatorial SU056 and NSUN2i treatment markedly reduced levels of oncogenic effectors (YAP, Ki67, Cyr61, CTGF) and EMT markers (Vimentin, MMP2/9) compared to single agent treatment in xenograft tumor (Figure S7s). Conversely, EMT related markers E-cadherin were elevated in combinatorial treatment groups (Figure S7s). Statistically, dual therapy significantly attenuated lung metastatic activity compared to single agent treatments (Fig. 8r). These data showed that the inhibitors of NSUN2 and YBX1 synergistically impede NSCLC tumor growth and metastasis by regulation of YAP.

## Discussion

The incidence and mortality of lung cancer have always been at the forefront of all malignant tumors in humans. The GLOBOCAN data showed that there were 2,206,771 new cases and 1,796,144 death cases of global lung cancer in 2020 [1]. The lung cancer incidence is modestly lower than the breast cancer, however its mortality rate remains the leading cause of cancer-related deaths globally. Although clinically targeted and immunotherapy can prolong the survival of patients, the 5-year survival rate for the invasive lung adenocarcinoma in stage I is only 55% [1]. Due to the limited awareness of physical examinations in China, most lung cancer cases are diagnosed at an advanced stage, posing a significant threat to public health and imposing substantial medical pressure. This data showed that m^5^C inhibitors synergistically inhibited NSCLC growth and metastatic via downregulation of *YAP* through NSUN2-ALYREF-YBX1 axis. Our study elucidates the molecular mechanism by which NSUN2-mediated m⁵C modification, facilitated by the ALYREF-YBX1 complex, regulates *YAP* to drive NSCLC tumorigenesis and metastasis. Our findings establish m⁵C-modified *YAP* as a promising therapeutic target for NSCLC.

Recent studies establish RNA modifications-notably m^6^A, m^5^C and m^1^A-as emerging mechanisms of post-transcriptional gene regulation [4]. Among >160 known RNA chemical modifications, m^6^A and m^5^C represent the most prevalent and functionally characterized types [5]. m^5^C methylation is dynamically regulated by writers (methyltransferases), erasers (demethylases), and readers (RNA-binding proteins) [9]. The m⁵C modification dynamically regulates RNA biology, including mRNA subcellular localization, transcript stability, translational efficiency, and structural maintenance [9]. Emerging evidence implicates m⁵C dysregulation in tumor initiation, progression, and metastatic dissemination in many human cancers. For example, elevated expression of m⁵C regulators NSUN3 and NSUN4 in lung tumors correlates with adverse clinicopathological features and reduced survival [41]. In this study, NSUN2 overexpression promoted cellular proliferation, invasion, migration, and EMT in NSCLC cells (Fig. 1). Notably, NSUN2 mediated m⁵C deposition on *YAP* mRNA, driving its m⁵C-dependent upregulation in NSCLC (Fig. 2).

**Fig. 9:**
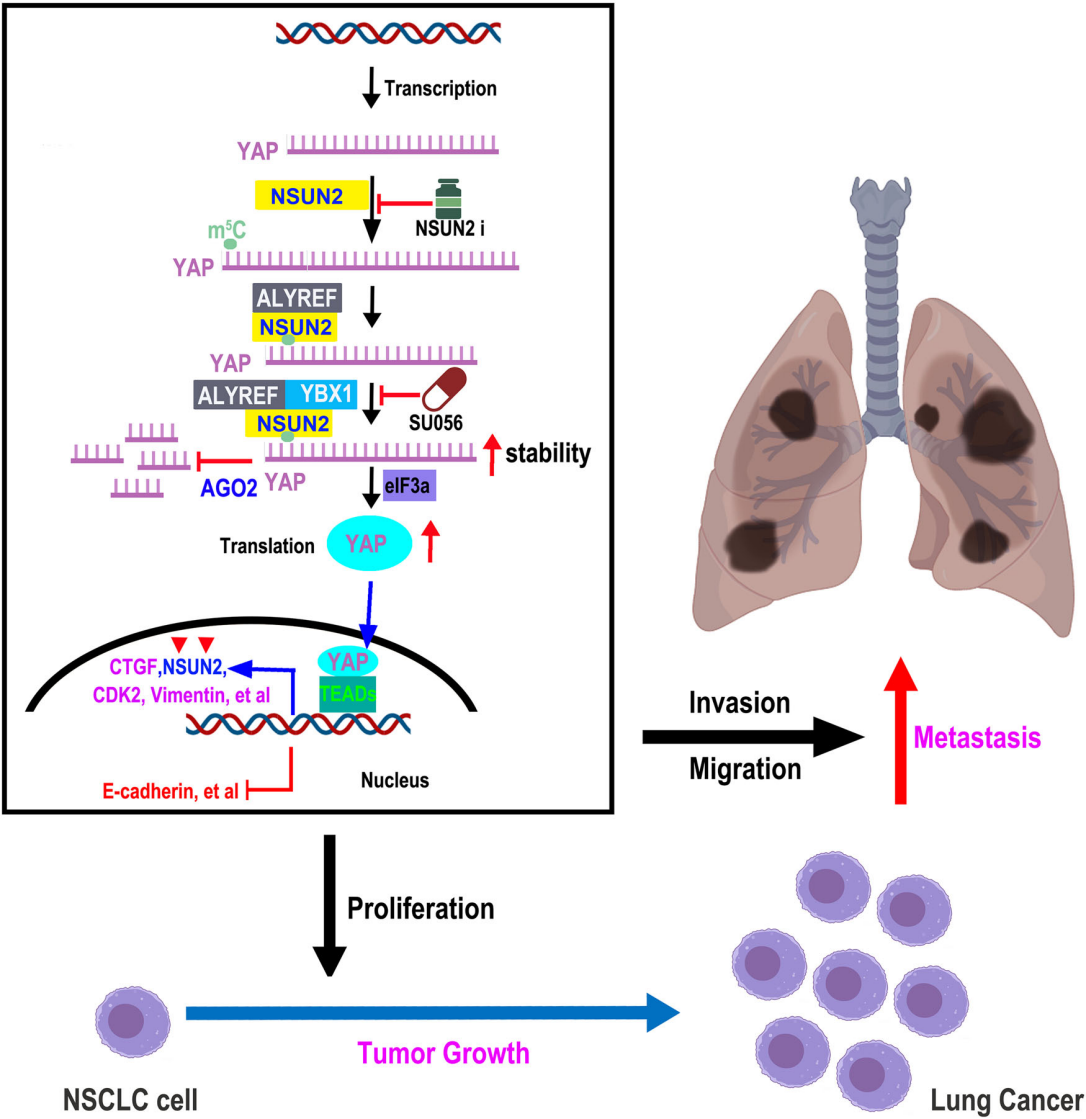
The diagram of NSUN2 promotes NSCLC growth and metastasis by regulating YAP expression through ALYREF/YBX1 axis. The figure is created using elements from BioRender.com.

The m^5^C binding proteins (“Readers”) are primarily consists of two components ALYREF and YBX1. ALYREF, functioning as an RNA-binding protein, plays important roles in 5’RNA capping, RNA polymerase II elongation, transcriptional splicing, and mRNA export [27]. Importantly, ALYREF has been found to be closely involved in the m^5^C modification, which is a mRNA nucleoplasm export factor. The another “Readers” is YBX1 which regulates mRNA stabilization. This study demonstrates that m⁵C-modified *YAP* mRNA is sequentially recognized by ALYREF (Fig. 4k) and subsequently bound by YBX1 to determine its transcript fate (Fig. 4l). We further uncovered that NSUN2, ALYREF, and YBX1 form a tripartite complex (Fig. 5a) that modulates *YAP* mRNA interactions (Fig. 5e-g). Mechanistically, NSUN2-mediated m^5^C deposition enables ALYREF-YBX1 binding to modified *YAP* transcripts and then YBX1 presents these transcripts to AGO2, attenuate RISC assembly with miRNAs (e.g., miR-107, miR-1843a-5p) [33, 42] that target the 3’UTR of *YAP* mRNA, ultimately reducing mRNA decay (Fig. 5h–k). Consequently, the recognition of m⁵C by the ALYREF-YBX1 complex enhances the translational efficiency of *YAP* mRNA. Our findings demonstrate that YBX1 facilitates the delivery of m⁵C-modified *YAP* mRNA to eIF3a-containing initiation complexes, thereby promoting protein synthesis (Fig. 5l-n). Thus, YAP upregulation occurs through dual mechanisms: enhanced mRNA stability and facilitated translation initiation in NSCLC. Notably, our findings reveal a novel positive feedback loop in which YAP-TEAD2 transcriptionally activates *NSUN2* expression (Fig. 6), which in turn amplifies YAP signaling to drive tumor cell proliferation, migration, and EMT. In NSCLC, the YAP-TEAD2-NSUN2 feedforward signaling axis sustains *YAP* transcriptional activity, driving NSCLC progression by upregulating YAP target genes including *CTGF*, *CYR61*, *MMP2*, and *MMP9*, which collectively promote tumor growth and metastatic dissemination. Furthermore, our result showed that the YBX1 inhibitor decreased the expression of YBX1 (Fig. 8c). However, the NSUN2 inhibitor only impeded the RNA catalytic activity but not affected its expression (Fig. 8j, k). Intriguingly, the inhibitors of NSUN2 and YBX1 synergistically impede NSCLC tumor growth and metastasis by regulation of YAP and then restrained the tumor growth and metastasis in vivo. This dual regulatory strategy offers distinct advantages. NSUN i suppresses NSUN2 catalytic activity, significantly reducing m⁵C modification on *YAP* mRNA. This decrease in m⁵C levels destabilizes *YAP* mRNA, leading to diminished *YAP* transcript abundance. On the other hand, SU056 downregulates YBX1 expression. Since YBX1 interacts with eIF3a, its reduction inhibits *YAP* translation, consequently lowering YAP protein levels. Together, these effects synergistically reduce *YAP* expression, ultimately suppressing NSCLC growth and metastasis. However, the NSUN2 and YBX1 inhibitors were initially identified from the ChemDiv compound library and have been found to specifically inhibit NSUN2 methyltransferase activity by targeting its catalytic domain and significantly reduce the YBX1 protein level, respectively. To date, there is no publicly available record indicating that these compounds have been clinically approved by regulatory authorities or enrolled in any phase I/II/III clinical trials, as their development is currently in the preclinical research stage. Their current applications are limited to basic research, particularly in investigations of YBX1/NSUN2-dependent biological processes, such as cancer cell proliferation and metastasis. This study provides a theoretical foundation for the clinical application of NSUN2 and YBX1 inhibitors and serves as a driving force to accelerate their translation into clinical practice. It should be noted that previous studies show verteporfin (VP), a YAP inhibitor, suppresses NSCLC malignancy by inhibiting proliferation, migration, invasion, and EMT [43]. Whether VP, SU056, and NSUN2i exhibit synergistic anti-tumor efficacy remains unclear and requires further exploration. In summary, the above research indicates that NSUN2 catalyzes m⁵C deposition on *YAP* RNA, enabling ALYREF recognition of this modification and then YBX1 binds the m⁵C-ALYREF-*YAP* mRNA complex, stabilizing *YAP* mRNA to enhance protein expression and drive oncogenic proliferation and metastasis in NSCLC (Fig. 9).

The YAP pathway is an evolutionarily highly conserved signaling pathway, which can regulate organ size via regulating stem cell proliferation, apoptosis, self-renewal ability and play an important role in tumor occurrence and organ regeneration [44]. Unlike other conventional signaling pathways, the YAP pathway has specific ligand-receptor pairs that allow it to respond to a variety of biochemical, physical, and structural signals such as cell polarity, cell contact, cell adhesion, cell density, hormones, bioactive chemicals, cell stress, and metabolic signals [29]. While recent investigations have predominantly focused on elucidating novel molecular components and alternative regulatory modalities within the YAP pathway, the intrinsic regulatory mechanisms governing YAP itself, the central effector of this pathway, remain poorly understood. So, the mechanism regarding YAP activation, nuclear translocation, and downstream transcriptional regulation need to be further explored. Therefore, elucidating the YAP signaling pathway may uncover fundamental oncogenic mechanisms, guide therapeutic strategies, and reveal novel therapeutic targets for NSCLC.

In conclusion, we observed that YAP expression is positively correlated with NSUN2 expression, and that these two proteins play similar roles in the regulation of NSCLC tumor growth and metastasis. We demonstrate that NSUN2, ALYREF and YBX1 bind to each other and affected their interaction with *YAP* mRNA. Notably, NSUN2 increases the level of m^5^C modification on *YAP* mRNA. In the context of tumor development, m^5^C-modified *YAP* mRNA is first recognized by ALYREF and then YBX1 binds to m^5^C-ALYREF-*YAP* mRNA complex to regulate *YAP* stability through impeding the interaction between AGO2 and *YAP* mRNA whereby increasing the expression of *YAP* with interaction with eIF3a in an m^5^C-dependent manner. Importantly, our data showed that *NSUN2* is a direct transcriptional target of the YAP-TEAD2 complex, establishing a positive feedback loop between NSUN2 and YAP-TEAD2 in NSCLC. Furthermore, the inhibitors of NSUN2 and YBX1 synergistically impede NSCLC tumor growth and metastasis by regulation of YAP in vitro and in vivo. Consequently, higher expression of YAP promotes cellular proliferation through its target genes of *CTGF* and *Cyr61* meanwhile facilitates cellular migration and invasion growth via *MMP2* and *MMP9*, resulting in NSCLC growth and metastasis. This study provides a significant contribution to the field by demonstrating that targeting *YAP* mRNA m^5^C modification represents a novel therapeutic strategy for NSCLC.

## Supplementary information

Supplementary Information

Unedited blot and gel images

## Data Availability

Supplementary Methods and Figures are attached.
