# Supplementary material for: NSUN2 promoted tumor growth and metastatic via m5C-regulation of YAP through ALYREF/YBX1 axis in NSCLC
Source: Cell Death Dis. 2026 Mar 7;17(1):299. doi: 10.1038/s41419-025-08353-x (PMC13039413; doi:10.1038/s41419-025-08353-x)

Supplementary Information For

NSUN2 promoted tumor growth and metastatic via m^{5}C-regulation of YAP through ALYREF/YBX1 axis in NSCLC

Rui Li[1,2#], Dan Jin[2#], Shuang Shao[2#], Jiwei Guo[1,2*]

Contents

Supplementary Methods 2
- RNA isolation and reverse transcription (RT)-PCR assay 2
- Western blot analysis 2
- Immunofluorescence staining 2
- Immunohistochemical analysis 3
- Over-expression and knockdown of genes 3
- CCK8 assays 3
- Wound healing assays 4
- Transwell migration and invasion assays 4
- qPCR for m^{5}C-RIP 4
- luciferase reporter assay 5

Supplementary Tables 6
- Table S1 Primers for RT- qPCR. 6
- Table S2 Sequences for knockdown. 6

Supplementary Figure Legends 7

Supplementary Figures 10
- Supplementary Figure S1 10
- Supplementary Figure S2 11
- Supplementary Figure S3 12
- Supplementary Figure S5 14
- Supplementary Figure S6 15
- Supplementary Figure S7 16

Supplementary Methods

RNA isolation and reverse transcription (RT)-PCR assay

We used TRIzol reagent (TransGen Biotech, Beijing, China) to isolate total RNA from the samples. RNA was reverse transcribed into first-strand cDNA using a TransScript All-in-One First-Strand cDNA Synthesis Kit (TransGen Biotech). cDNAs were used in RT-PCR and quantitative real-time PCR assay with the human *GAPDH* gene as an internal control. The RT-PCR reaction mix contained 10 μL 2xMix, Amplification was performed as follows: a denaturation step at 94 ˚C for 5 min, followed by 35 cycles of amplification at 94 ˚C for 30 sec, 58 ˚C for 30 sec and 72 ˚C for 30 sec. The reaction stopped at 25 ˚C for 5 min. We got the images of RT-PCR by Image Lab™ Software (ChemiDocTM XRS+, Bio-Rad). Primers for qPCR shown in Table S1.

Western blot analysis

Human lung cancer cells were transfected with the relevant plasmids and cultured for 48 h. For western blot analysis, cells were lysed in NP-40 buffer (10 mM Tris pH 7.4, 150 mM NaCl, 1% Triton X-100, 1 mM EDTA pH 8.0, 1 mM EGTA pH 8.0, 1 mM PMSF, and 0.5% NP-40) at 25 ˚C for 20 min. The lysates were added to 5 × loading dye and then separated by electrophoresis. The primary antibodies used in this study were 1:1000 Abcam (Cambridge, UK) antibody of YAP (ab52771), NSUN2 (ab259941), ALYREF (ab202894), YBX1 (ab255606), CTGF (ab6992), Cyr61 (ab24448), Vimentin (ab45939), E-cadherin (ab1416), cleaved Capase-3 (ab32042) and Tubulin (ab6046), GAPDH (ab181602).

Immunofluorescence staining

Cells were seeded in 24-well plates at $5x10^3$ per well. After cellular transfection 48 h, cells were fixed as previously described. Cells were incubated with primary antibodies overnight at 4°C. As a negative control, slides incubated with BSA were stained in parallel. Then cells were incubated with the secondary

antibody Alexa Fluor 594 AffiniPure Goat Anti-Rabbit IgG or Alexa Fluor 488 AffiniPure Goat Anti-Mouse IgG diluted with BSA for 2 h at 25 °C. Cells were counterstained with 4',6-diamidino 2-phenylindole (DAPI) and visualized with an advanced fluorescence microscope (Olympus BX63) and quantified using ImageJ software.

Immunohistochemical analysis

Tumor tissues were fixed in 4% paraformaldehyde overnight and then embedded in paraffin wax. Four-micrometer thick sections were stained using hematoxylin and eosin (H&E) for histological analysis.

Over-expression and knockdown of genes

Overexpressing plasmid (2 μg), siRNA (1.5 μg) and shRNA (1.5 μg) of indicated genes were transfected into cells using Lipofectamine 3000 (Invitrogen, Carlsbad, CA) for over-expression and knockdown of indicated genes, followed by analysis 48–72 h later. In our experimental design, we implemented stringent control measures for both gene overexpression and targeted knockdown experiments. Specifically, the pcDNA-3.1 (Vector) served as the negative control for gene overexpression, while the shNC acted as the negative control for the gene knockdown. To ensure the specificity and reliability of our results, we co-transfected the overexpression or knockdown constructs with their corresponding control vectors in our experiments. This experimental approach allowed us to systematically assess the effects of gene overexpression or knockdown while effectively minimizing experimental variables and ensuring solid data interpretation. The selected sequences for knockdown are shown in Table S2.

CCK8 assays

Cell viability and growth was determined using CCK8 assays in 96-well plates in a manner. Cells were transfected with the relevant plasmids culturing for 36 h, followed by incubation with CCK8 for 2 h. Absorbance was read at 450 nm

using a spectrophotometer (Tecan, Männedorf, Switzerland).

Wound healing assays

To assess the cellular migration, 10^5 cells were seeded onto 6-well plates with transfection of the relevant plasmids. These were then incubated in 5% CO_2 at 37 ˚C for 48 h. A wound was scraped into the cells using a plastic 200 μL tip and then washed by PBS. The cells were then incubated in RPMI-1640 medium containing 2% FBS. Images were captured at the time points of 0 and 36 h after wounding. The relative distance of the scratches was observed under an optical microscope (IX53, Olympus, Tokyo, Japan) and assessed using the ImageJ software.

Transwell migration and invasion assays

Transwell migration assays were performed using a 24-well chamber (Costar 3422; Corning Inc., Corning, NY, USA). The lower and upper chambers were partitioned by a polycarbonate membrane (8-μm pore size) for migration and coated with Corning®Matrigel® for invasion. Lung cancer cells (1×10^3) were seeded into RPMI-1640 without FBS in the upper chamber. RPMI-1640 containing 10% FBS was added to the lower chamber. The cells were allowed to migrate for 36 h at 37 ˚C in a humidified atmosphere containing 5% CO_2. Cells remaining on the upper side of the membrane were removed using PBS-soaked cotton swabs. The membrane was then fixed in 4% paraformaldehyde for 20 min at 37 ˚C and then stained with crystal violet. Cells on the lower side of the membrane were counted under an Olympus light microscope (Olympus, Tokyo, Japan).

qPCR for m^5C-RIP

Reverse transcription was performed on 10 μL m^5C PolyA+ RNA from the MeRIP with the iScript cDNA synthesis kit (Bio-Rad Laboratories, Hercules, CA). After diluting cDNA two-fold, quantitative real-time PCR was performed using the CFX96™ Real-Time PCR System (1855195, Bio-Rad) and primers from Integrated DNA Technologies, Inc. (Coralville, Iowa). Primer efficiency was verified to be over 95% for all primer sets used. Quantification of mRNA from the m^5C-RIP was carried out via $2^{-\Delta\Delta CT}$ analysis against non-

immunoprecipitated input RNA. All real-time PCR primer sets were designed so the products would span at least one intron (> 1 kb when possible), and amplification of a single product was confirmed by agarose gel visualization and/or melting curve analysis.

luciferase reporter assay

To construct the core region of *NSUN2* promoter, the region of *NSUN2* was amplified by PCR from the human cDNA of A549 cells and were inserted into the upstream of the pGL3-Basic vector (Promega, Madison, WI, USA) via KpnI and XhoI sites to generate *NSUN2* luc. Thereafter, we use the Firefly Luciferase Reporter Gene Assay Kit (#RG005, Beyotime, China) to detect the promoter activities. The PGL3-basic plasmid was used as a negative control. Data was normalized against Renilla luciferase activity.

Supplementary Tables

Table S1 Primers for RT- qPCR.

Genes	Forward	Reverse
NSUN2	GGTCAACCATGATGCCTCCA	GCGTCCCAGTCTGTAAACCA
ALYREF	GCAGGCCAAAACAACTTCCC	AGTTCCTGAATATCGGCGTCT
YBX1	AGGCAGGAACGGTTGTAGGT	CCTTGTTCTCCTGCACCCTG
YAP	GGACCCCAGACGACTTCCTC	CCTTCCAGTGTGCCAAGGTC
Cyr61	GGTCAAAGTTACCGGGCAGT	GGAGGCATCGAATCCCAGC
CTGF	ACCGACTGGAAGACACGTTTG	CCAGGTCAGCTTCGCAAGG
E-cadherin	ACCATTAACAGGAACACAGG	CAGTCACTTTCAGTGTGGTG
Vimentin	CGCCAACTACATCGACAAG	CTGGTCCACCTGCCGGCG
GAPDH	CTCCTCCTGTTCGACAGTCA	CCCAATACGACCAAATCCG

Table S2 Sequences for knockdown.

Genes	Target Sequences
shNSUN2-1	CCCAAGAATGAACGGCTTCAT
shNSUN2-2	ATAGTTGTCCCGGGACGTCA
shALYREF-1	TTGCTGAATTTGGAACGCTGAA
shALYREF-2	CGTGGAGACAGGTGGGAAACT
shYBX1-1	GACGGCAATGAAGAAGATAAA
shYBX1-2	GACAACCAGGGTGCAGGAGAA
shYAP-1	AAGGTGATACTATCAACCAAA
shYAP-2	AAGACATCTTCTGGTCAGAGA
sieIF3a	CAGTTGATGGCAAATTACT
shNC	TTCTCCGAACGTGTCACGA

Supplementary Figure Legends

Figure S1. NSUN2 and YAP play a similar role in NSCLC cells growth, migration and EMT.

(a) The association between the expression of NSUN2/YAP and the progression grading of lung adenocarcinoma and lung squamous cell carcinoma. **(b)** mRNA levels of *NSUN2* and *YAP* in NSCLC cell lines. **(c)** m^5C methylation assay in NSCLC samples (n=10). **(d)** *NSUN2* and *YAP* knockout validation. **(e-h)** protein levels of NSUN2 and YAP **(e)**, cellular growth **(f)**, immunofluorescent staining of Cleaved-Caspase 3 **(g)** and Annexin V **(h)** in transfected A549 cells. **(i)** The number of colons were analyzed by colony formation assay. **(j-k)** cellular migration **(j)** and EMT **(k)** in A549 and H1299 cells. Results were presented as mean ± SD of three independent experiments. $^*P<0.05$, $^{**}P<0.01$ *VS* Control.

Figure S2. NSUN2 promoted cellular invasion and EMT via regulation of YAP in the NSCLC cells.

(a) dot blot assay of m^5C methylation in transfected A549 cells. **(b)** Putative m^5C modification sites in the mRNA sequence of *YAP* and synonymous mutations in the *YAP* mRNA (created with BioRender.com). **(c-e)** RNA pulldown (**c, d**) and RIP (**e**) assay of interaction between NSUN2/Flag-NSUN2 and *YAP* mRNA using the Primer 1 (P1) detected the S1 site and Primer 2 (P2) detected the S2 site. (**f, g**) m^5C-RIP-qPCR assay of m^5C methylation. (**h, i**) relative mRNA levels of *YAP* in the transfected H1299 and A549 cells. (**j**, **k**) RIP (**j**) and RNA pulldown (**k**) assays of the interaction between NSUN2 WT/KD and *YAP* mRNA. **(l, m)** the cellular viability **(l)** and colon formation growth **(m)** in A549 cells. **(n)** The stability of *YAP* mRNA in transfected A549 cells. **(o, p)** cellular invasion growth **(o)** and EMT **(p)** in transfected A549 and H1299 cells. Results were presented as mean ± SD of three independent experiments. $^*P<0.05$, $^{**}P<0.01$ or $^{\#}P<0.05$, $^{\#\#}P<0.01$ indicates a significant difference between the

indicated groups.

Figure S3. ALYREF and YBX1 promoted cellular migration growth and EMT via regulation of YAP in the NSCLC cells.

(a) protein levels of ALYREF and YBX1 in NSCLC cell lines. **(b-f)** mRNA levels of *ALYREF* and *YBX1* **(b)**, cellular viability **(c)**, migration **(d)**, colon formation growth **(e)** and EMT **(f)** in transfected A549 cells. **(g)** The positive correlation between YBX1 and ALYREF from TCGA database. **(h)** RNA pulldown assay of the interaction between ALYREF/YBX1 and *YAP* mRNA. **(i-o)** stability of *YAP* mRNA **(i)**, mRNA levels of *YAP* **(j)**, cellular viability **(k)**, growth **(l)**, migration growth **(m, n)** and EMT **(o)** in transfected A549 cells determined by qPCR, CCK8, wound healing, transwell and RT-PCR assays. Results were presented as mean ± SD of three independent experiments. $^{**}P<0.01$ or $^{\#\#}P<0.01$ indicates a significant difference between the indicated groups.

Figure S4. NSUN2 regulated cell growth and metastasis through m^{5}C-mediated ALYREF/YBX1 axis in NSCLC.

(a-e) cellular growth **(a)**, colon formation growth **(b)**, cellular invasion growth **(c)**, mRNA levels of *MMP2* and *MMP9* **(d)** and EMT **(e)** in A549 cells. **(f)** mRNA level of *YAP* in RNA pulldown assay using the MS2 coat protein system. Results were presented as mean ± SD of three independent experiments. $^{**}P<0.01$ or $^{\#\#}P<0.01$ indicates a significant difference between the indicated groups. *ns*, not significant.

Figure S5. NSUN2, ALYREF and YBX1 synergistically regulate cellular clone formation.

(a-c) mRNA level of *YAP* in the MS2 coat protein system used the GFP antibody. **(d-m)** mRNA levels of *YAP*, *CTGF* and *Cyr61* **(d, e)**, protein level of YAP (**f**), protein level of YAP, CTGF and Cyr61(**g**), cellular viability **(h-j)**, colon formation growth (**k, l**), cellular migration **(m)** and invasion **(n, o)** in A549 cells with transfection of relevant genes. Results were presented as mean ± SD of three

independent experiments. *P<0.05, **P<0.01 or $^{##}P$<0.01 indicates a significant difference between the indicated groups. ns, not significant.

Figure S6. NSUN2 promoted tumor growth and metastasis via increasing the expression of YAP *in vivo***.**

The protein level of NSUN2 **(a)**, YAP **(b)**, Cyr61 **(c)**, E-cadherin **(d)**, Vimentin **(e)**, Ki67 **(f)** and Cleaved Casepase-3 **(g)** were detected in xenografted A549 cell tumors with stable expression of indicated genes determined by immunohistochemical staining (*n*=5). *P<0.05, **P<0.01 or $^{##}P$<0.01 indicates a significant difference between the indicated groups.

Figure S7. SU056 and NSUN2 i synergistically impede NSCLC tumor growth and metastasis by regulation of YAP.

(a) IC_{50} of SU056 in A549 cells. **(b-f)** protein levels of YBX1 and YAP **(b)**, mRNA levels of *CTGF* and *Cyr61* **(c)**, cellular viability **(d)**, cellular invasion and migration growth **(e)** and EMT **(f)** in the SU056-treated A549 cells. **(g)** IC_{50} of NSUN2 i in A549 cells. **(h-l)** mRNA levels of *CTGF* and *Cyr61* **(h)**, cellular viability **(i)**, cellular migration and invasion growth **(j)**, EMT **(k)** and apoptosis **(l)** in NSUN2 i-treated A549 cells. **(m-p)** m^5C level **(m)**, mRNA levels of *CTGF* and *Cyr61* **(n)**, cellular viability **(o)**, colon formation growth **(p)** in the A549 cells with co-treatment with SU056 and NSUN2 i. **(q-s)** tumor weight **(q)**, overall survival **(r)** and mRNA levels of indicated genes **(s)** in xenografted A549 cell mice treated with SU056 and NSUN2 i. Results were presented as mean ± SD of three independent experiments. **P<0.01 or $^{##}P$ <0.01 indicates a significant difference between the indicated groups.

Supplementary Figures

Supplementary Figure S1

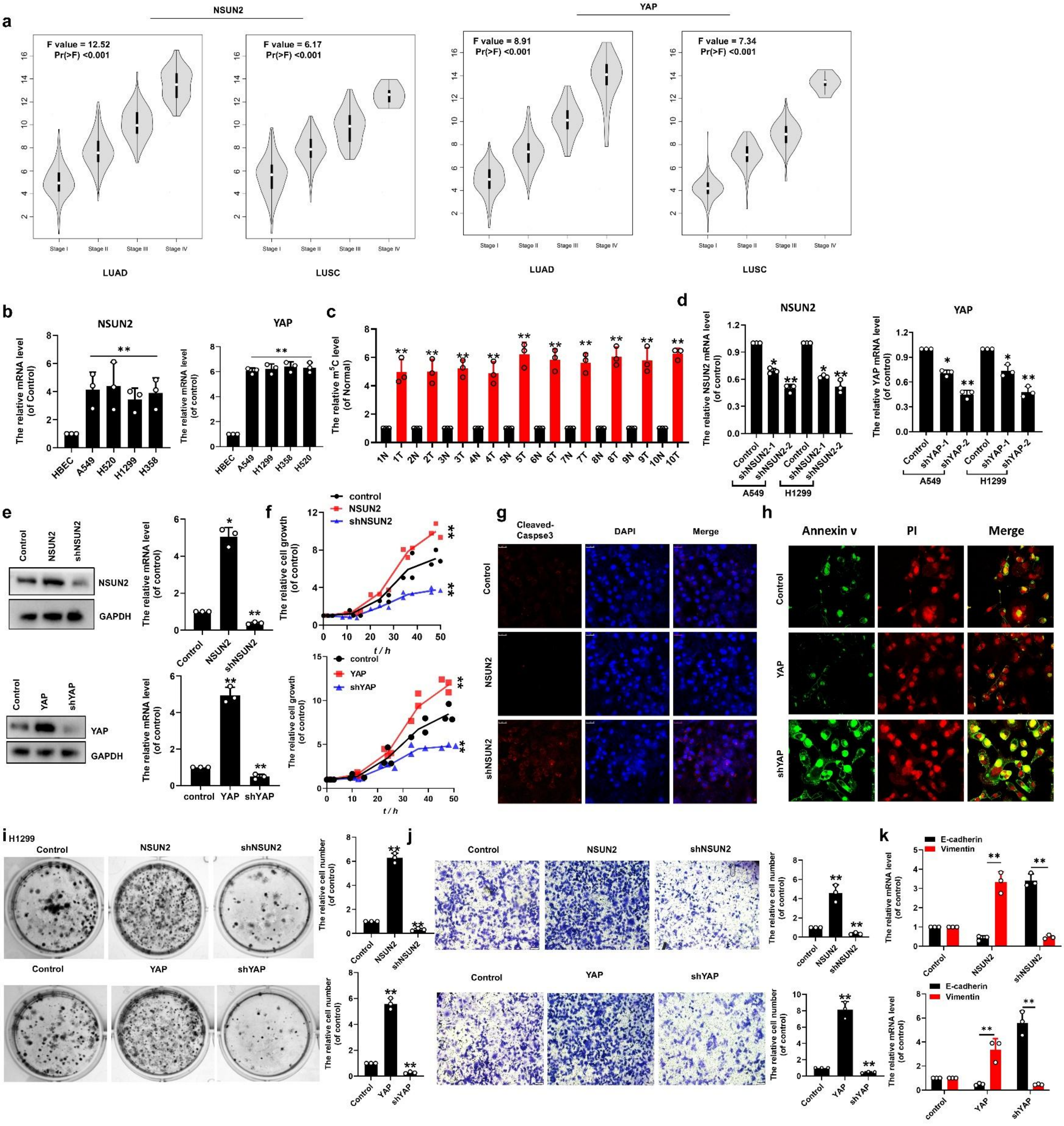

Supplementary Figure S2

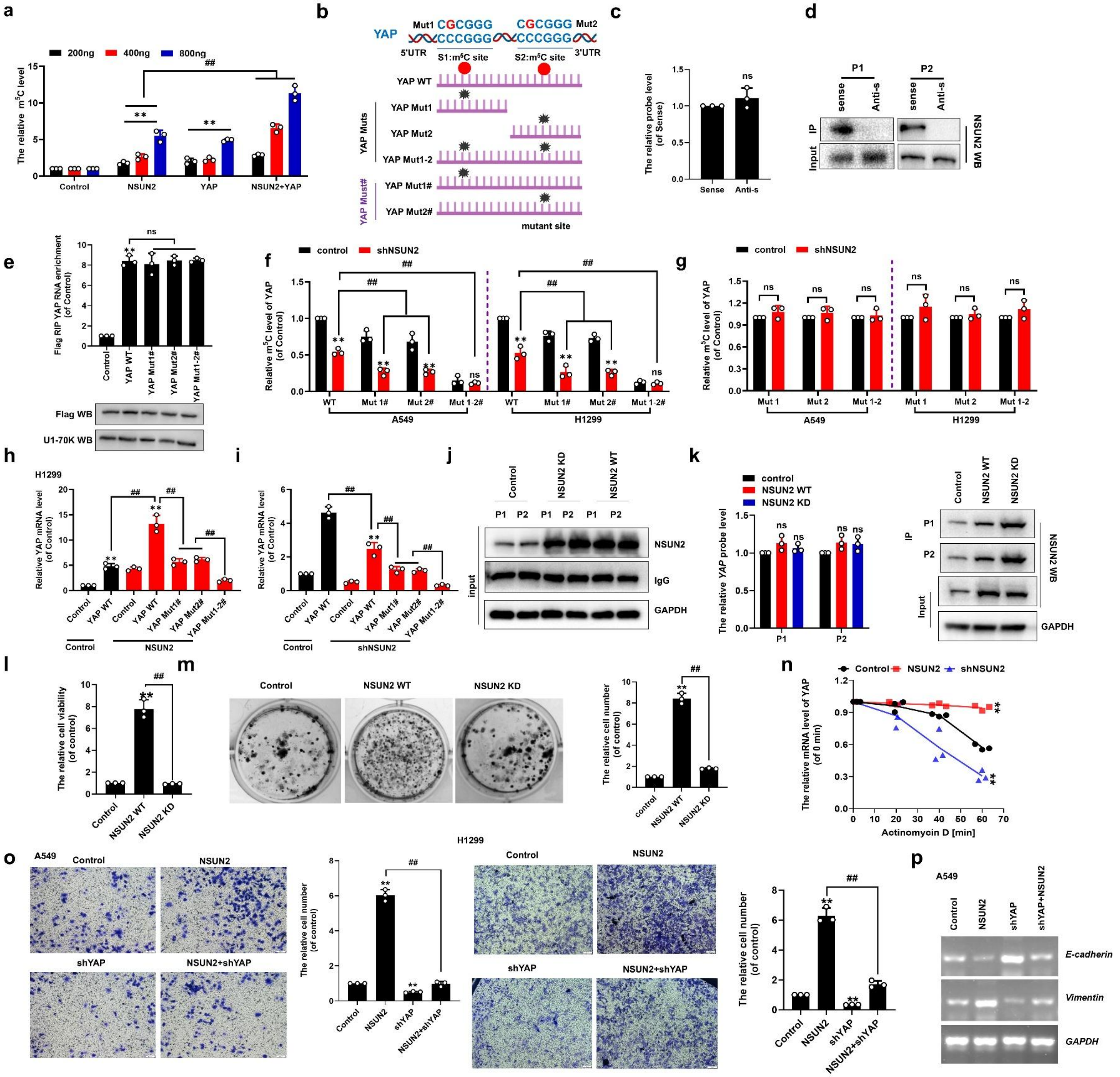

Supplementary Figure S3

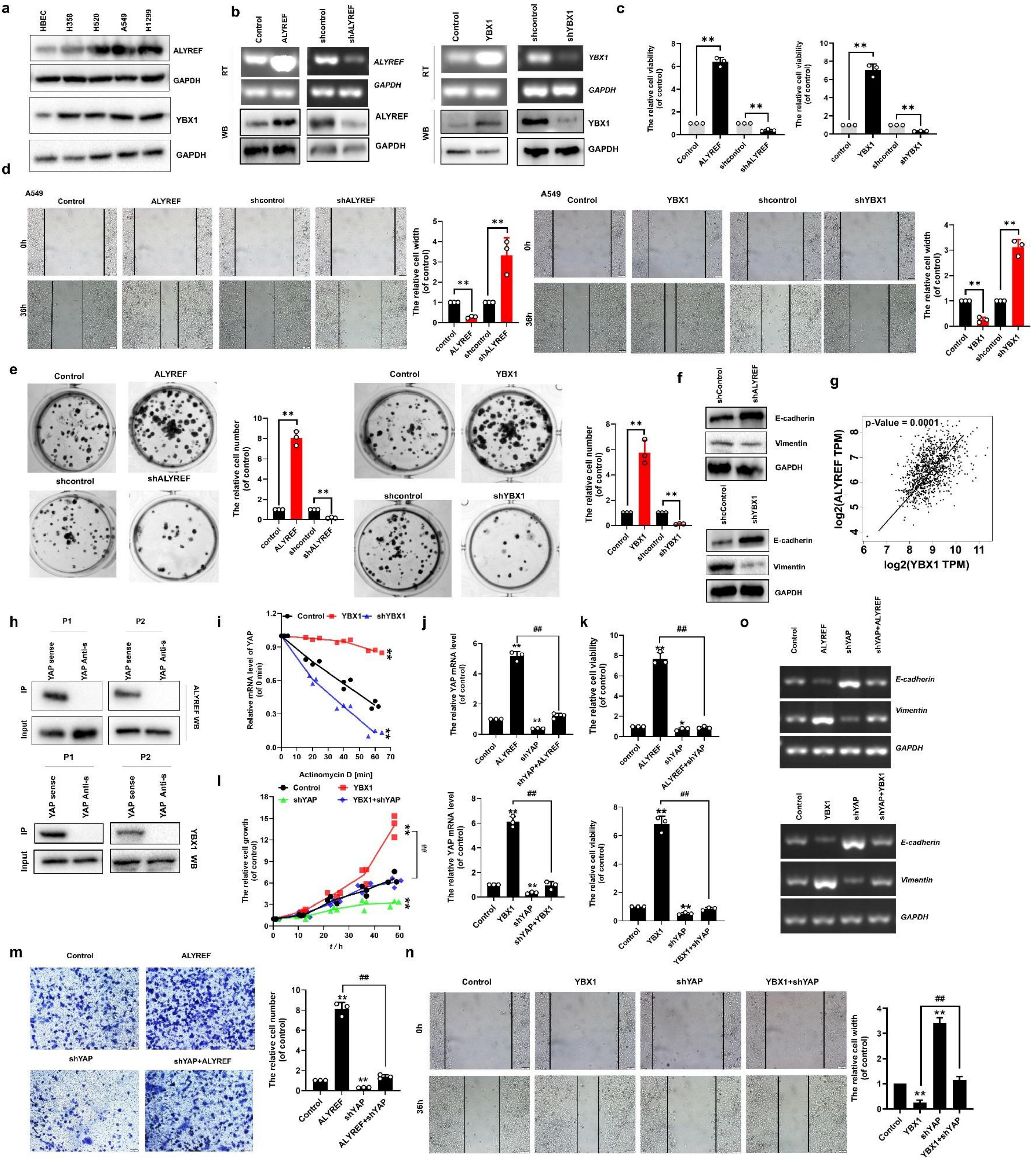

Supplementary Figure S4

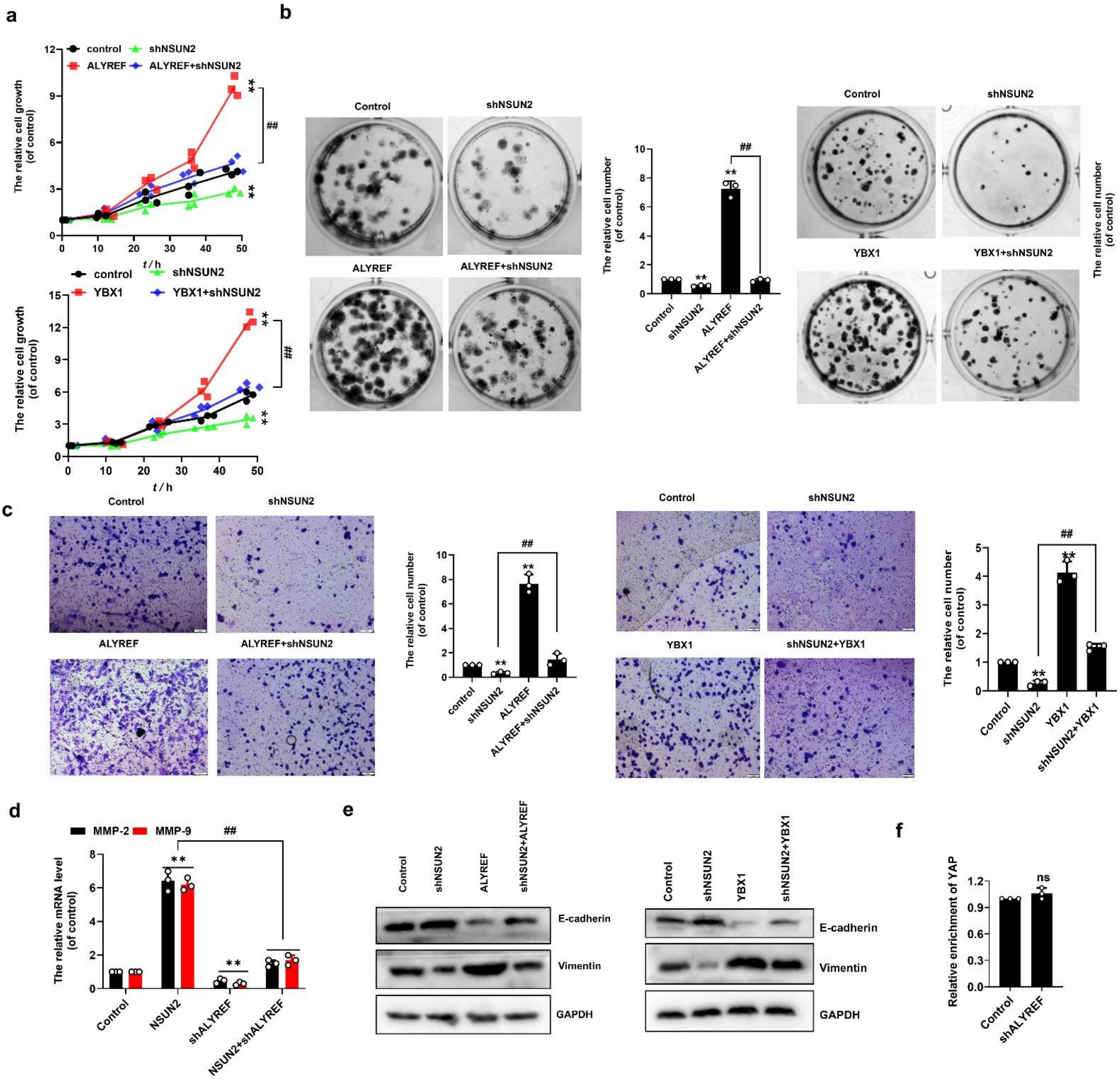

Supplementary Figure S5

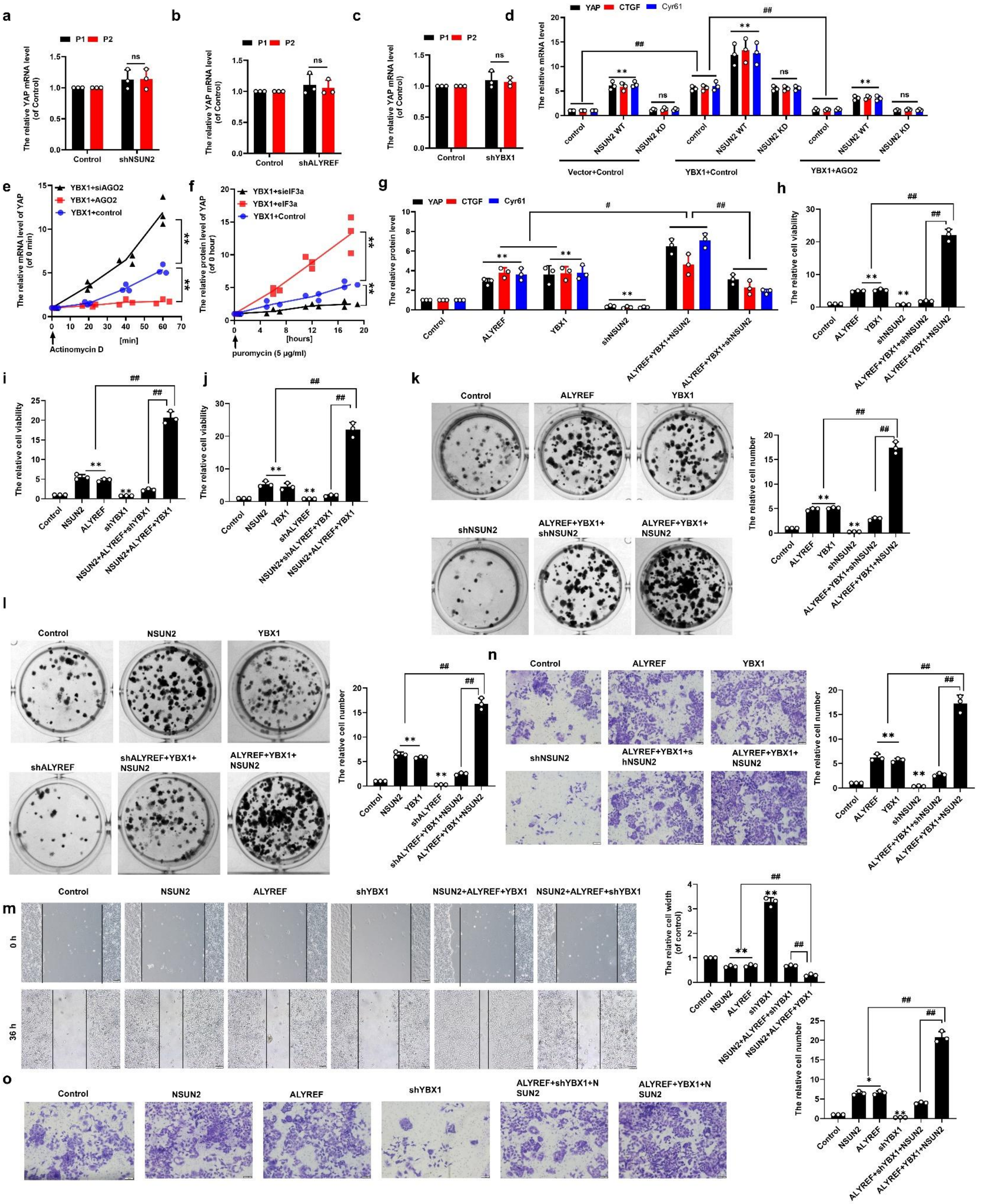

Supplementary Figure S6

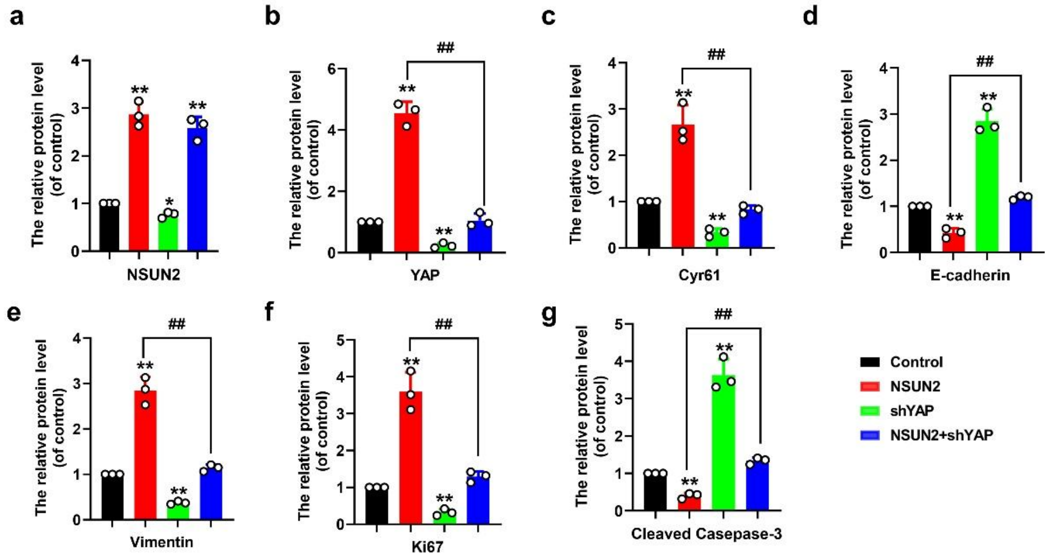

Supplementary Figure S7

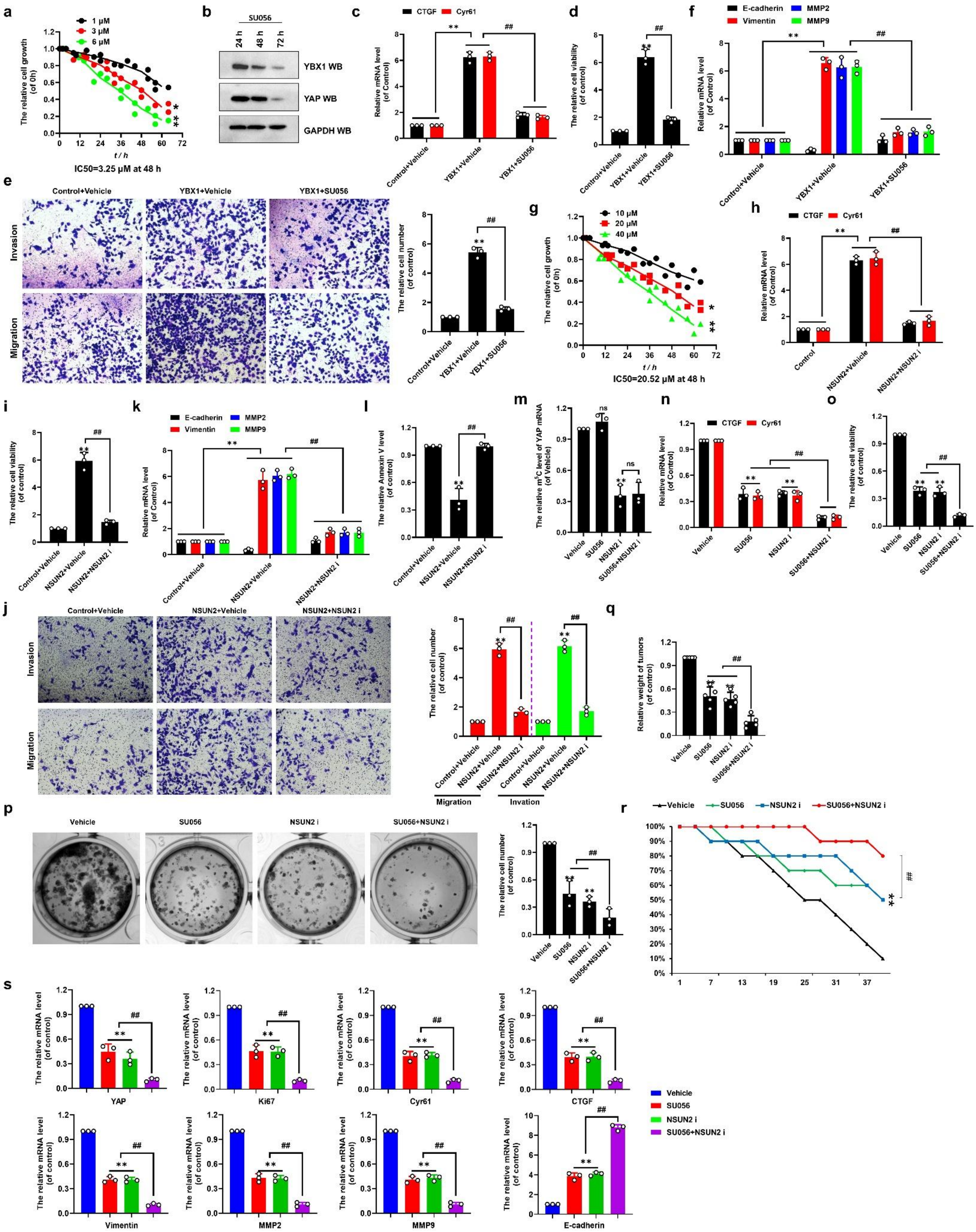

Supplement: Supplementary file 1 — Supplementary Information [file 41419_2025_8353_MOESM1_ESM.pdf]
