# Supplementary material for: NSUN2 promoted tumor growth and metastatic via m5C-regulation of YAP through ALYREF/YBX1 axis in NSCLC
Source: Cell Death Dis. 2026 Mar 7;17(1):299. doi: 10.1038/s41419-025-08353-x (PMC13039413; doi:10.1038/s41419-025-08353-x)

Fig 1

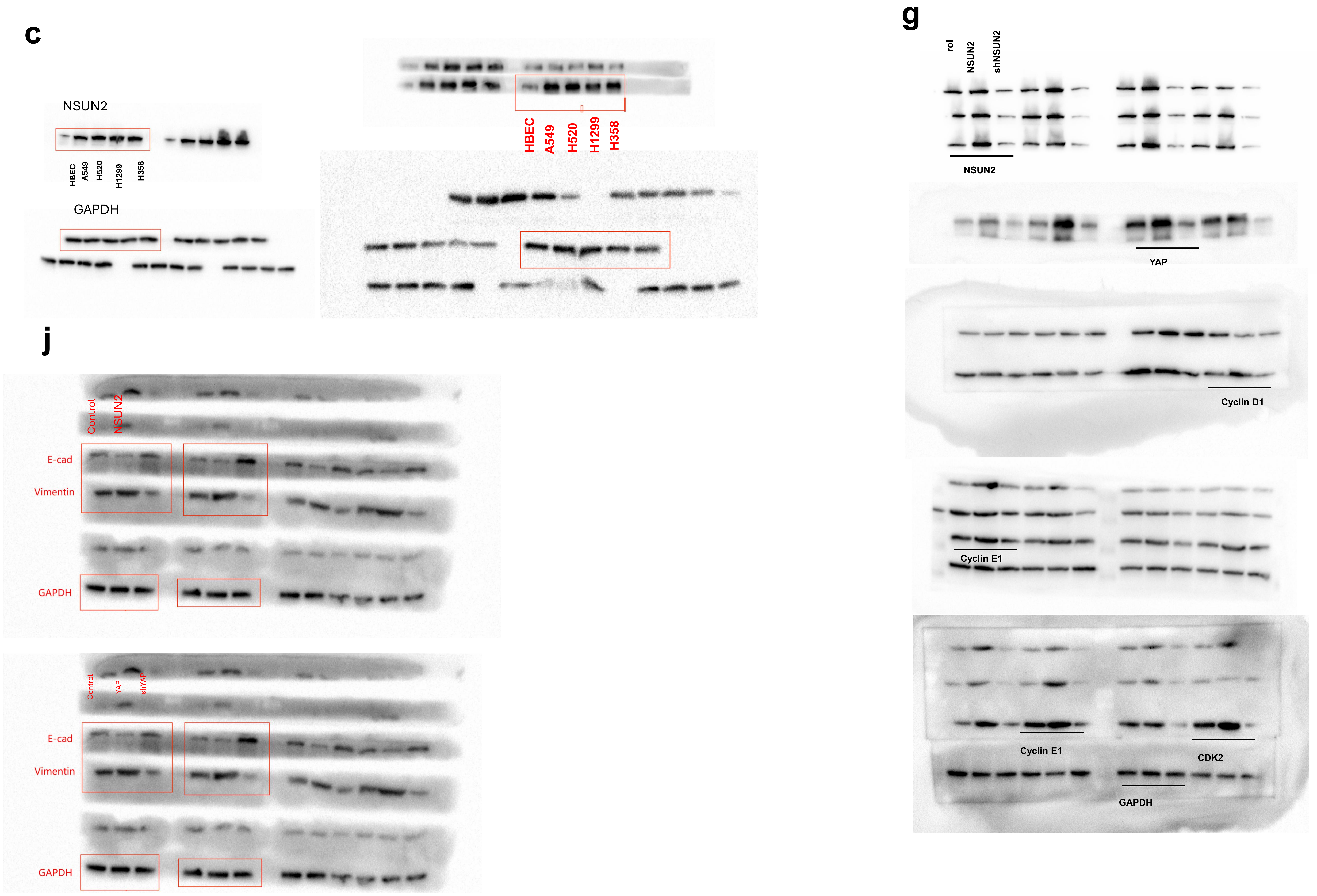
c
NSUN2
HBEC
A549
H520
H1299
H358
GAPDH
HBEC
A549
H520
H1299
H358
g
rol
NSUN2
shNSUN2
NSUN2
YAP
Cyclin D1
Cyclin E1
Cyclin E1
CDK2
GAPDH
j
Control
NSUN2
E-cad
Vimentin
GAPDH
Control
YAP
shYAP
E-cad
Vimentin
GAPDH

Fig 2

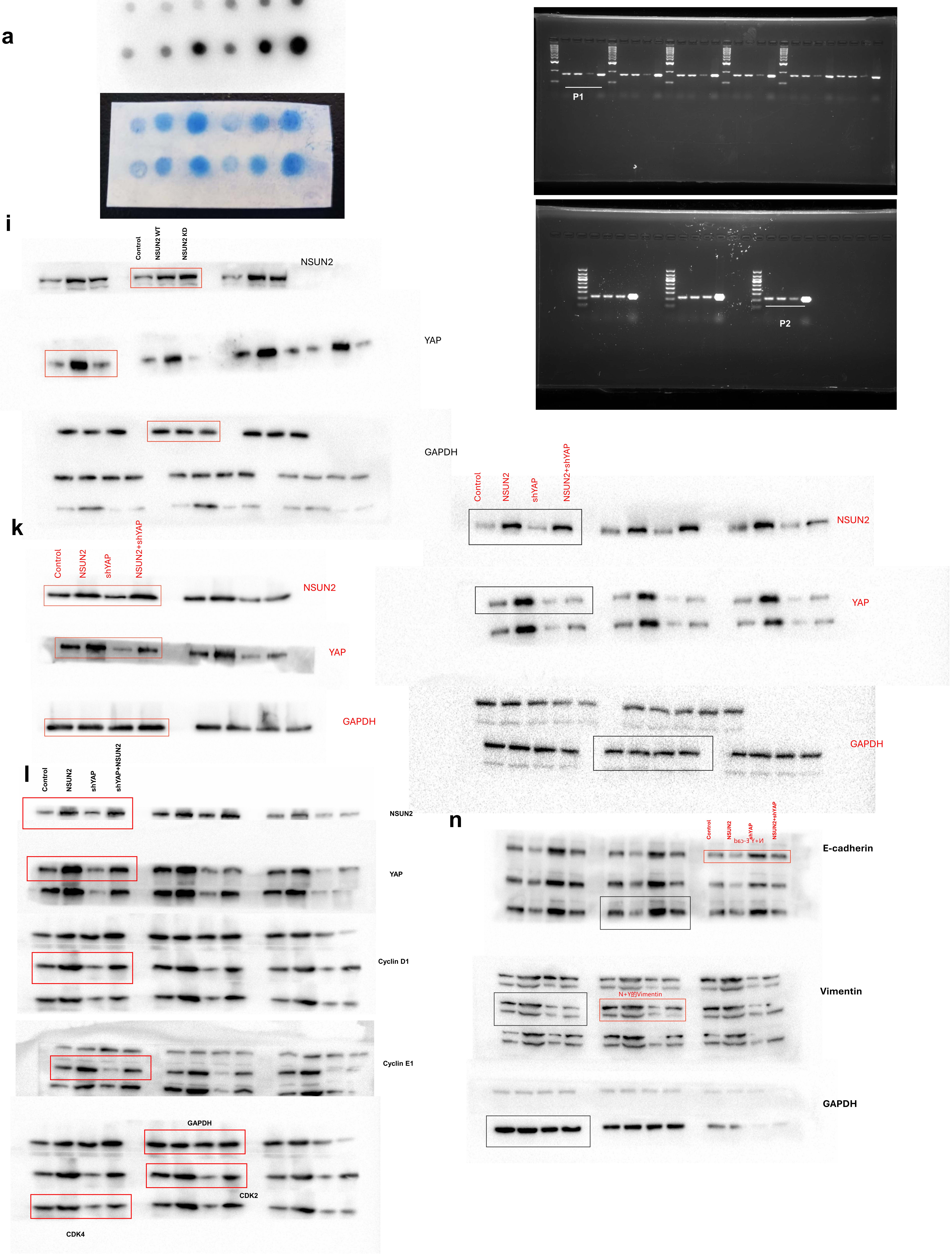
a
c
P1
P2
i
Control
NSUN2 WT
NSUN2 KD
NSUN2
YAP
GAPDH
k
Control
NSUN2
shYAP
NSUN2+shYAP
NSUN2
YAP
GAPDH
Control
NSUN2
shYAP
NSUN2+shYAP
NSUN2
YAP
GAPDH
l
Control
NSUN2
shYAP
shYAP+NSUN2
NSUN2
YAP
Cyclin D1
Cyclin E1
GAPDH
CDK2
CDK4
n
Control
NSUN2
shYAP
NSUN2+shYAP
E-cadherin
Vimentin
GAPDH

p

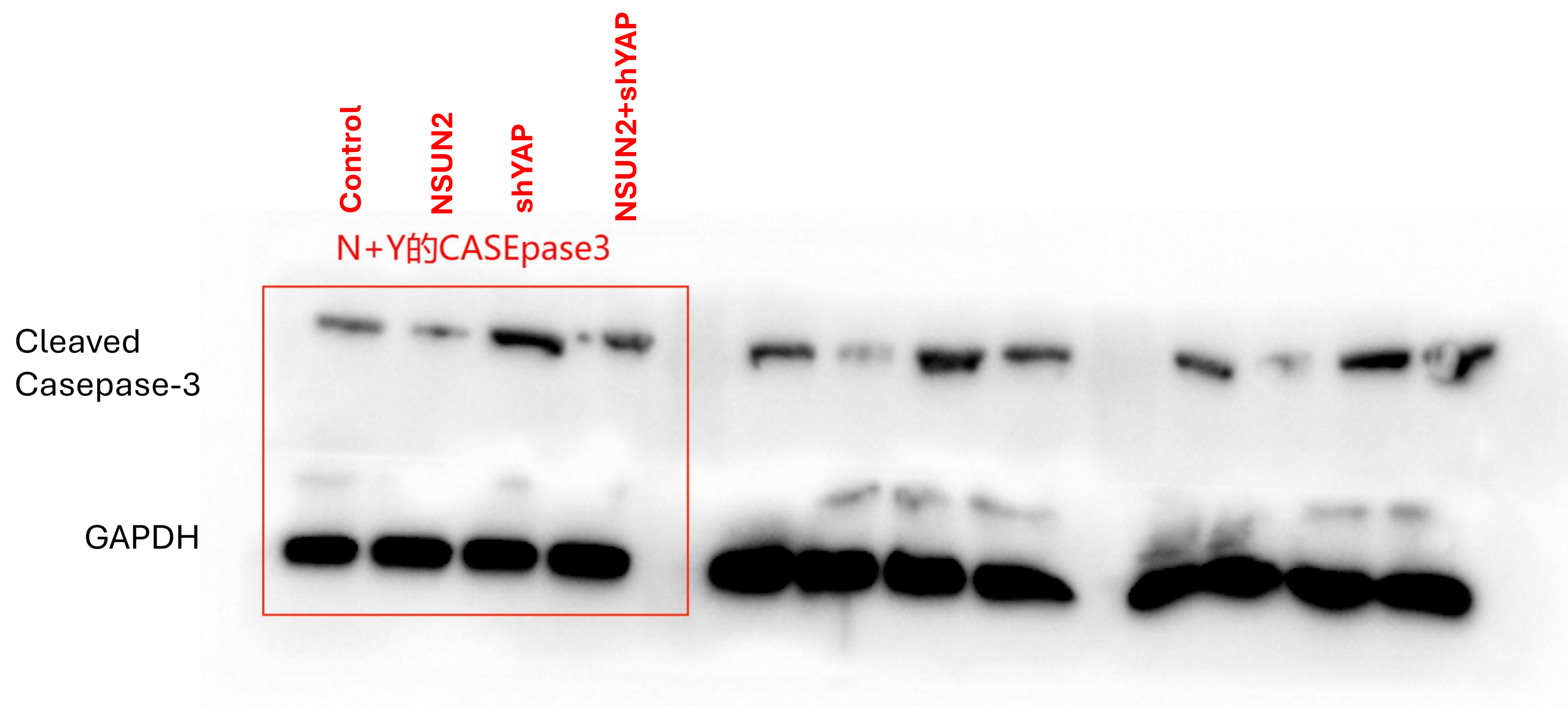
Control
NSUN2
shYAP
NSUN2+shYAP
N+Y的CASEpase3
Cleaved
Casepase-3
GAPDH

o

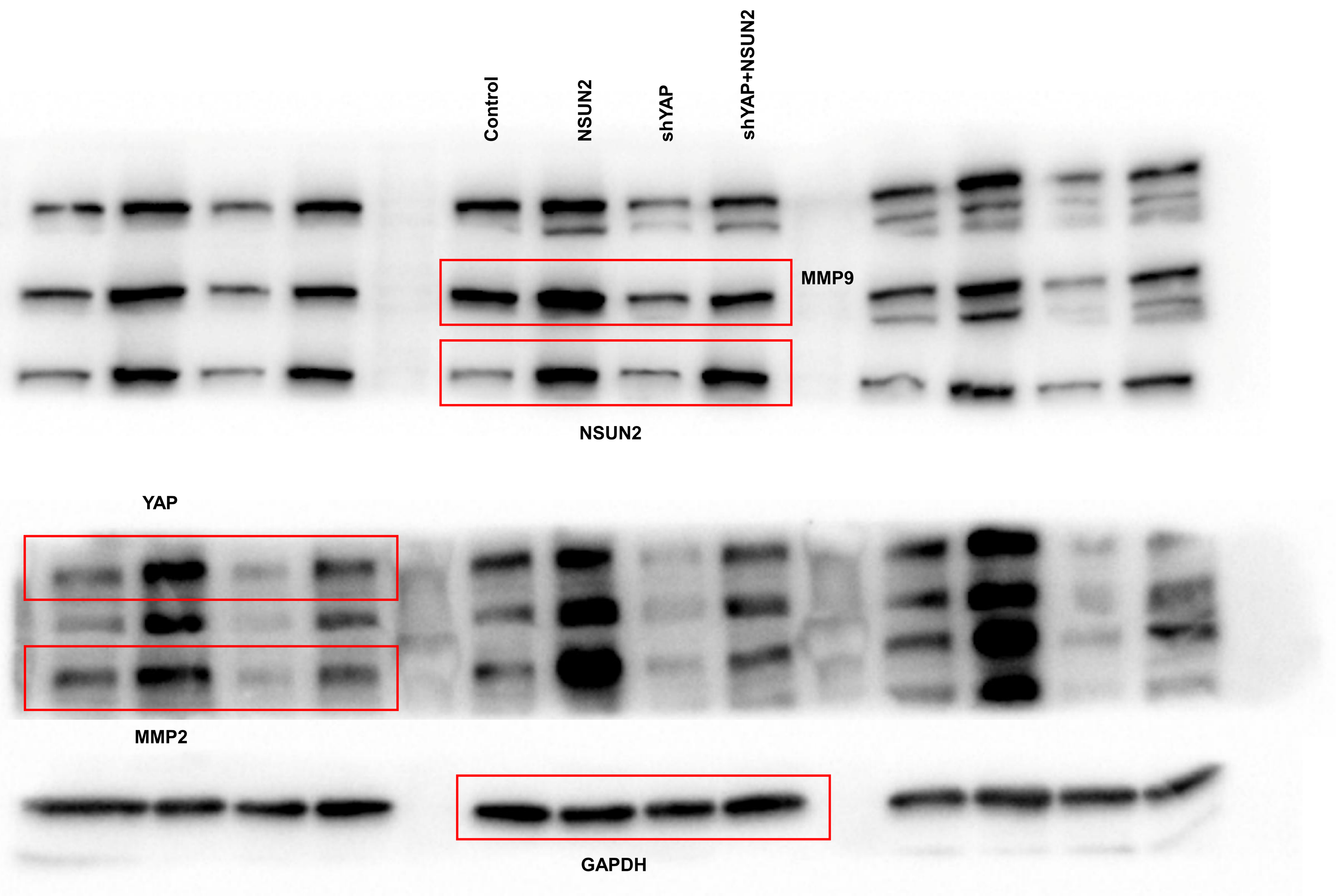
Control
NSUN2
shYAP
shYAP+NSUN2
MMP9
NSUN2
YAP
MMP2
GAPDH

Fig 3

e

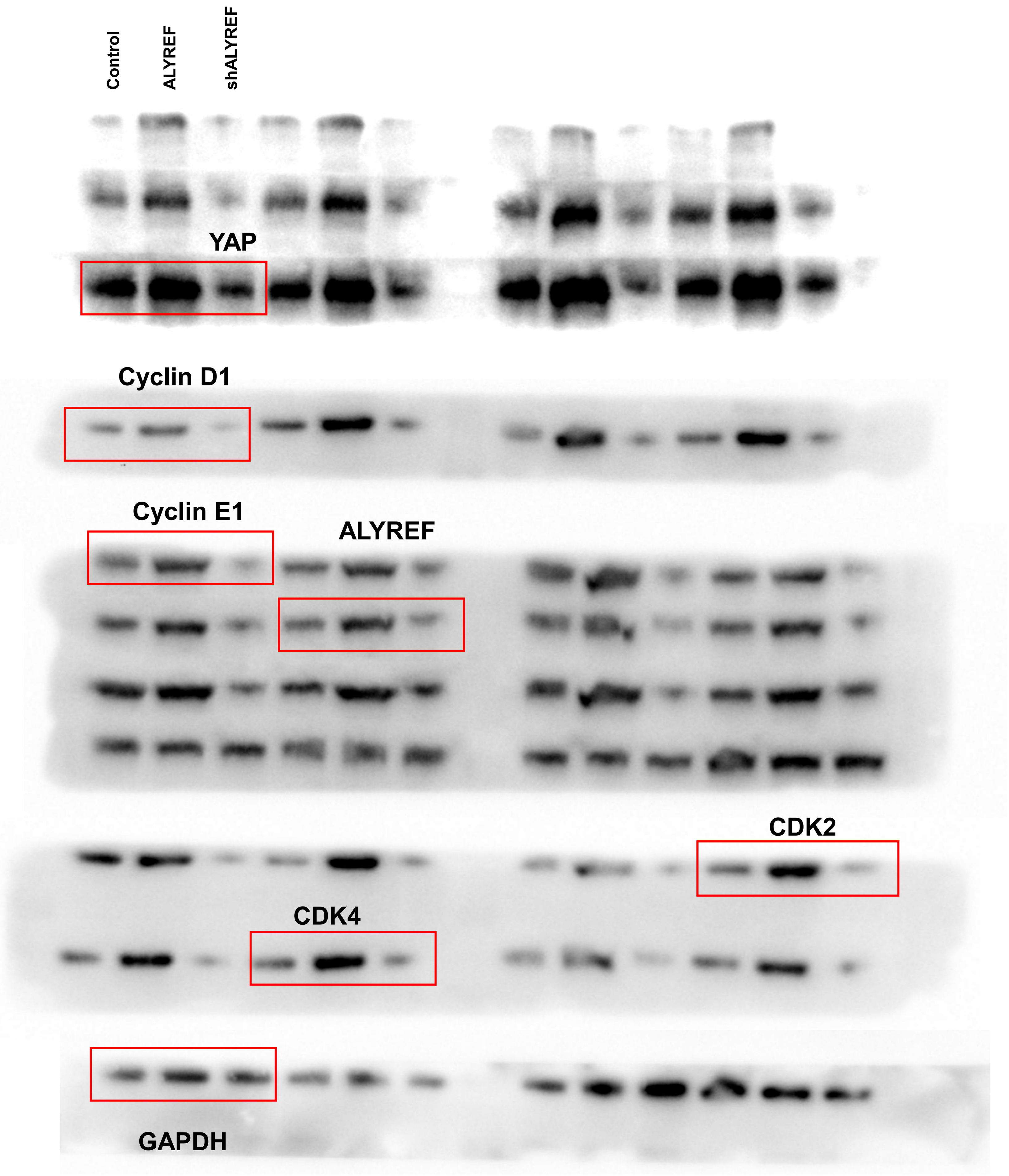
Control
ALYREF
shALYREF
YAP
Cyclin D1
Cyclin E1
ALYREF
CDK2
CDK4
GAPDH

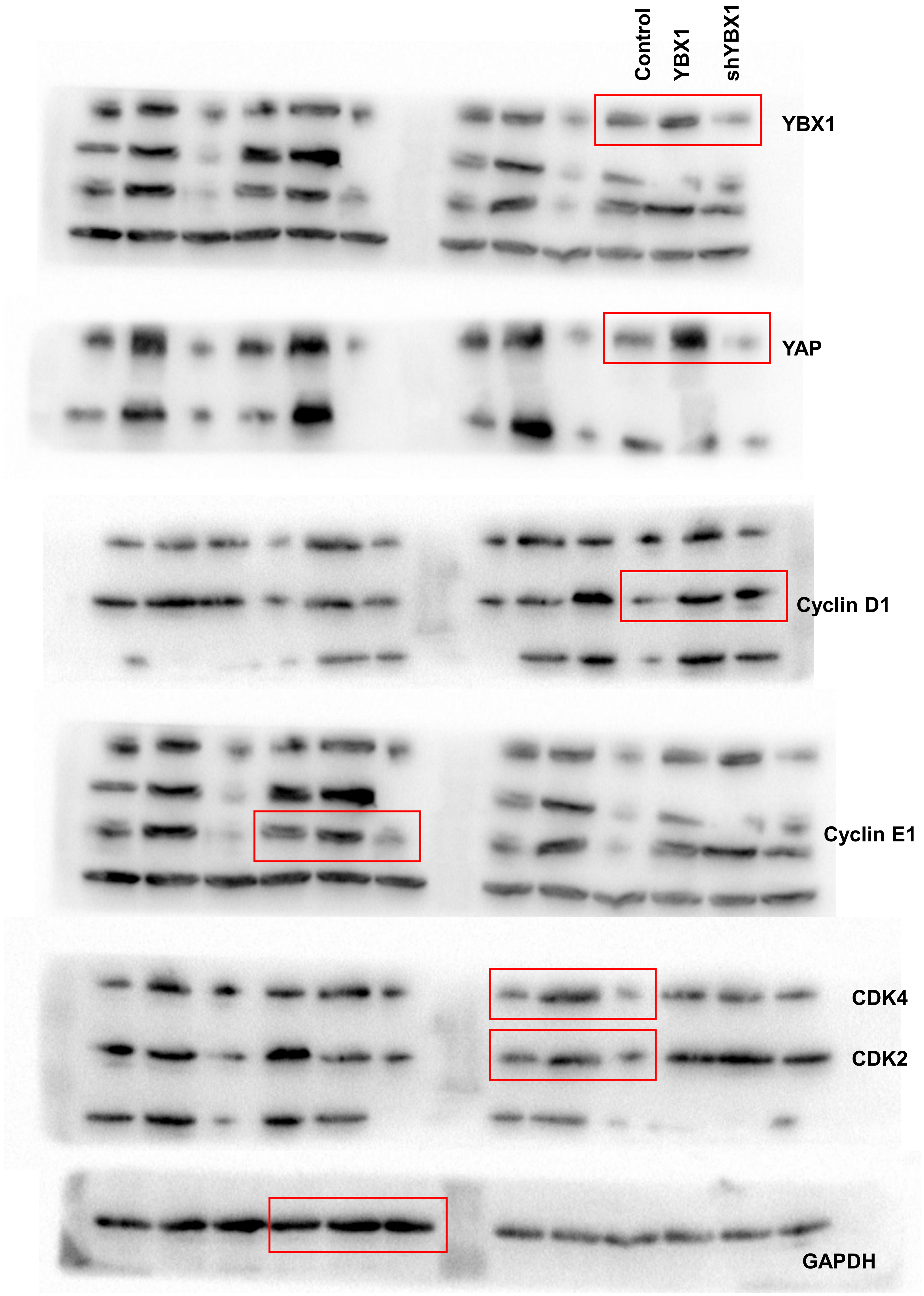
Control
YBX1
shYBX1
YBX1
YAP
Cyclin D1
Cyclin E1
CDK4
CDK2
GAPDH

g

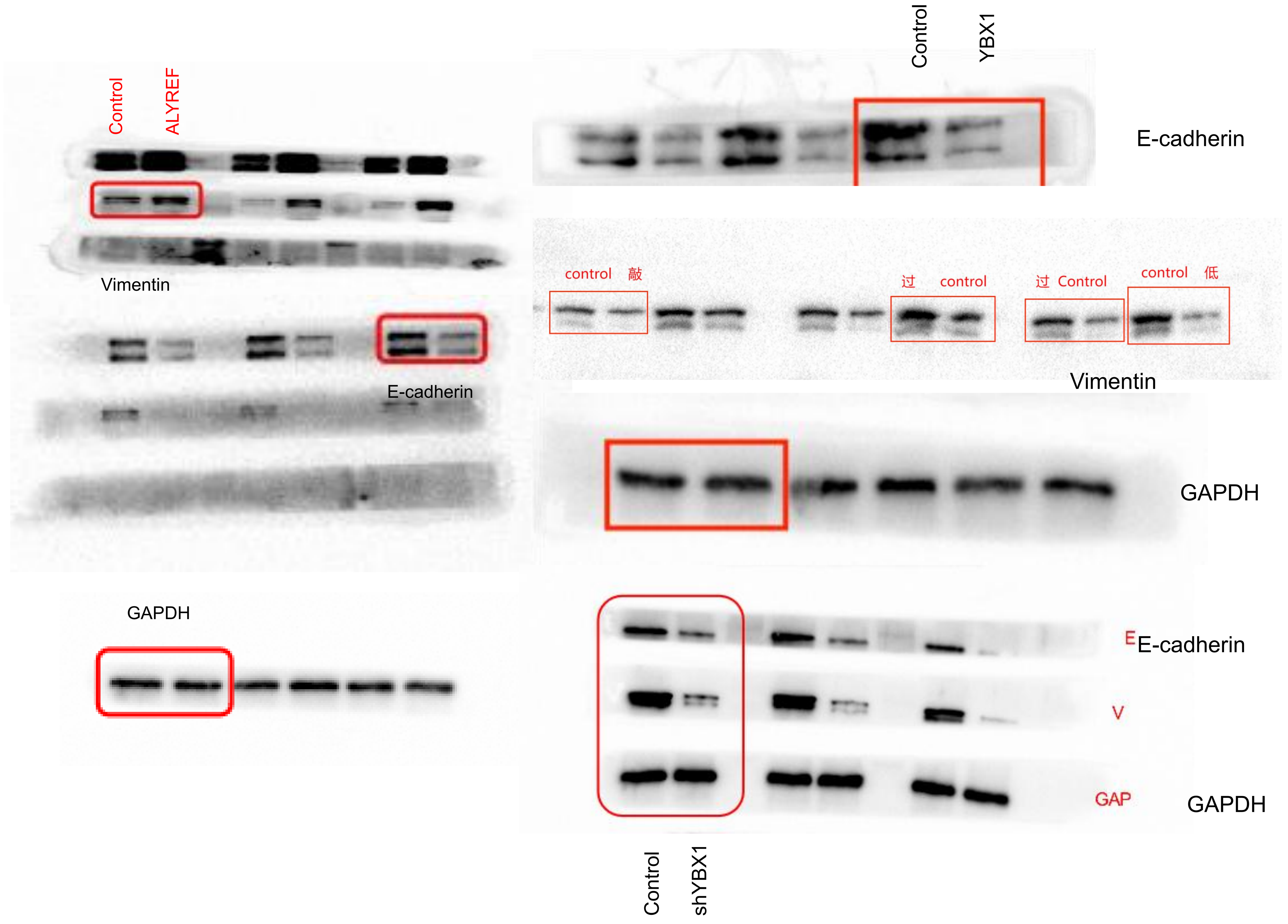

Control
ALYREF
Vimentin
E-cadherin
GAPDH
Control
YBX1
E-cadherin
control
敲
过
control
过
Control
control
低
Vimentin
GAPDH
E
E-cadherin
V
GAP
GAPDH
Control
shYBX1

h

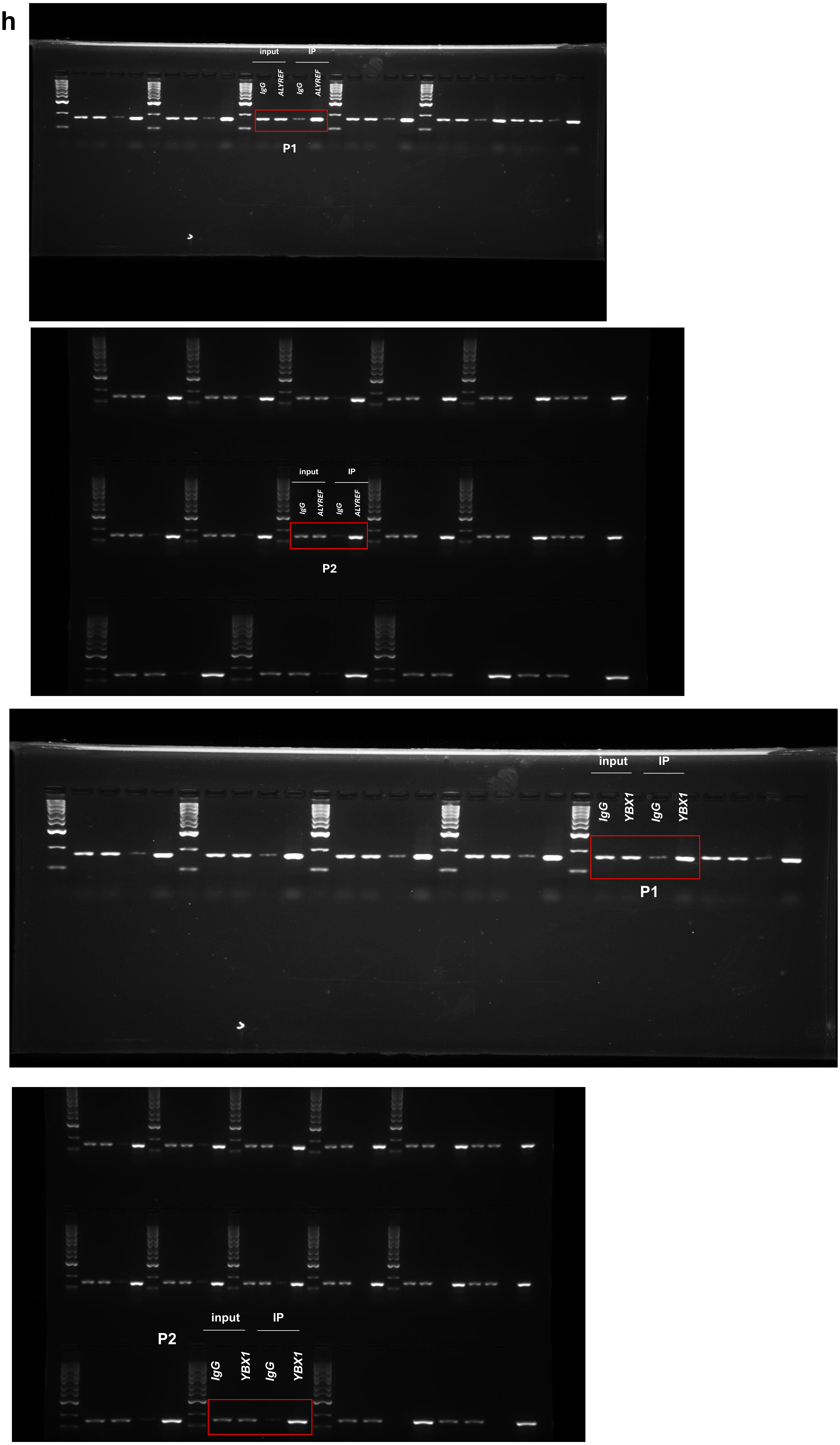

input
IP
IgG
ALYREF
IgG
ALYREF
P1
input
IP
IgG
ALYREF
IgG
ALYREF
P2
input
IP
IgG
YBX1
IgG
YBX1
P1
P2
input
IP
IgG
YBX1
IgG
YBX1

i

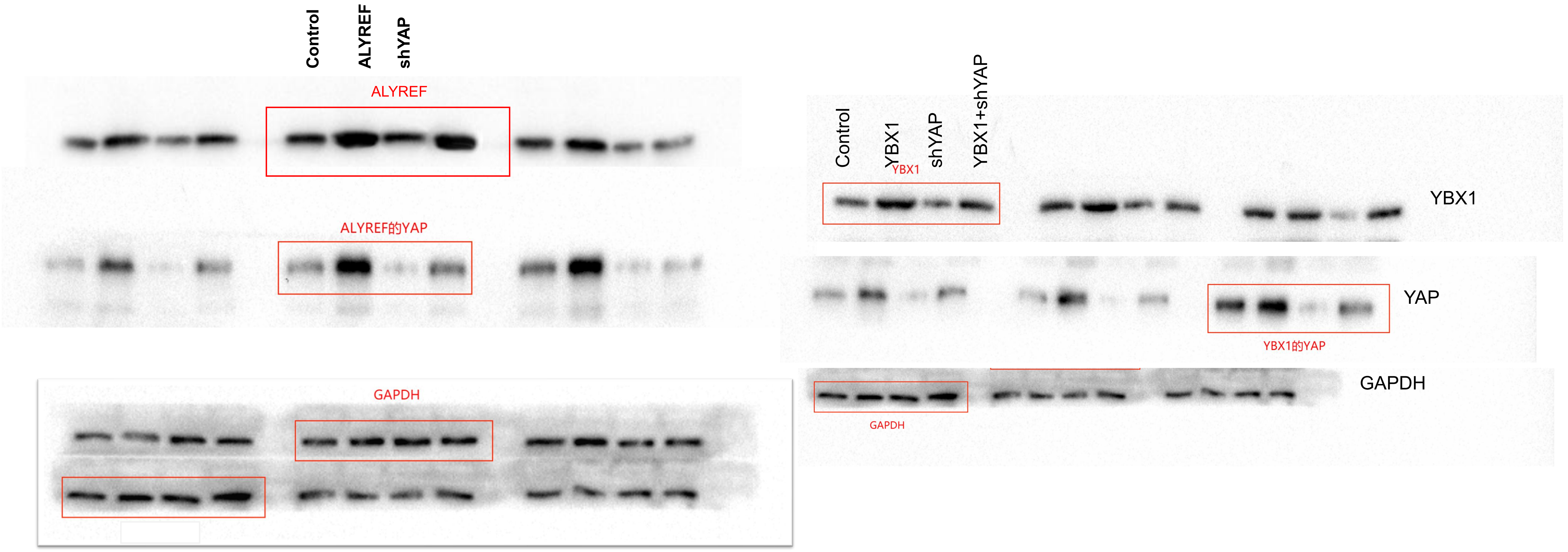
Control
ALYREF
shYAP
ALYREF
ALYREF的YAP
GAPDH
Control
YBX1
shYAP
YBX1+shYAP
YBX1
YBX1
YAP
YBX1的YAP
GAPDH
GAPDH

j

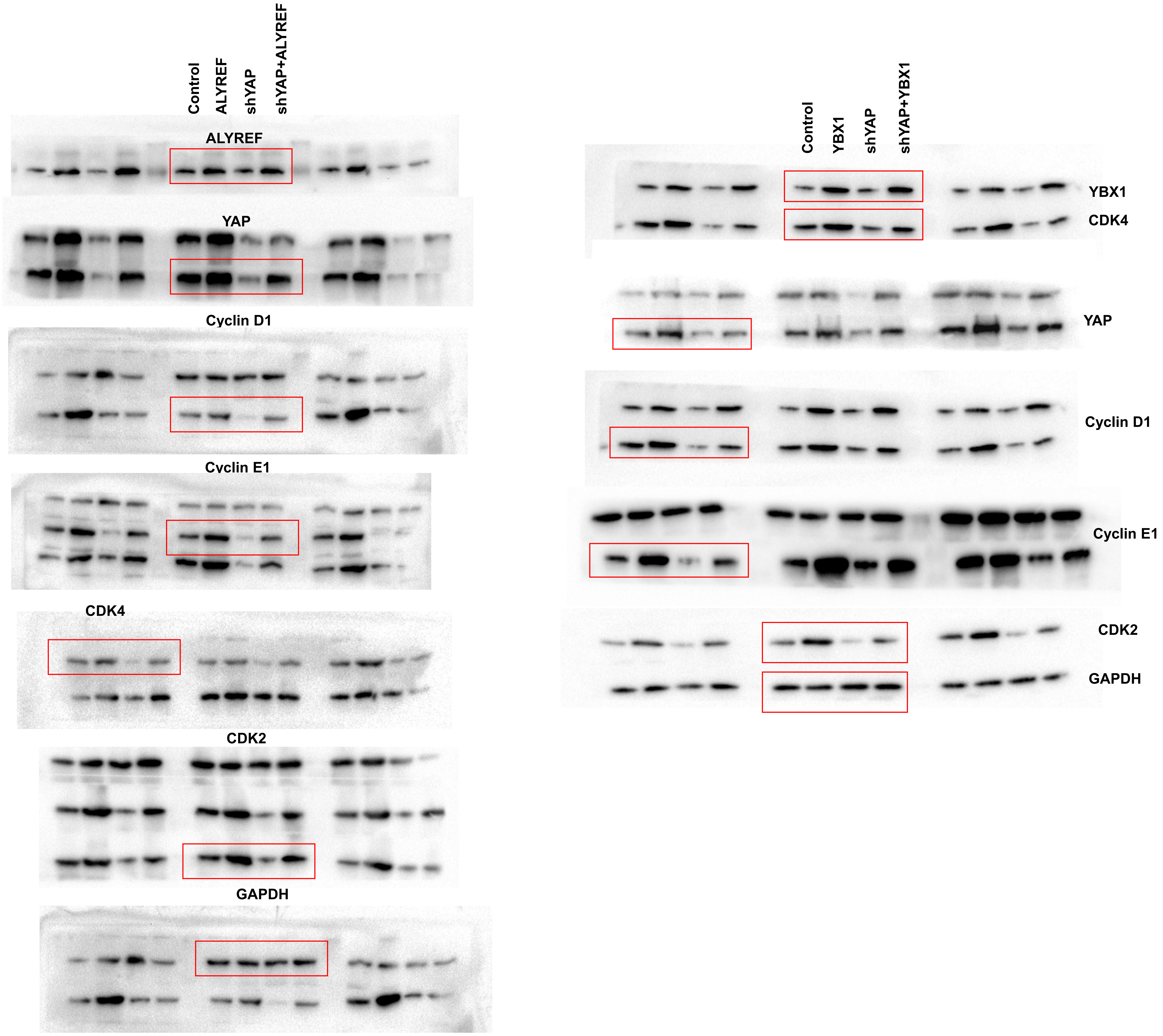
Control
ALYREF
shYAP
shYAP+ALYREF
ALYREF
YAP
Cyclin D1
Cyclin E1
CDK4
CDK2
GAPDH
Control
YBX1
shYAP
shYAP+YBX1
YBX1
CDK4
YAP
Cyclin D1
Cyclin E1
CDK2
GAPDH

k

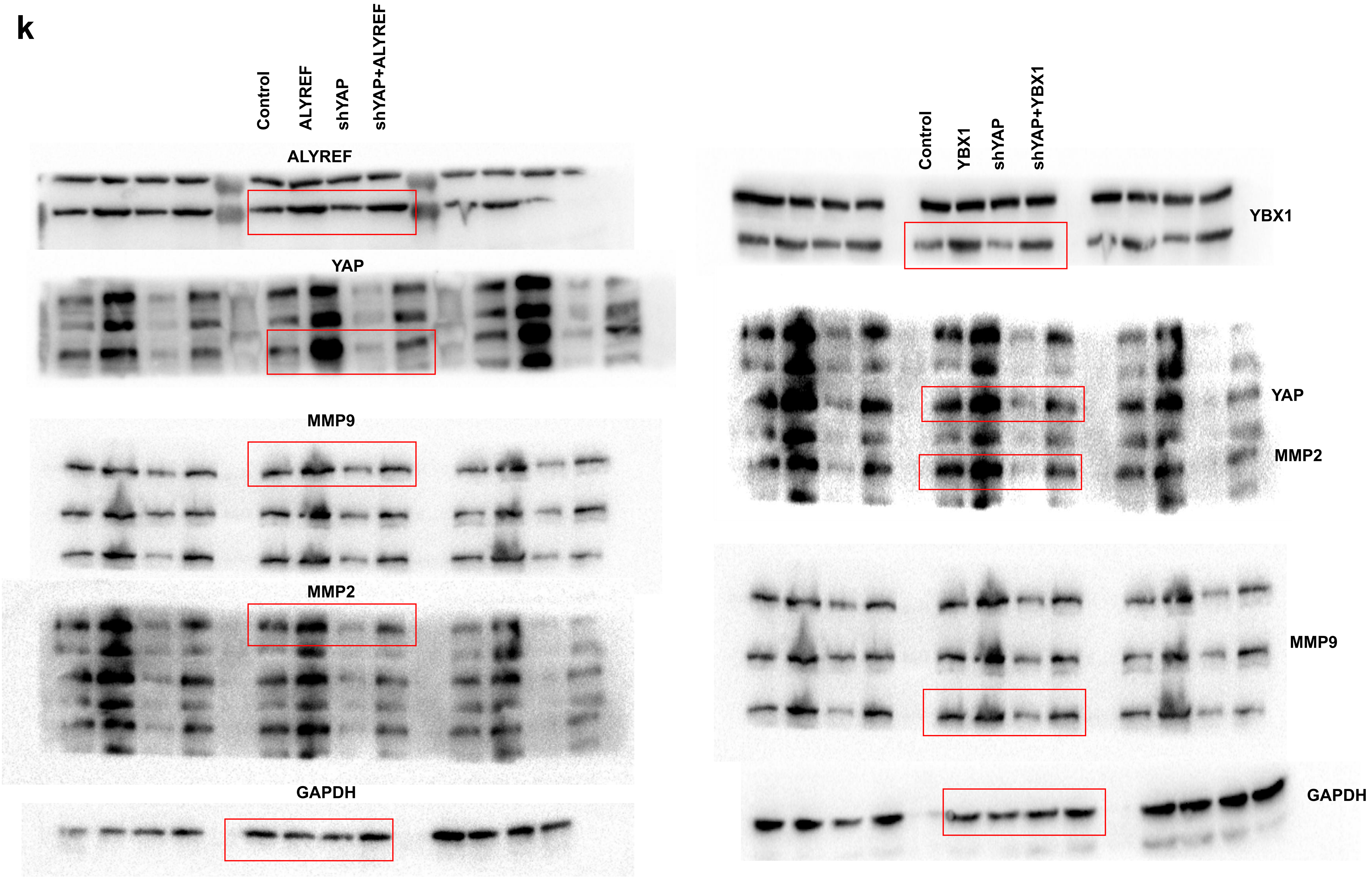
Control
ALYREF
shYAP
shYAP+ALYREF
ALYREF
YAP
MMP9
MMP2
GAPDH
Control
YBX1
shYAP
shYAP+YBX1
YBX1
YAP
MMP2
MMP9
GAPDH

l

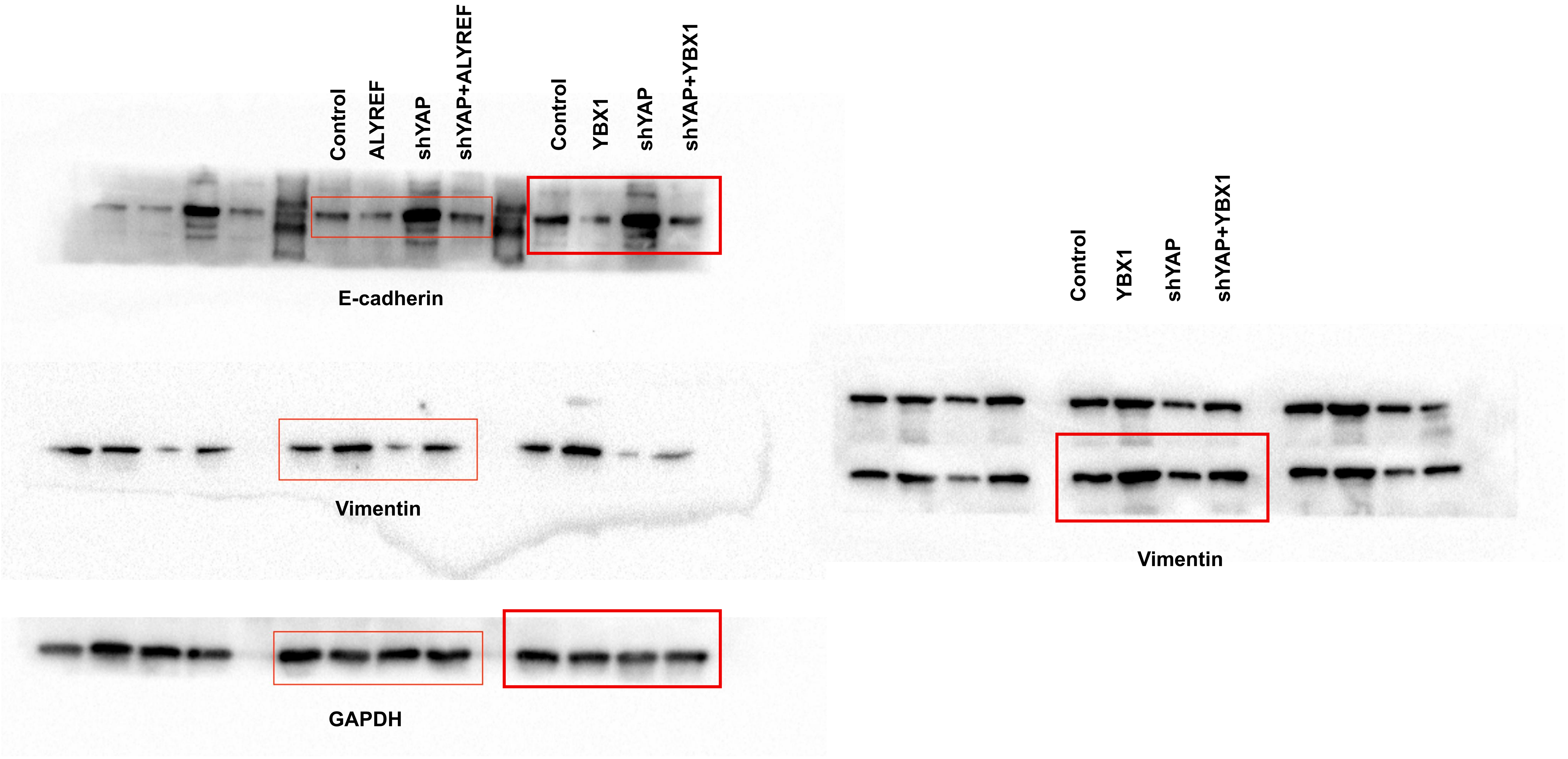
Control
ALYREF
shYAP
shYAP+ALYREF
Control
YBX1
shYAP
shYAP+YBX1
E-cadherin
Vimentin
GAPDH
Control
YBX1
shYAP
shYAP+YBX1
Vimentin

Fig 4

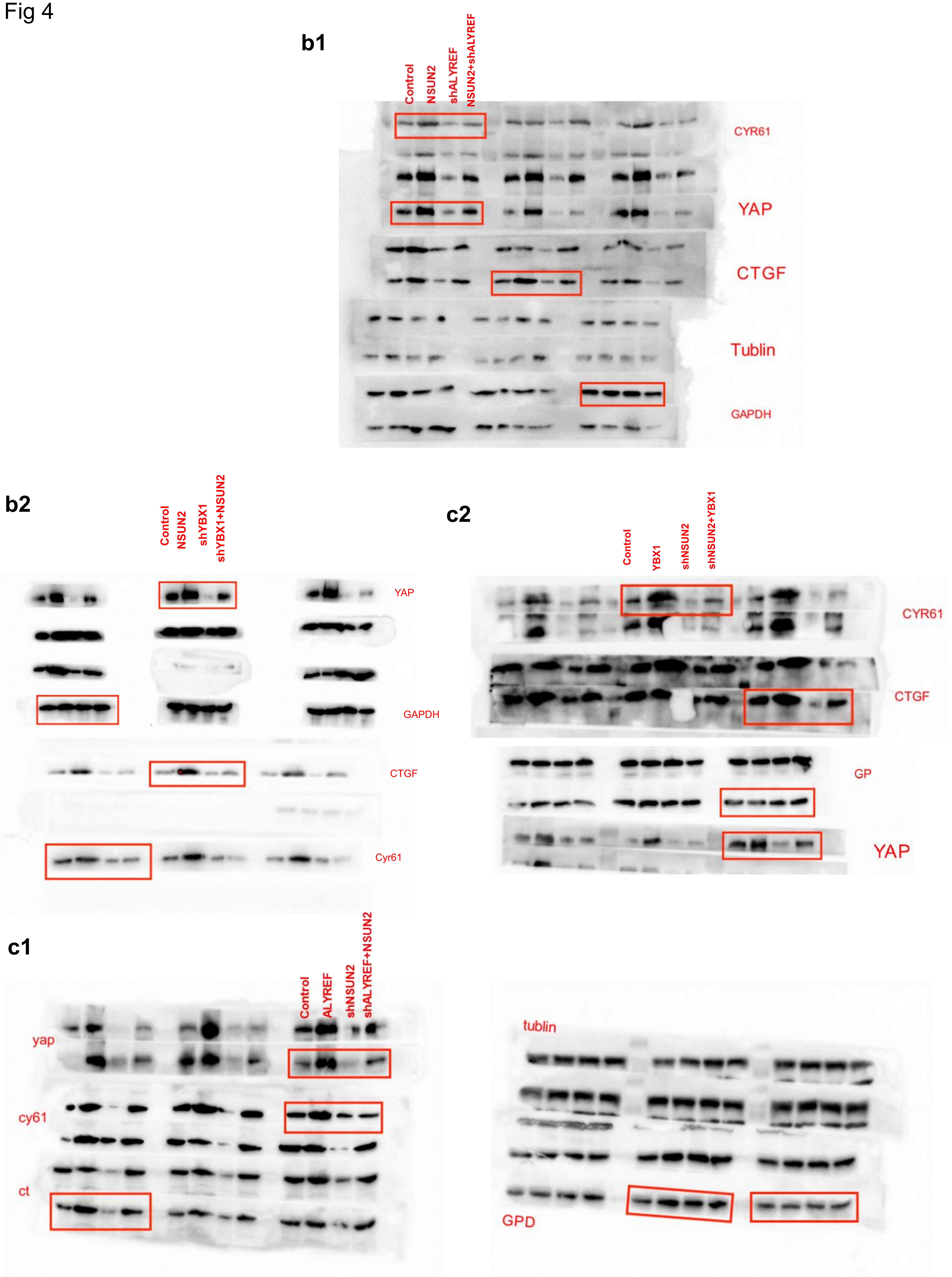
b1
Control
NSUN2
shALYREF
NSUN2+shALYREF
CYR61
YAP
CTGF
Tublin
GAPDH
b2
Control
NSUN2
shYBX1
shYBX1+NSUN2
YAP
GAPDH
CTGF
Cyr61
c2
Control
YBX1
shNSUN2
shNSUN2+YBX1
CYR61
CTGF
GP
YAP
c1
Control
ALYREF
shNSUN2
shALYREF+NSUN2
yap
cy61
ct
tublin
GPD

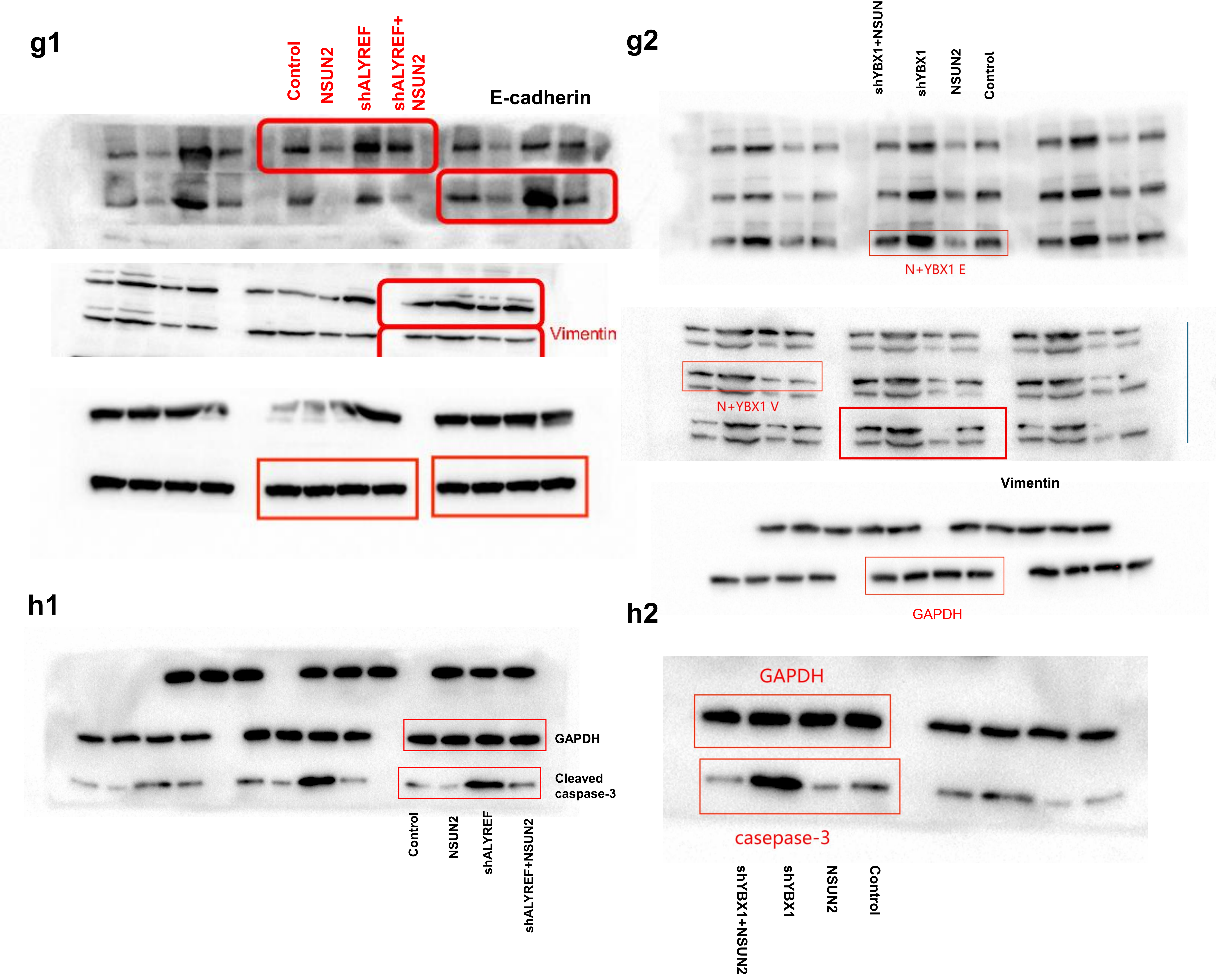
g1
Control
NSUN2
shALYREF
shALYREF+ NSUN2
E-cadherin
Vimentin
g2
shYBX1+NSUN
shYBX1
NSUN2
Control
N+YBX1 E
N+YBX1 V
Vimentin
GAPDH
h1
GAPDH
Cleaved caspase-3
Control
NSUN2
shALYREF
shALYREF+NSUN2
h2
GAPDH
casepase-3
shYBX1+NSUN2
shYBX1
NSUN2
Control

k

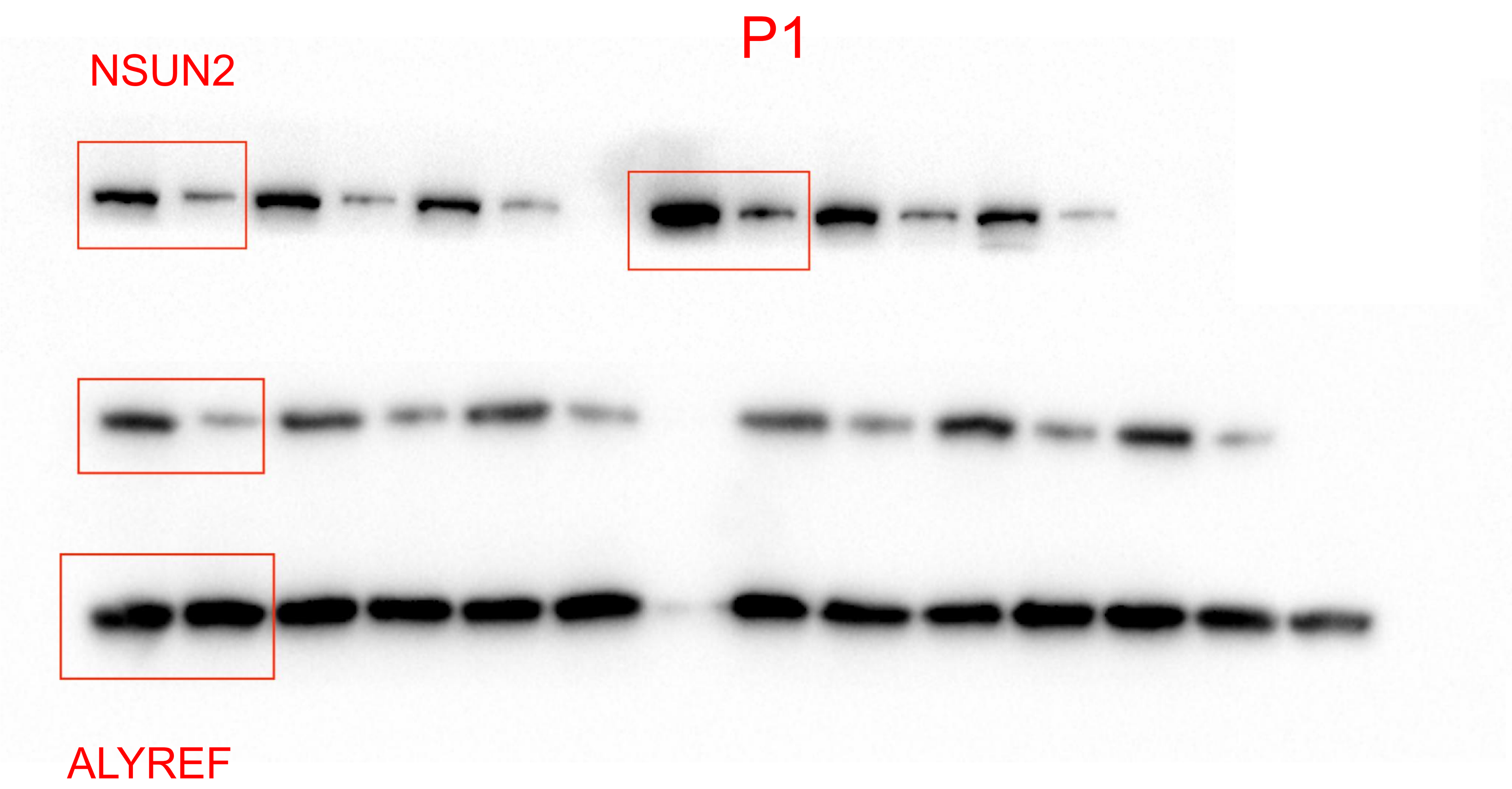
P1
NSUN2
ALYREF

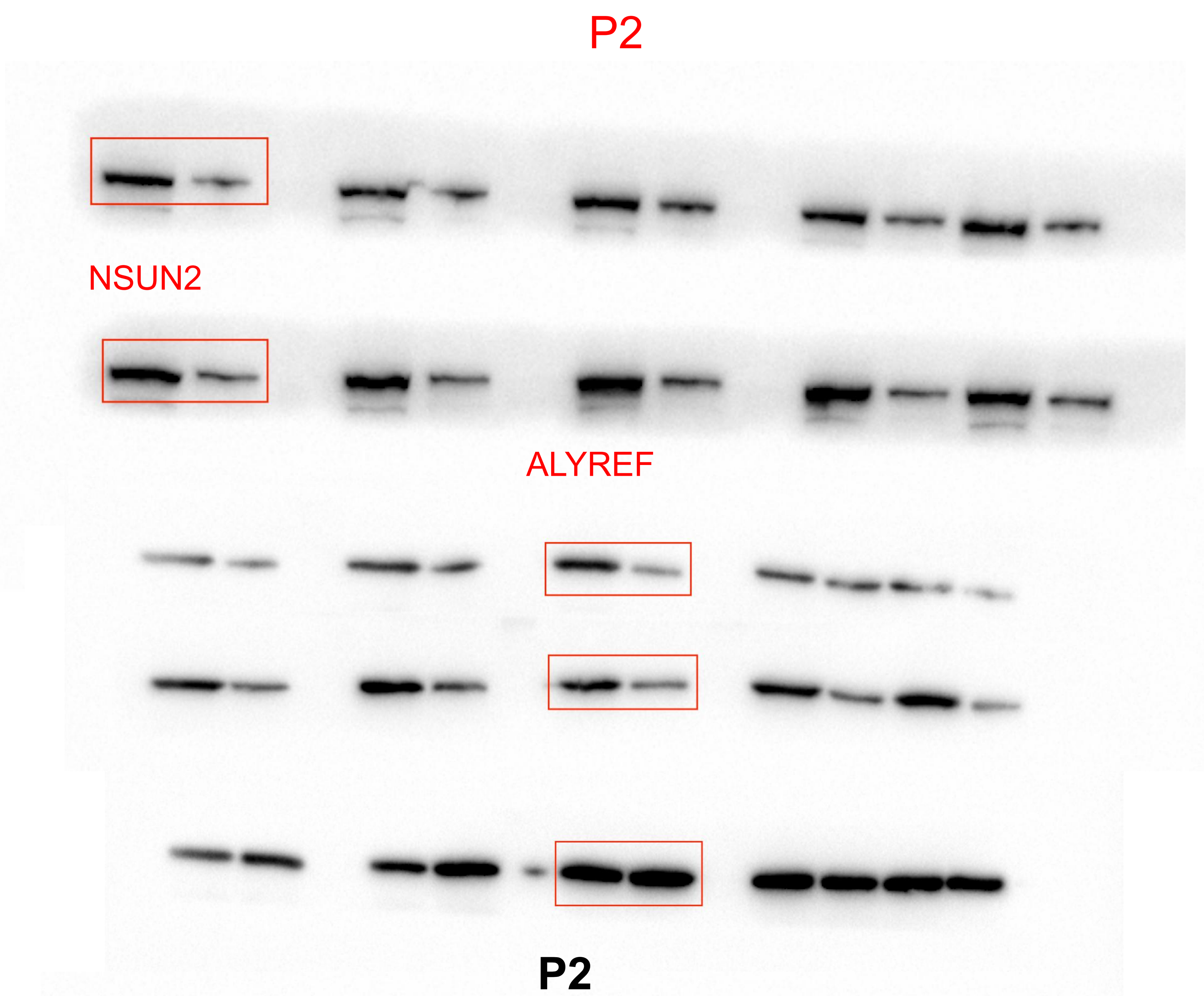
P2
NSUN2
ALYREF

l

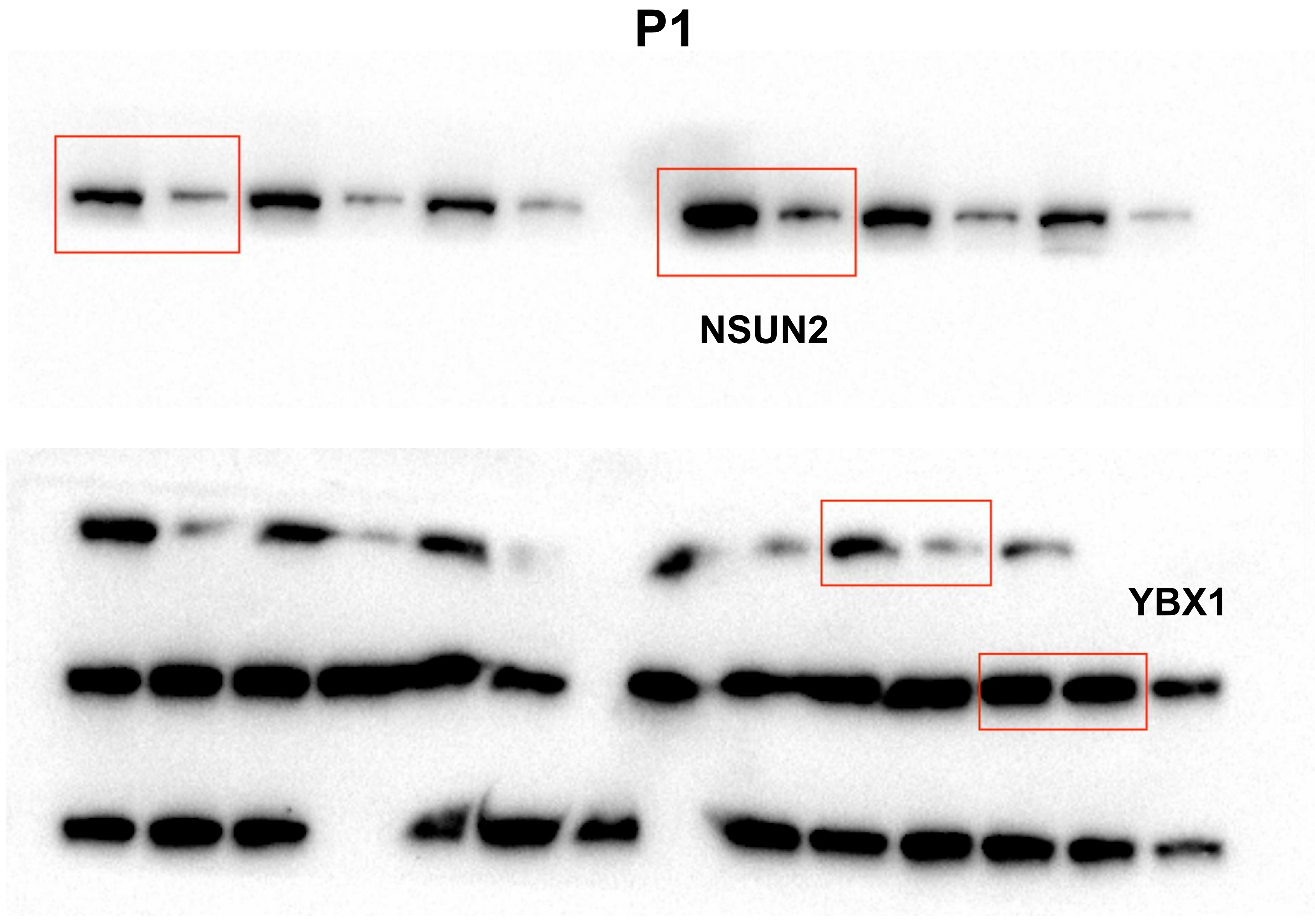
P1
NSUN2
YBX1

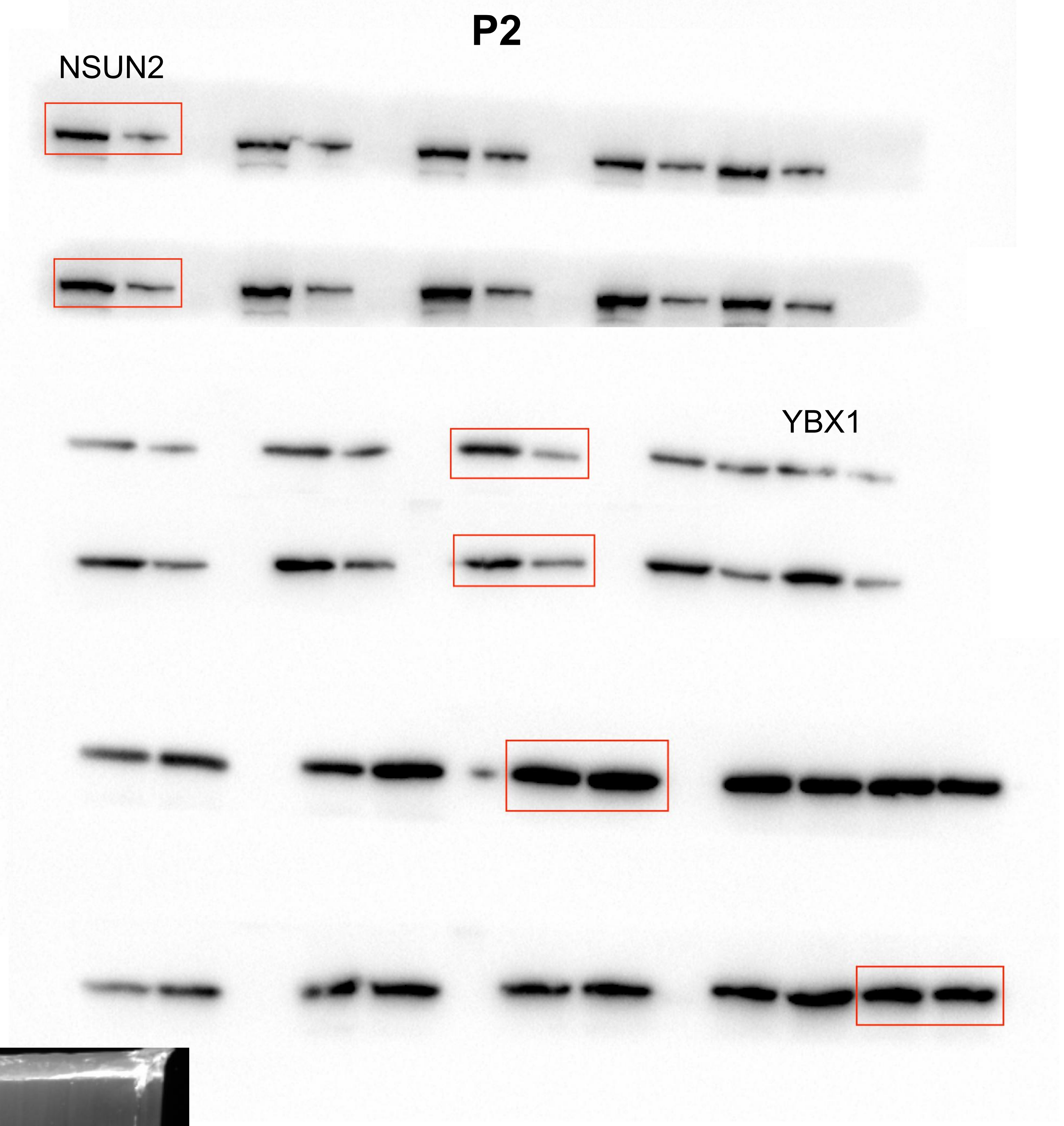
P2
NSUN2
YBX1

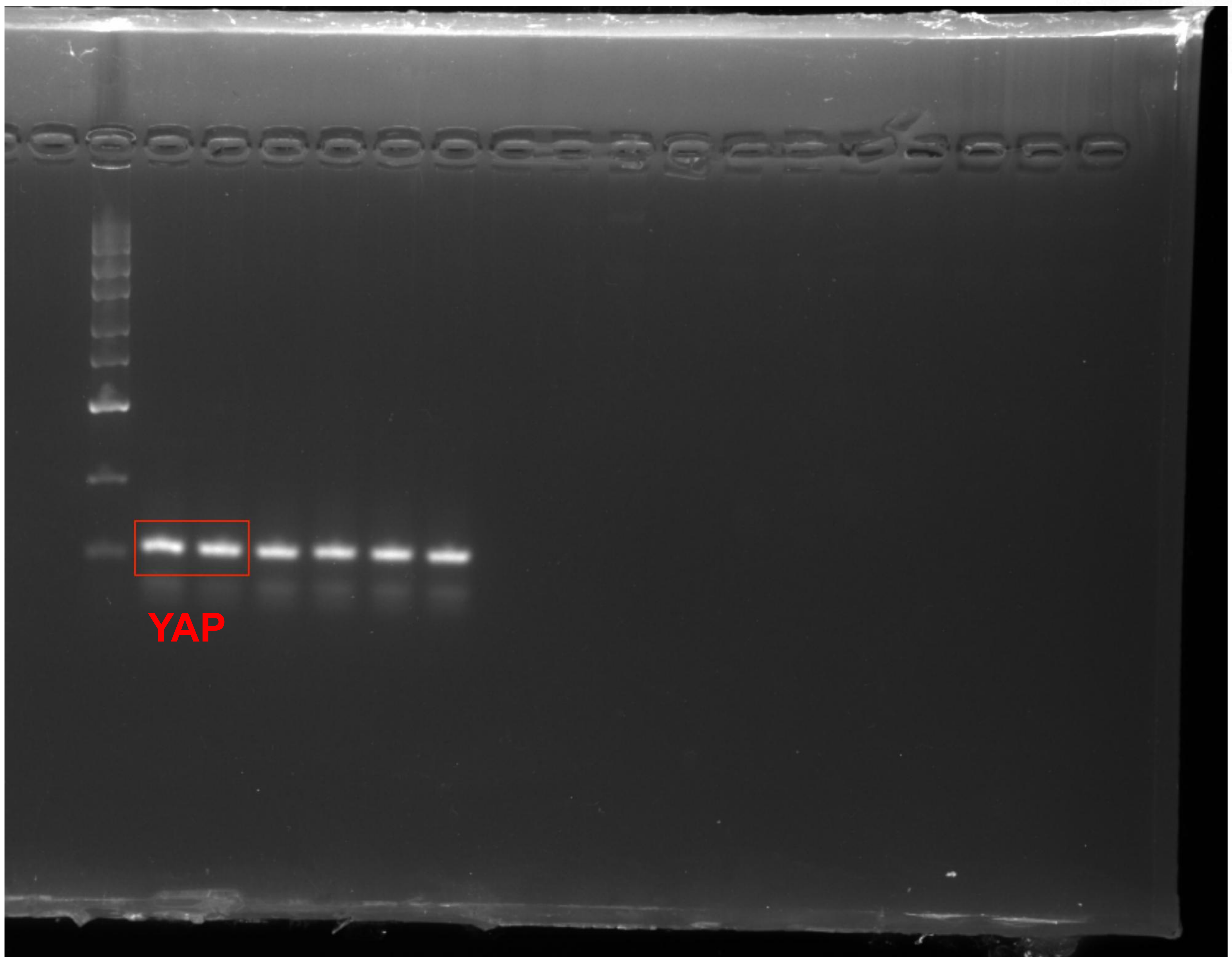
YAP

Fig 5

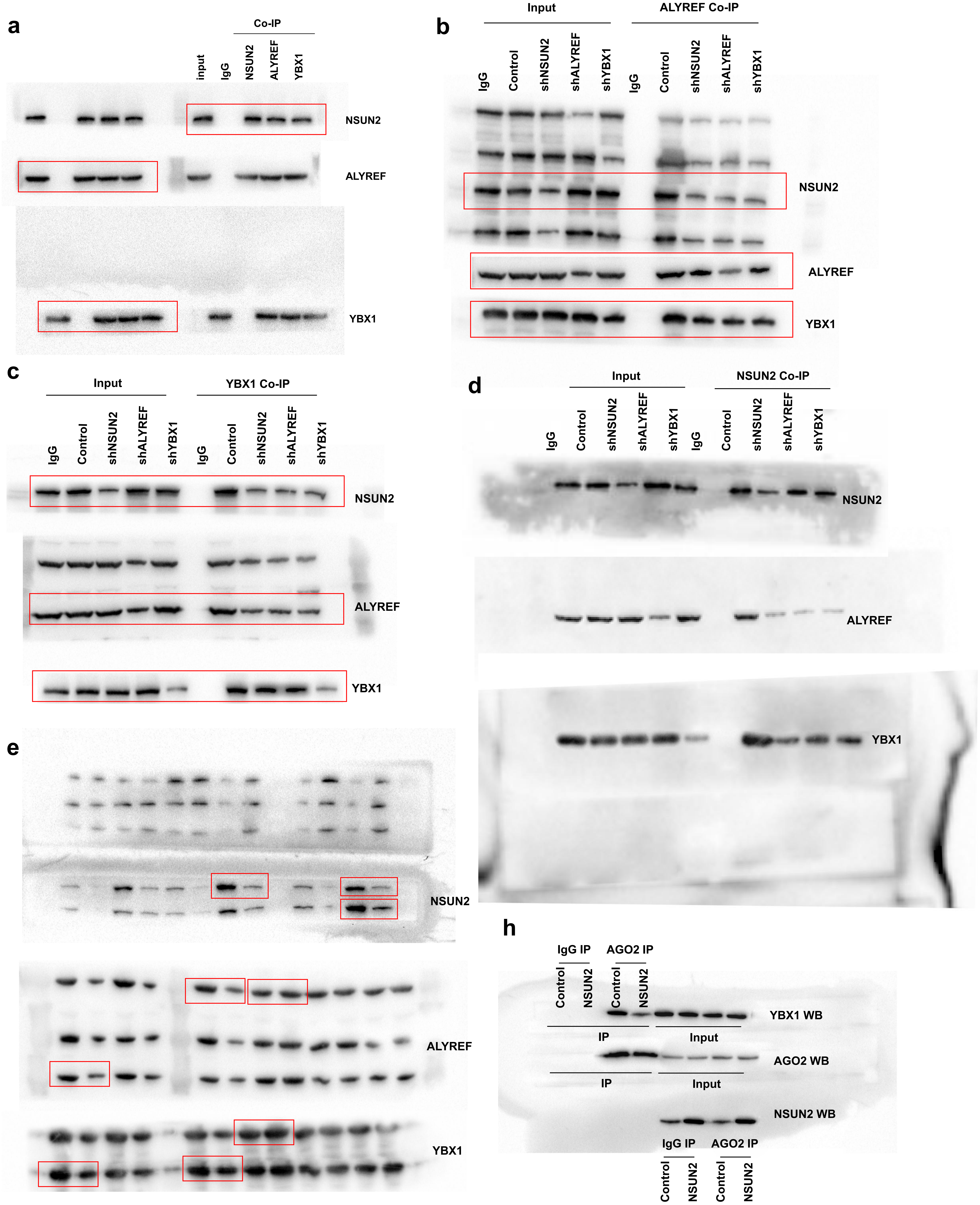
a
Co-IP
input
IgG
NSUN2
ALYREF
YBX1
NSUN2
ALYREF
YBX1
b
Input
ALYREF Co-IP
IgG
Control
shNSUN2
shALYREF
shYBX1
IgG
Control
shNSUN2
shALYREF
shYBX1
NSUN2
ALYREF
YBX1
c
Input
YBX1 Co-IP
IgG
Control
shNSUN2
shALYREF
shYBX1
IgG
Control
shNSUN2
shALYREF
shYBX1
NSUN2
ALYREF
YBX1
d
Input
NSUN2 Co-IP
IgG
Control
shNSUN2
shALYREF
shYBX1
IgG
Control
shNSUN2
shALYREF
shYBX1
NSUN2
ALYREF
YBX1
e
NSUN2
ALYREF
YBX1
h
IgG IP
AGO2 IP
Control
NSUN2
Control
NSUN2
YBX1 WB
IP
Input
AGO2 WB
IP
Input
NSUN2 WB
IgG IP
AGO2 IP
Control
NSUN2
Control
NSUN2

f

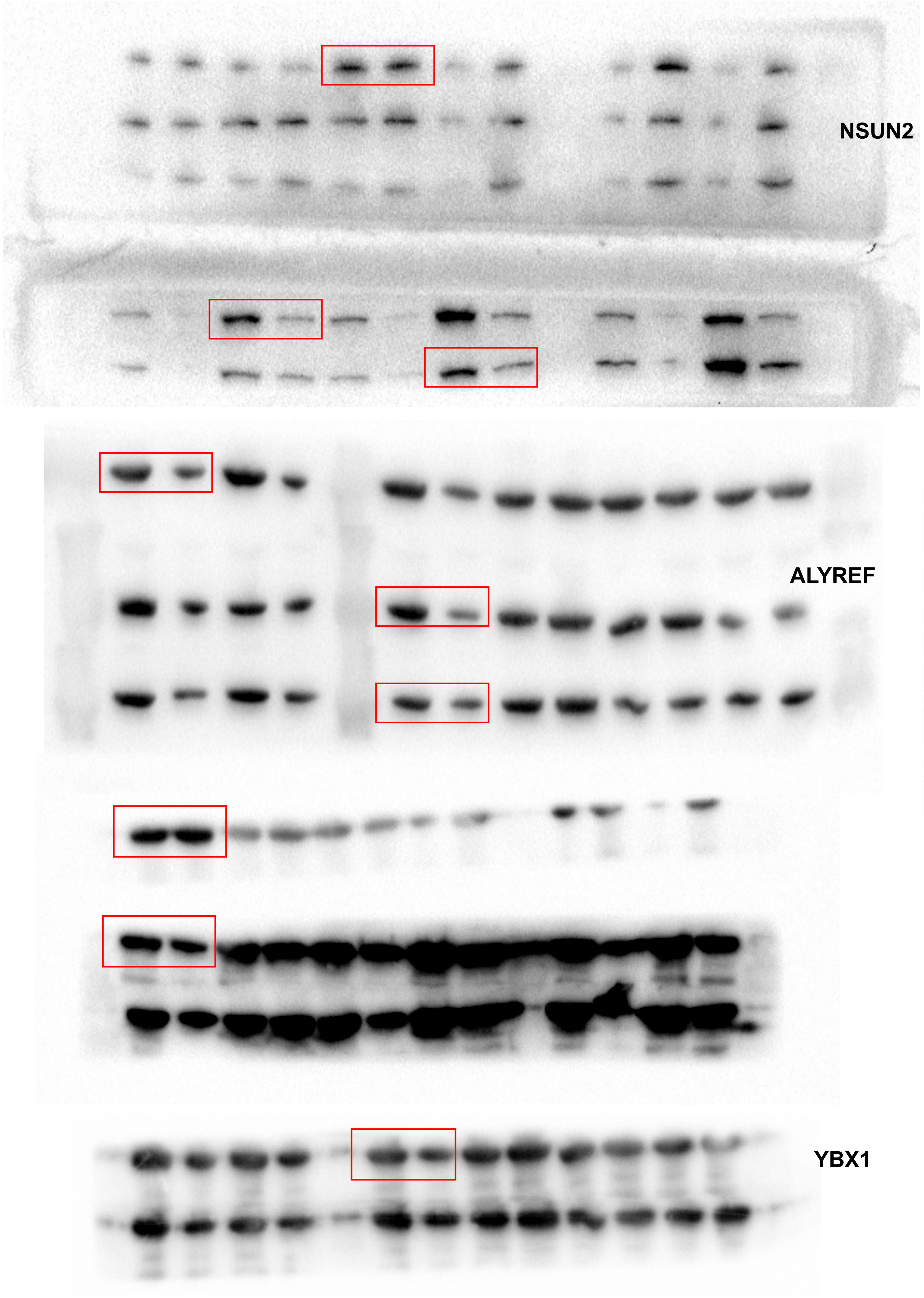
NSUN2
ALYREF
YBX1

g

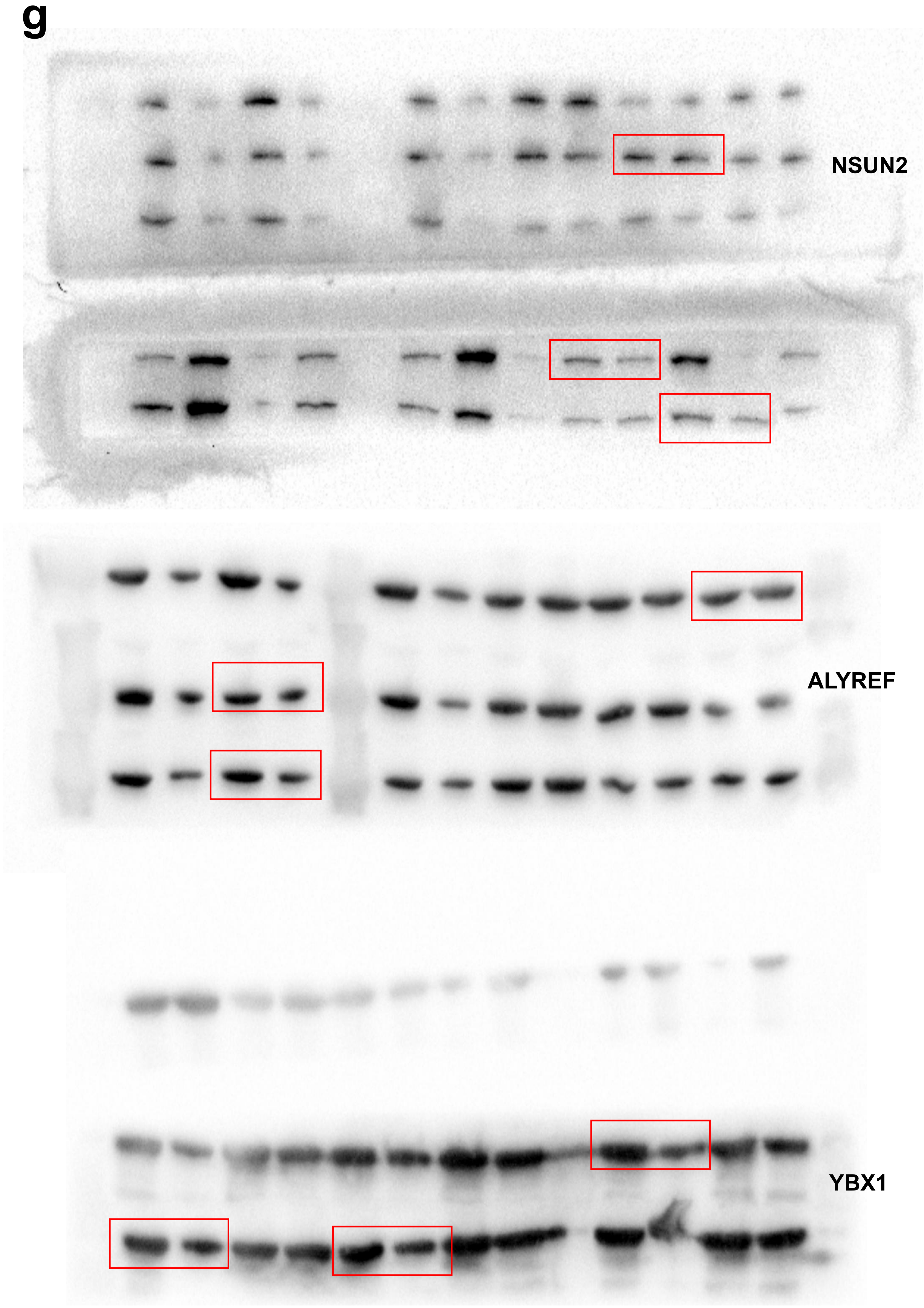
NSUN2
ALYREF
YBX1

l

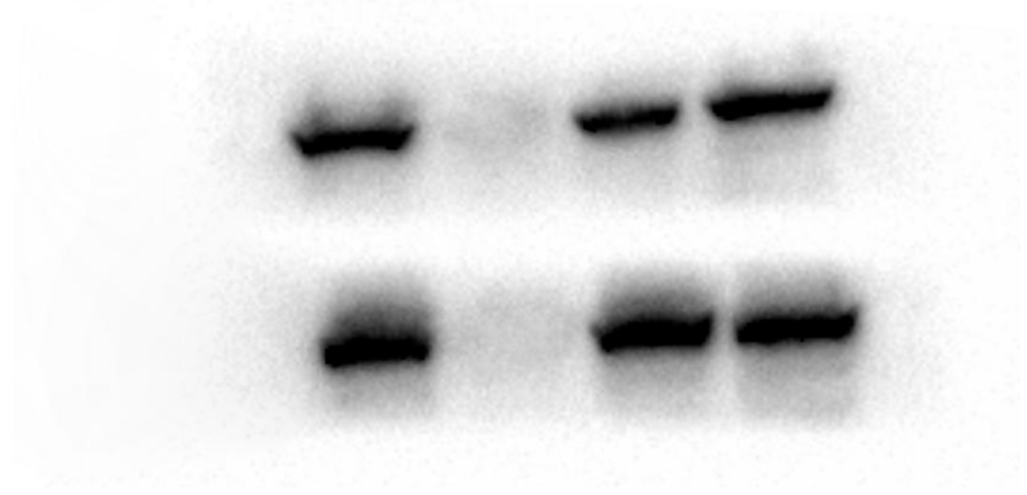

o

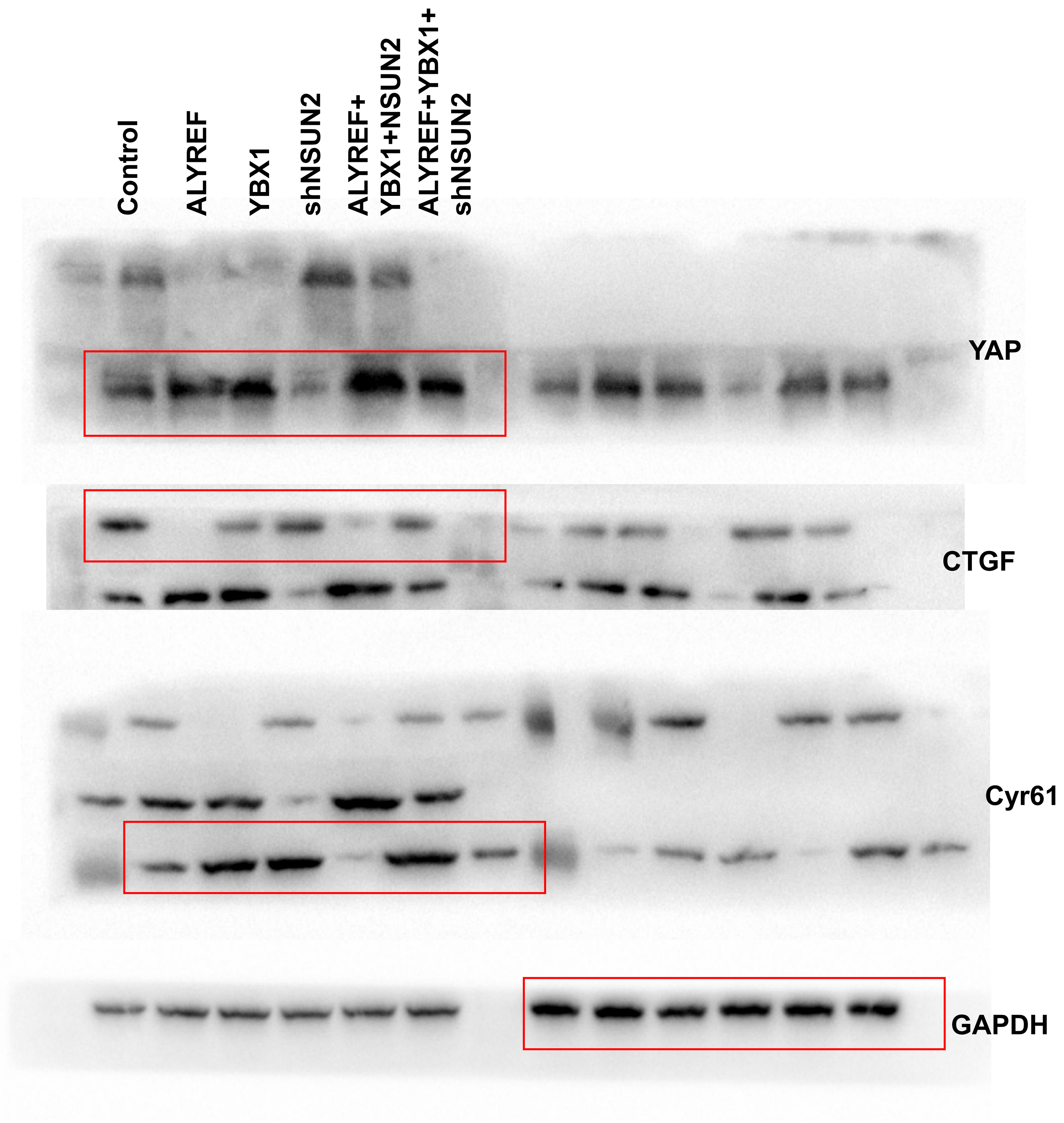
Control
ALYREF
YBX1
shNSUN2
ALYREF+
YBX1+NSUN2
ALYREF+YBX1+
shNSUN2
YAP
CTGF
Cyr61
GAPDH

Fig 6

h

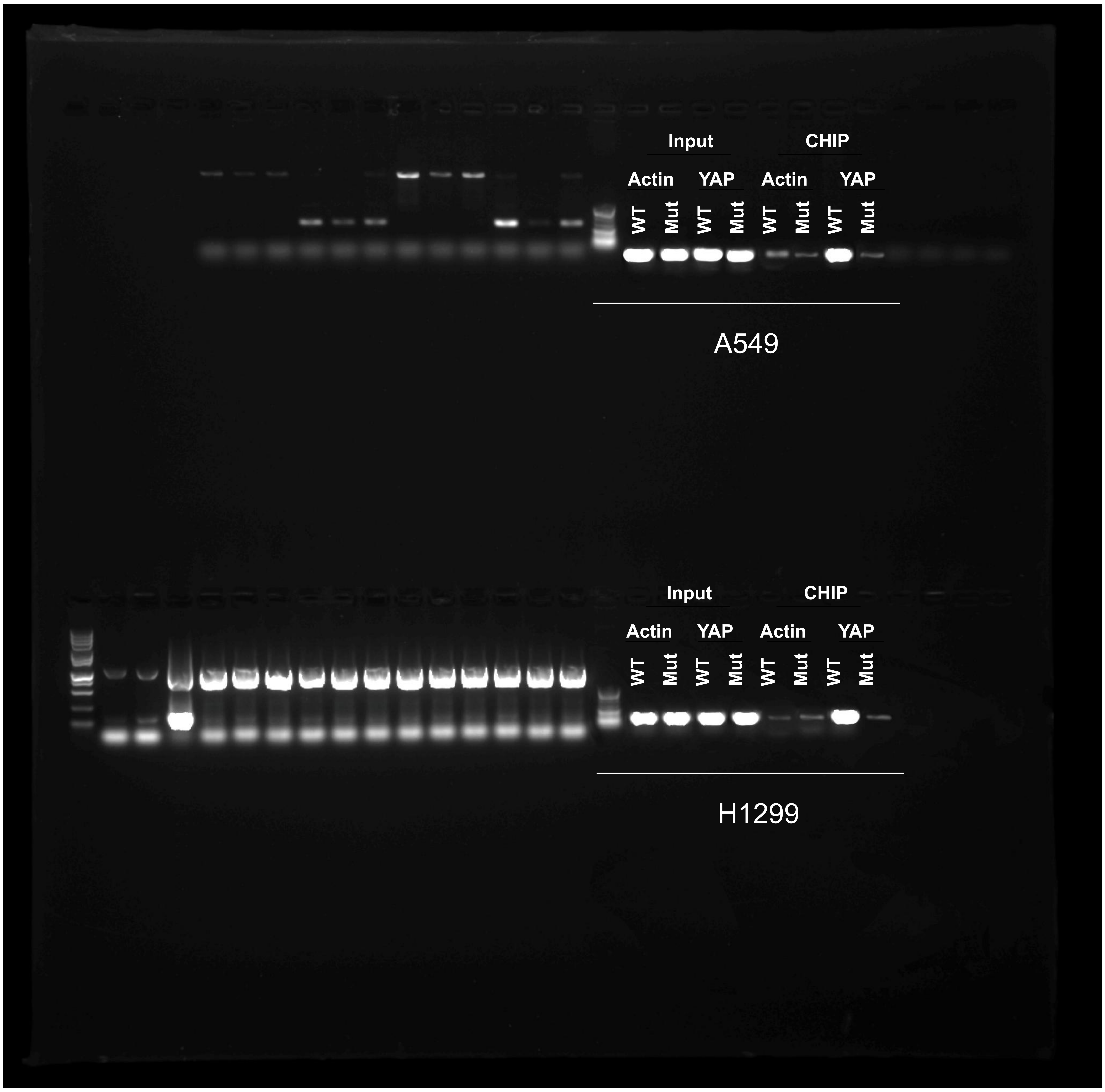
Input
CHIP
Actin
YAP
Actin
YAP
WT
Mut
WT
Mut
WT
Mut
WT
Mut
A549
Input
CHIP
Actin
YAP
Actin
YAP
WT
Mut
WT
Mut
WT
Mut
WT
Mut
H1299

I

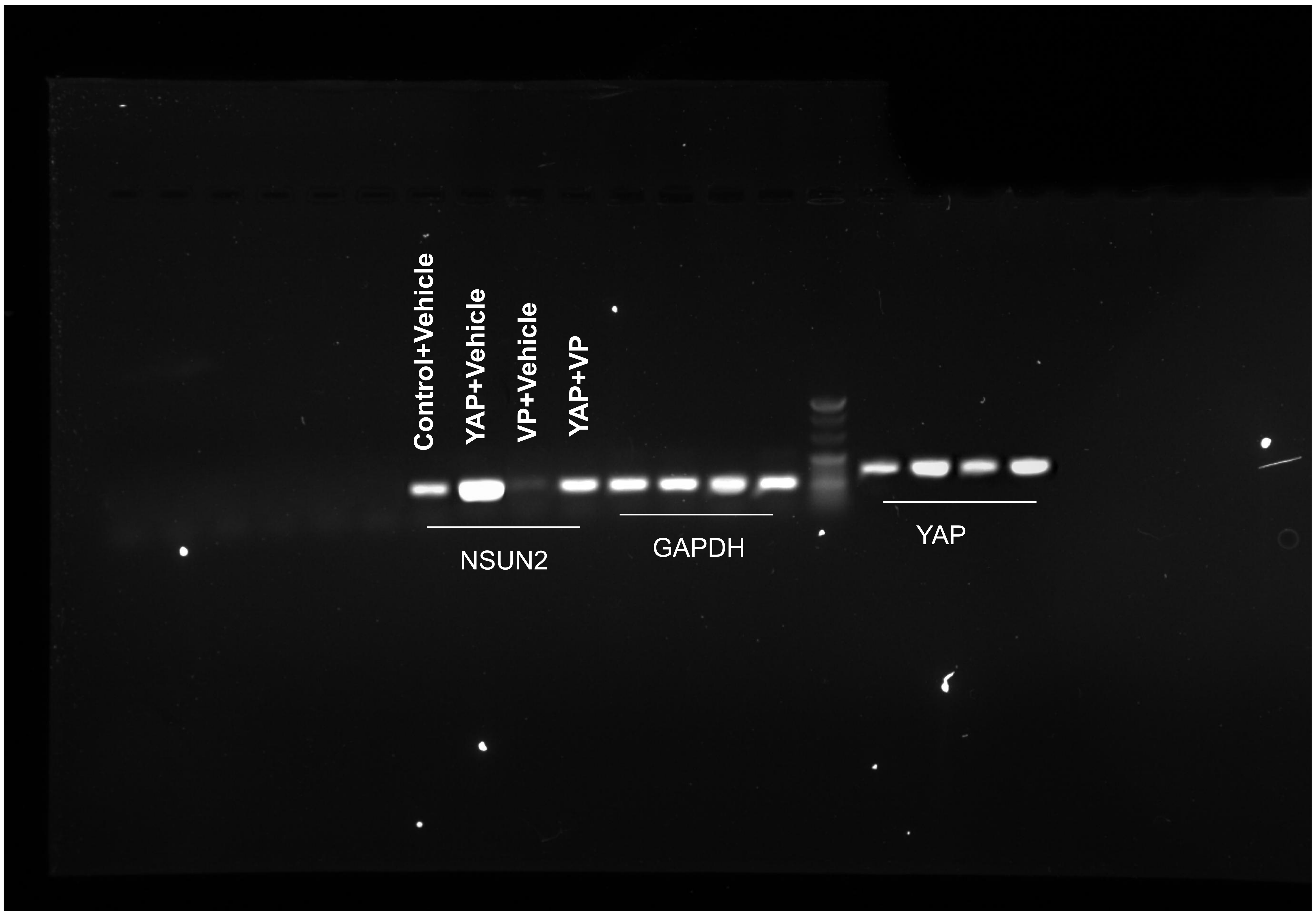
Control+Vehicle
YAP+Vehicle
VP+Vehicle
YAP+VP
NSUN2
GAPDH
YAP

Fig 7

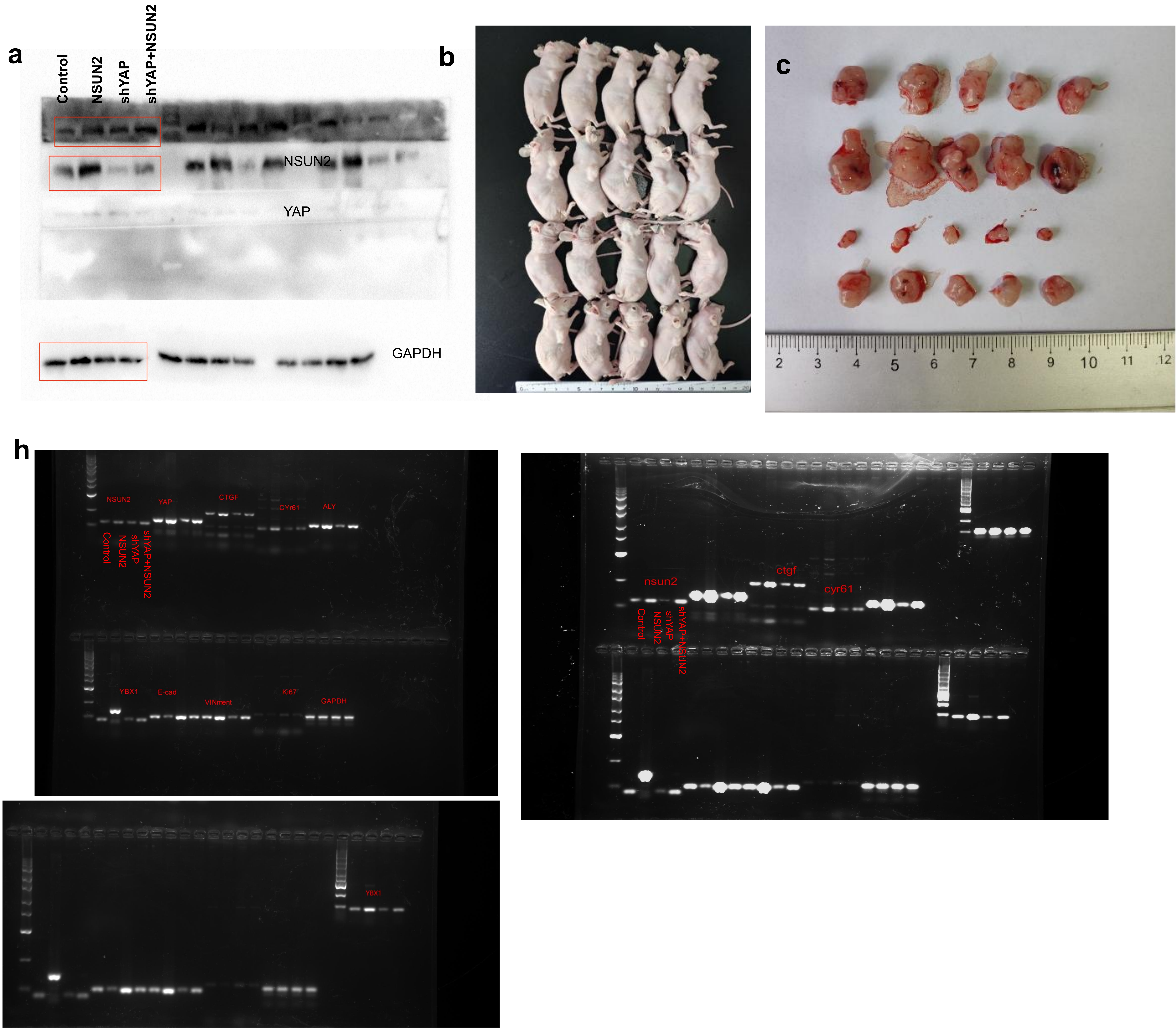
a
Control
NSUN2
shYAP
shYAP+NSUN2
NSUN2
YAP
GAPDH
b
c
2
3
4
5
6
7
8
9
10
11
12
h
NSUN2
YAP
CTGF
CYr61
ALY
Control
NSUN2
shYAP
shYAP+NSUN2
YBX1
E-cad
VINment
Ki67
GAPDH
nsun2
ctgf
cyr61
Control
NSUN2
shYAP
shYAP+NSUN2
YBX1

Fig 8

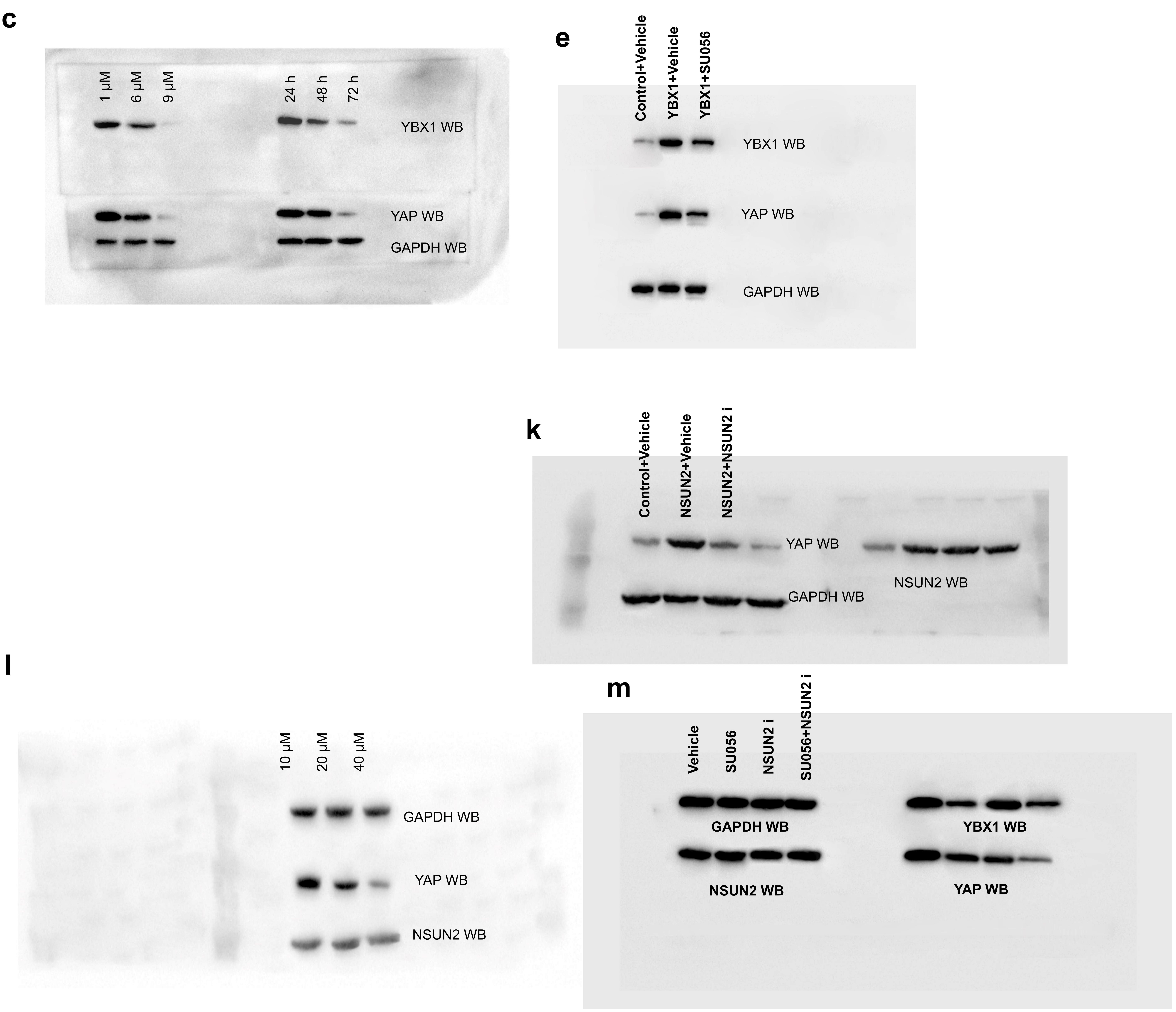
c
1 μM
6 μM
9 μM
24 h
48 h
72 h
YBX1 WB
YAP WB
GAPDH WB
e
Control+Vehicle
YBX1+Vehicle
YBX1+SU056
YBX1 WB
YAP WB
GAPDH WB
k
Control+Vehicle
NSUN2+Vehicle
NSUN2+NSUN2 i
YAP WB
GAPDH WB
NSUN2 WB
l
10 μM
20 μM
40 μM
GAPDH WB
YAP WB
NSUN2 WB
m
Vehicle
SU056
NSUN2 i
SU056+NSUN2 i
GAPDH WB
YBX1 WB
NSUN2 WB
YAP WB

Fig S1d

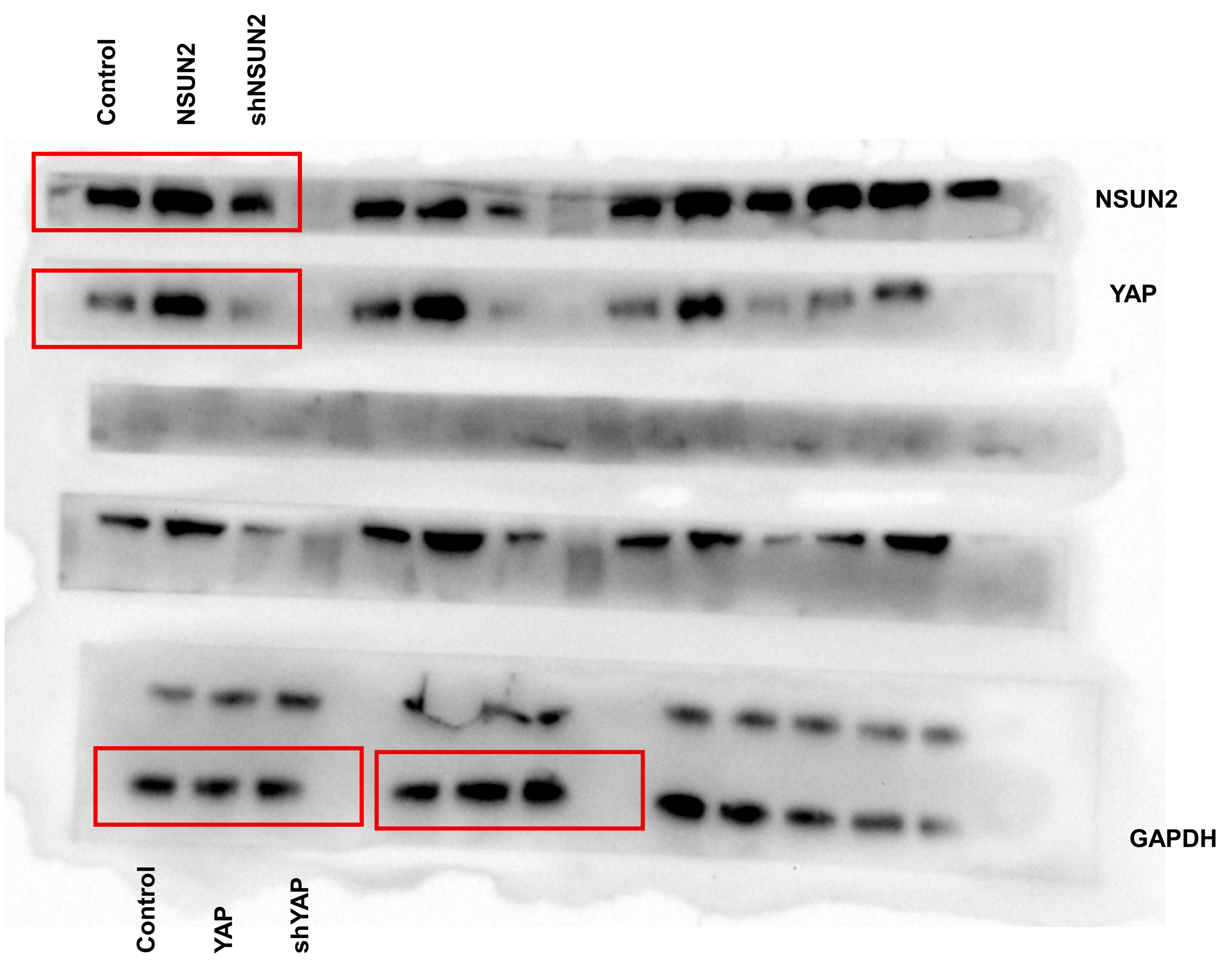

Fig S2d

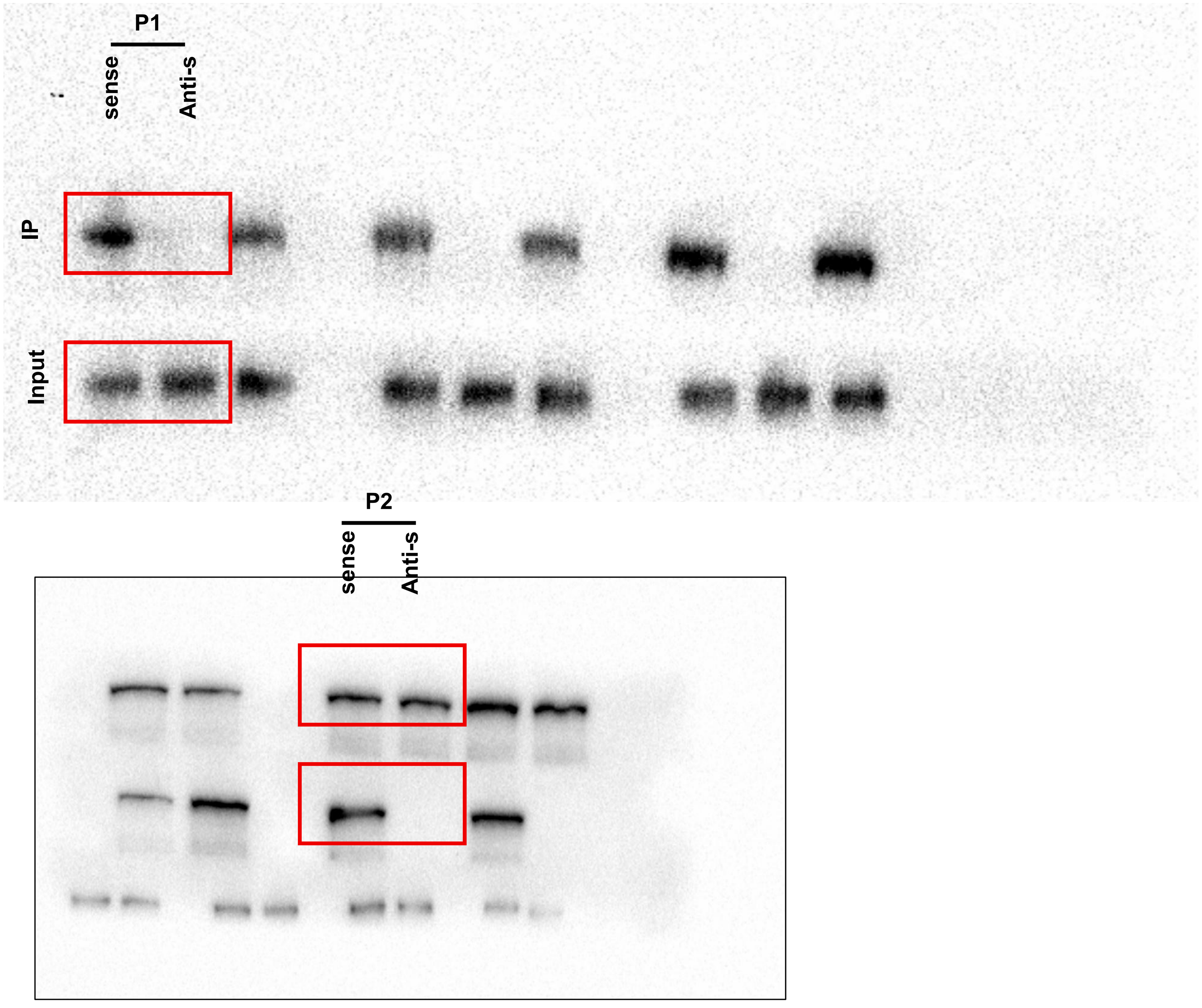

Fig S2e

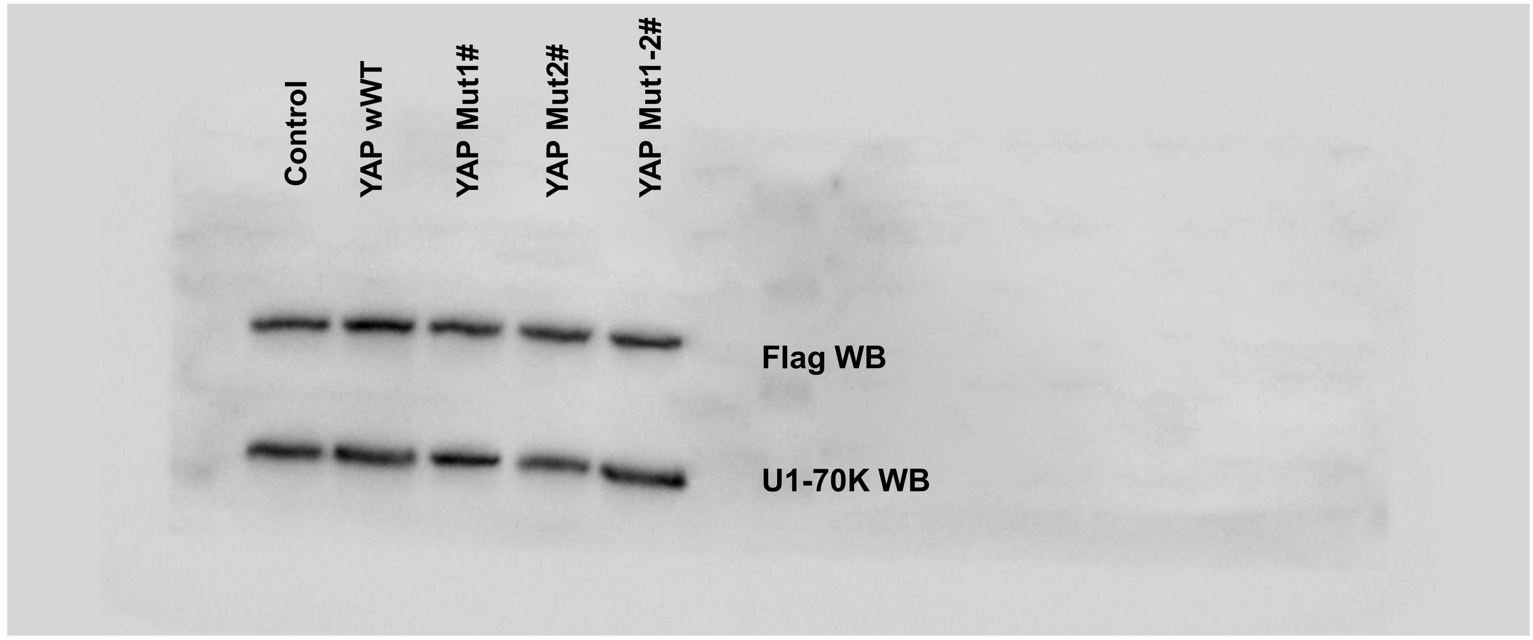

Fig S2j

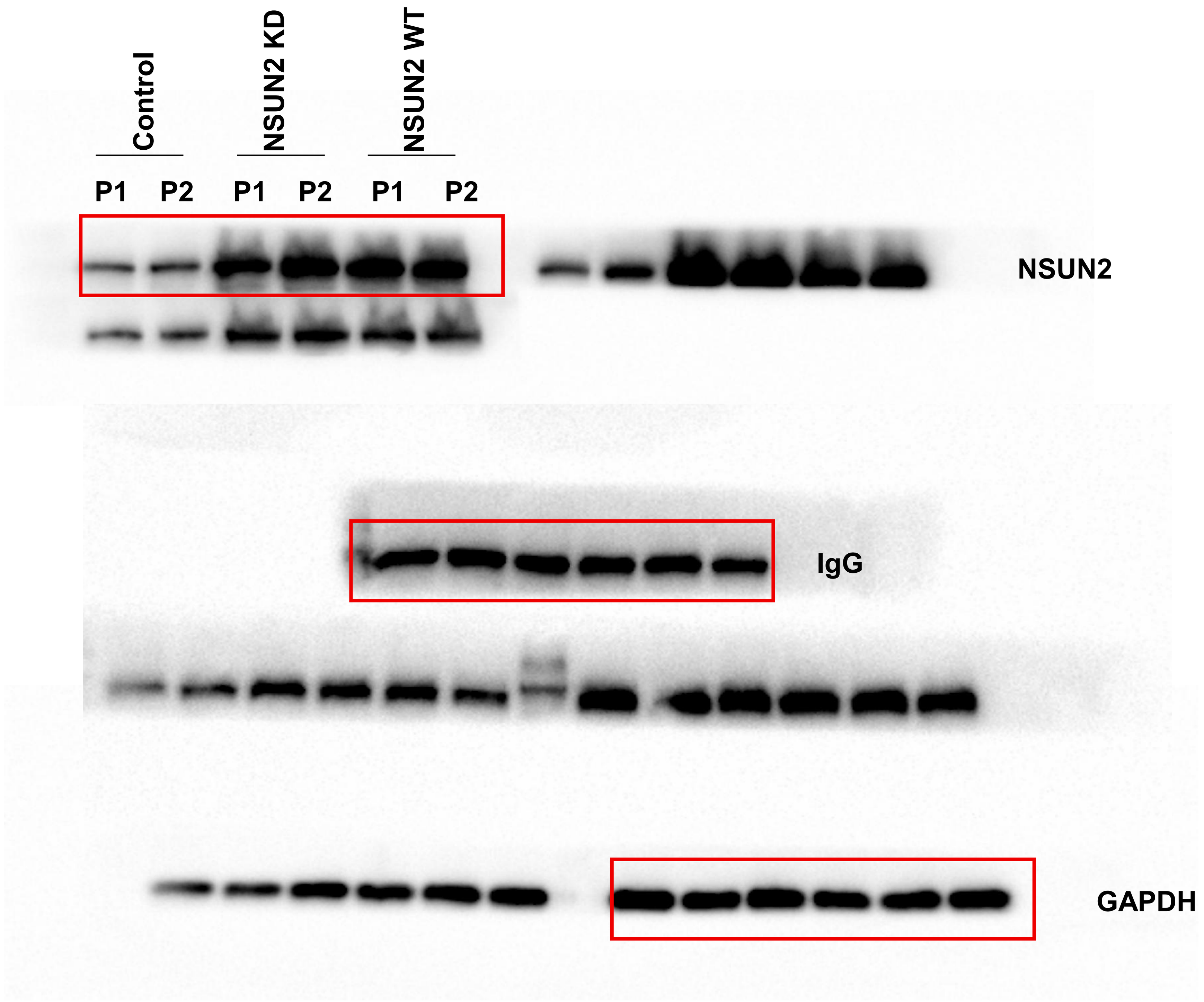

Fig S2k

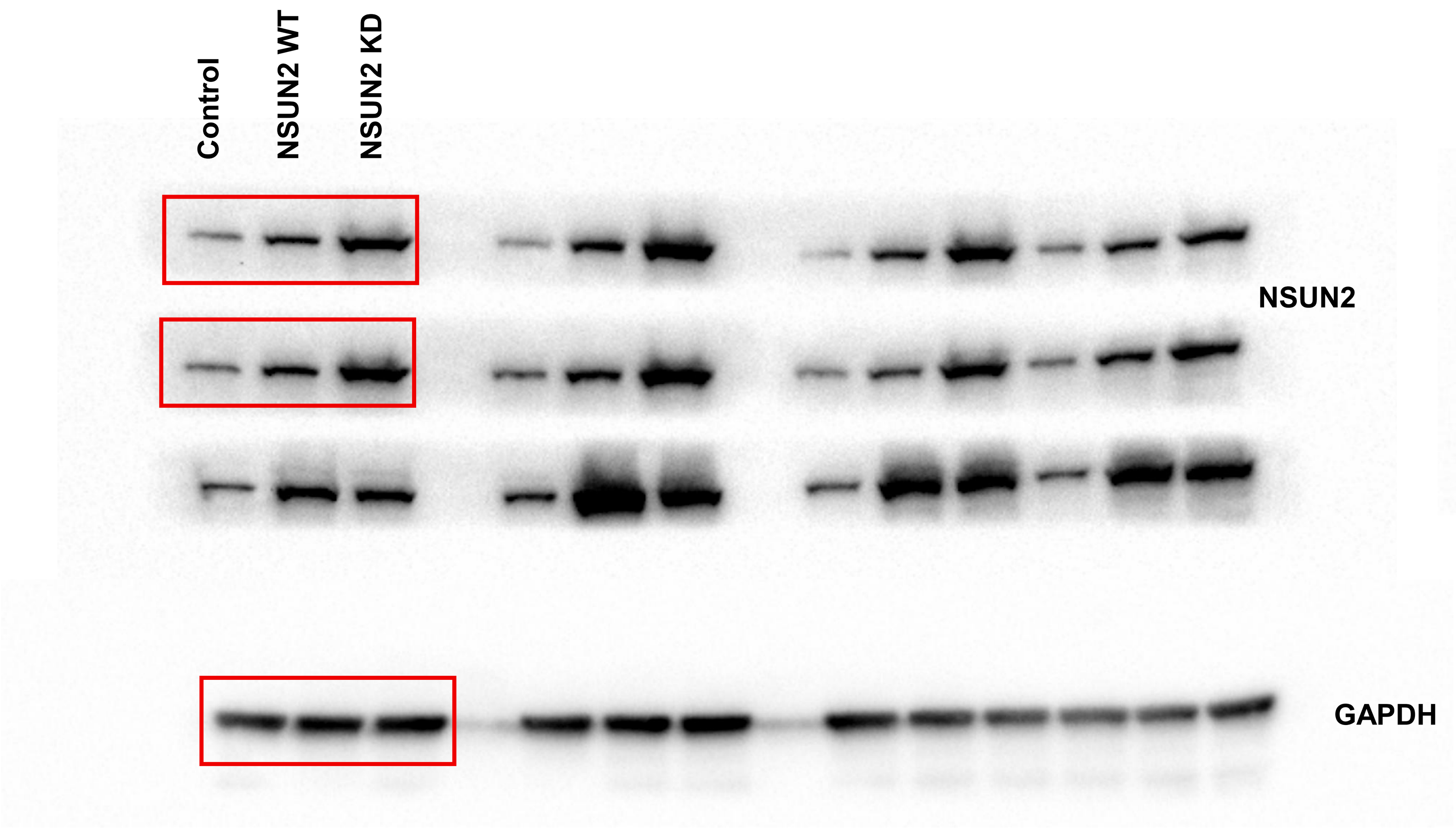

Fig S2q

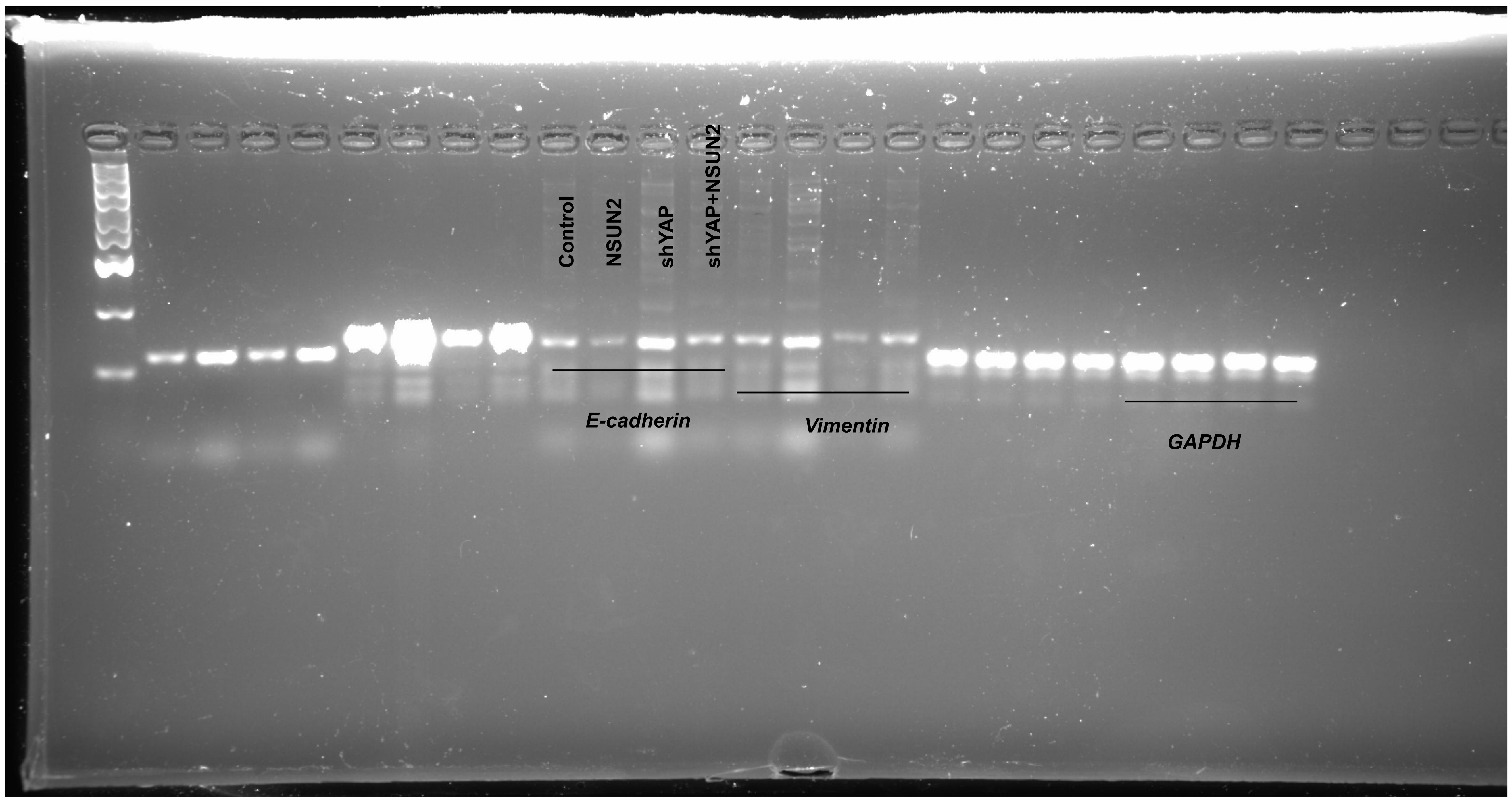

Fig S3a

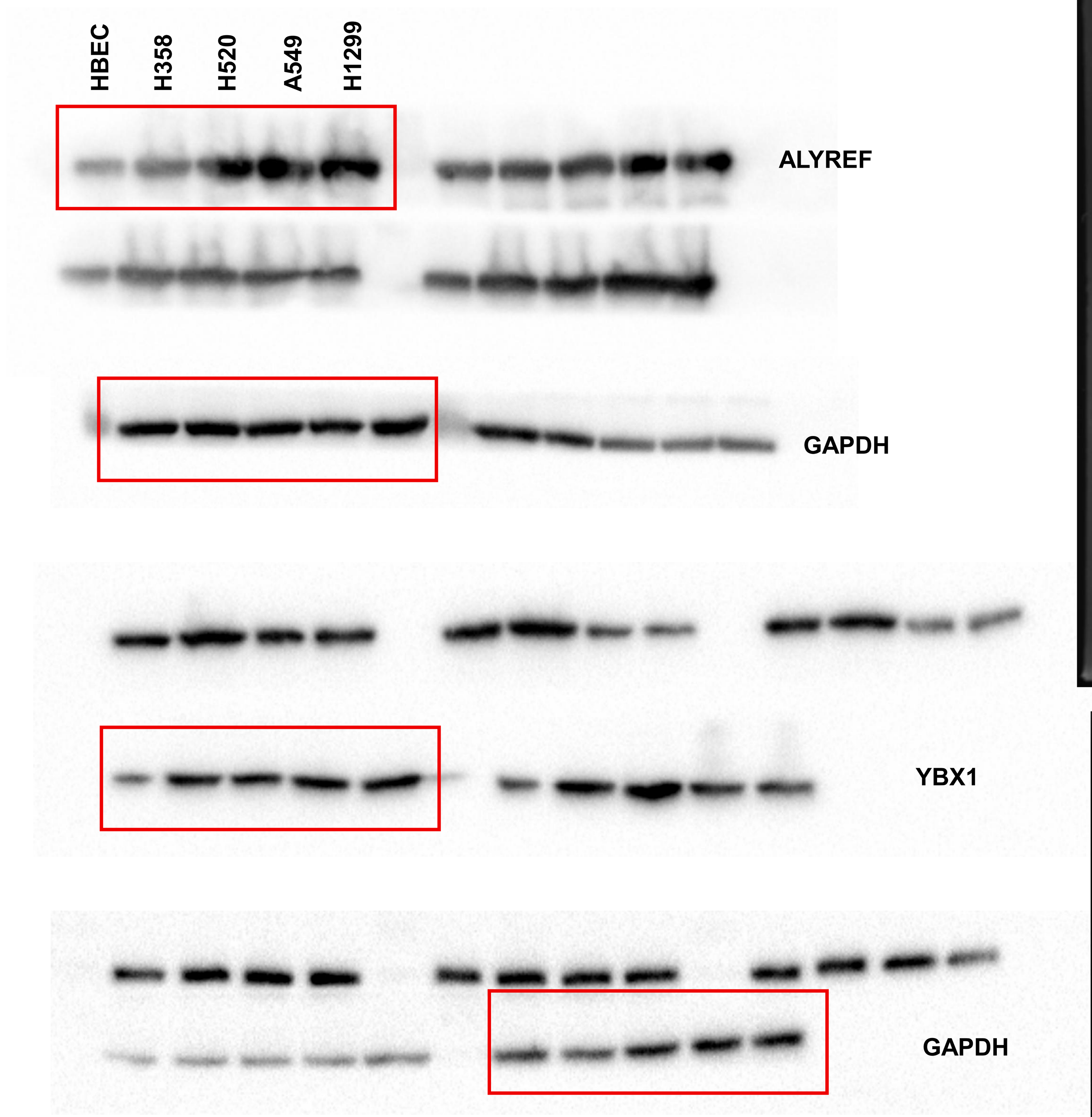

Fig S3b

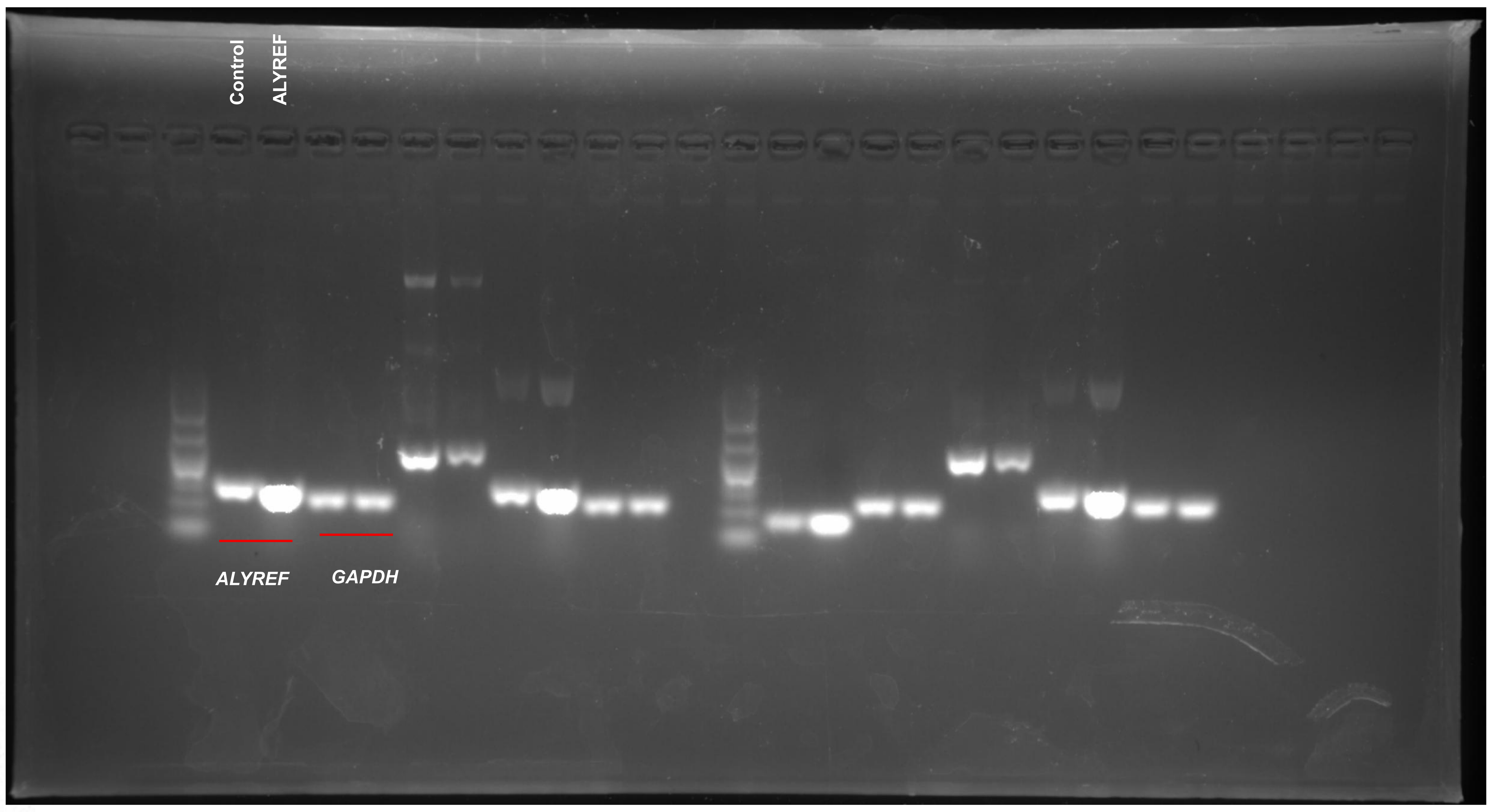

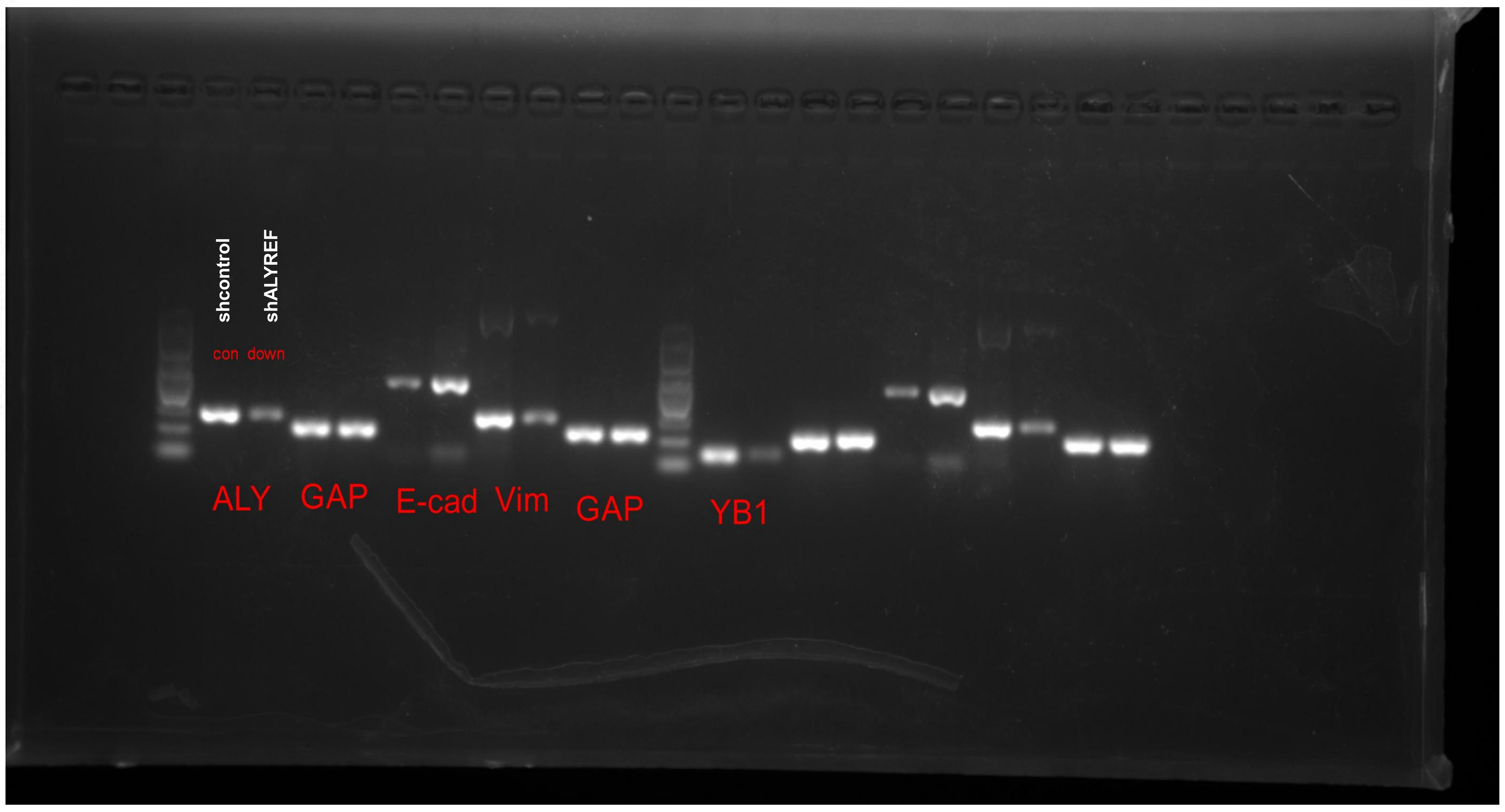

Fig S3b

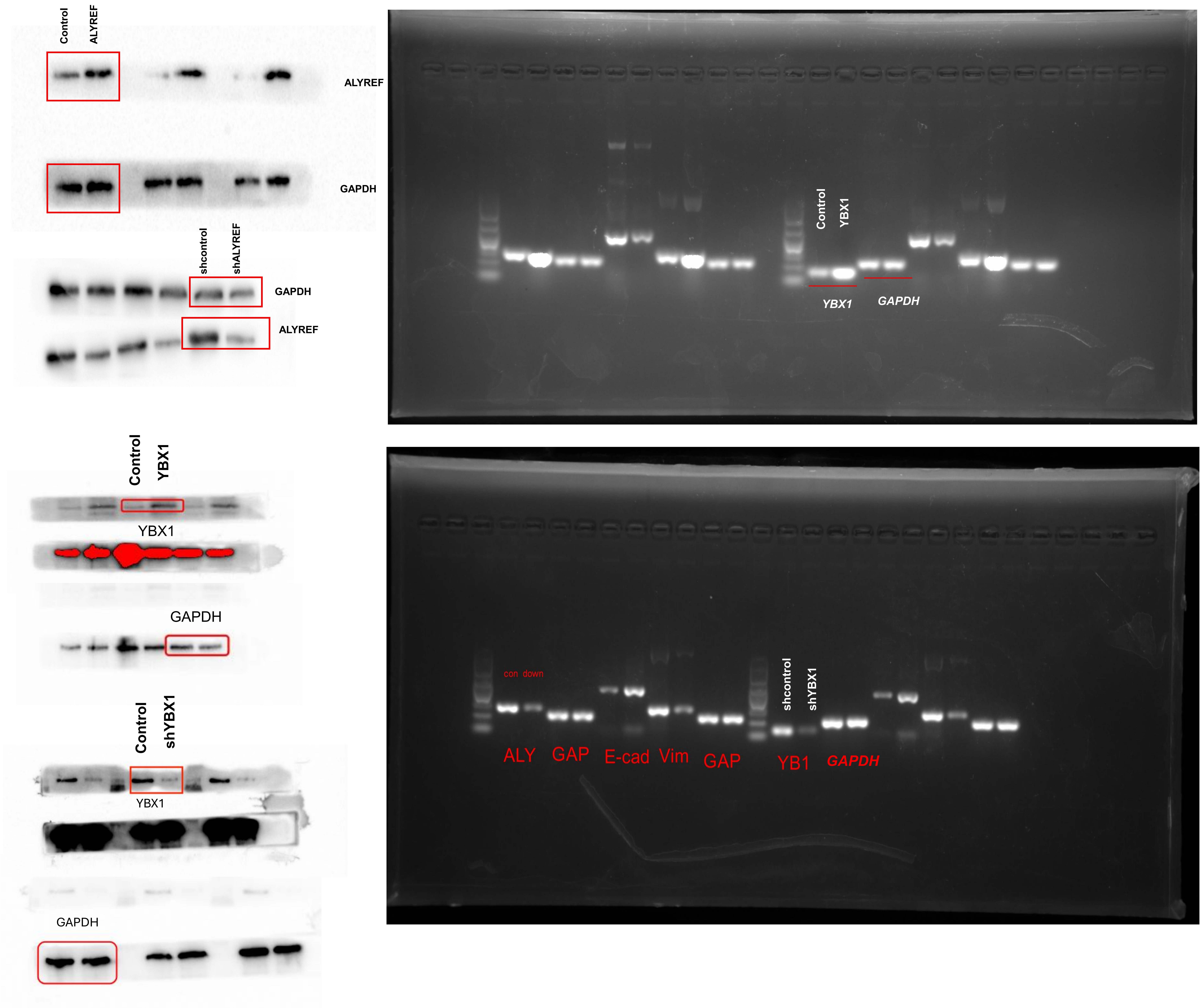

Fig S3f

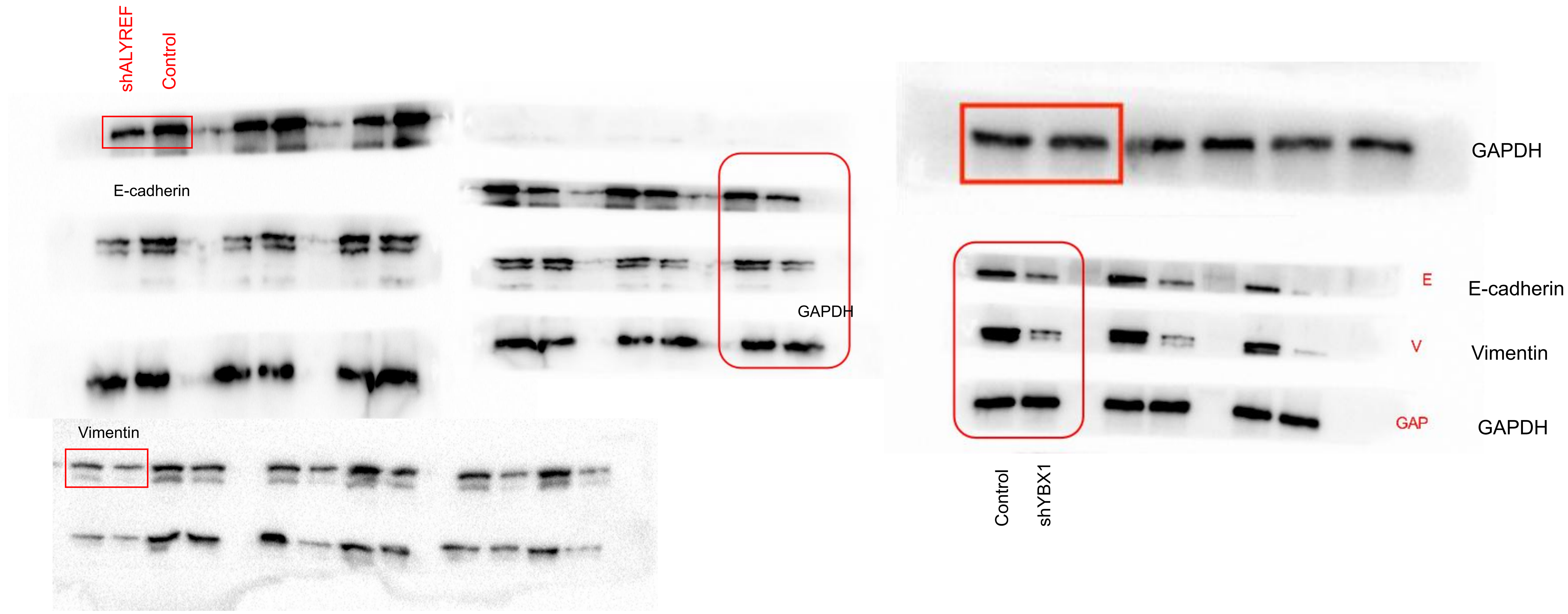

Fig S3h

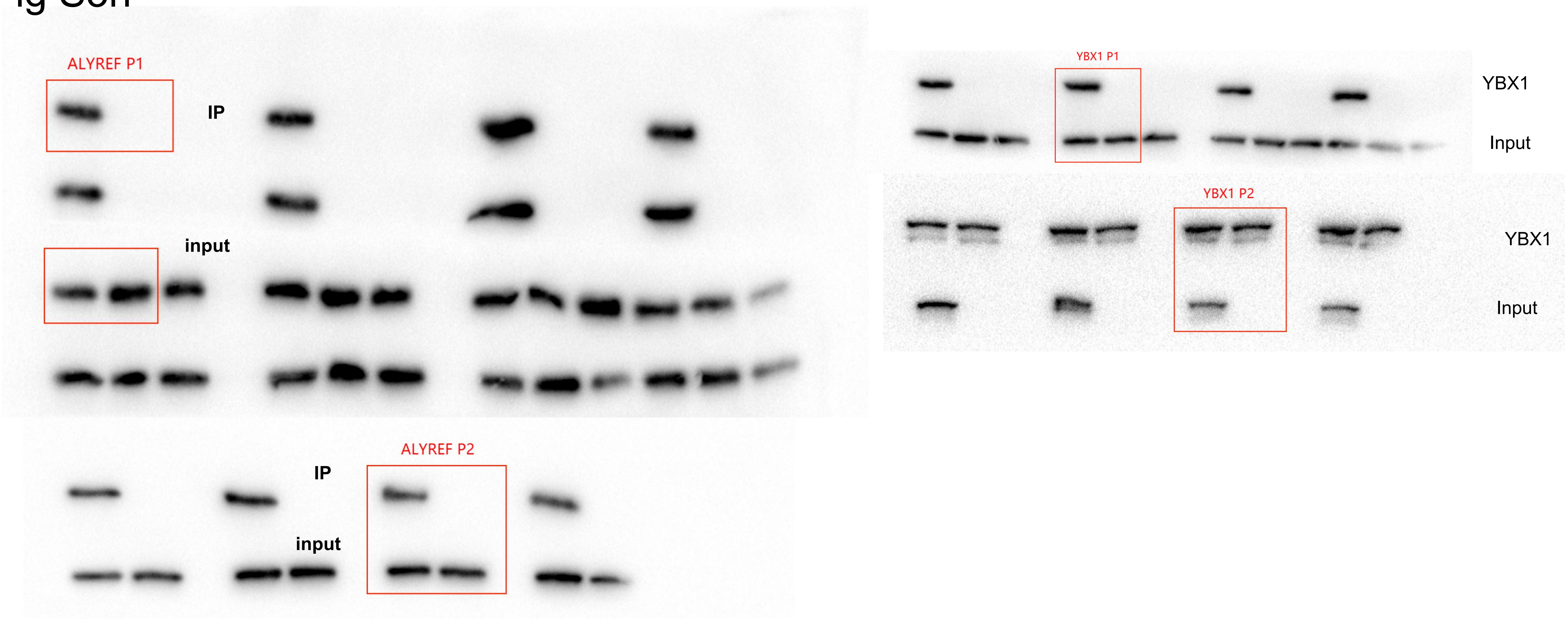

Fig S3n

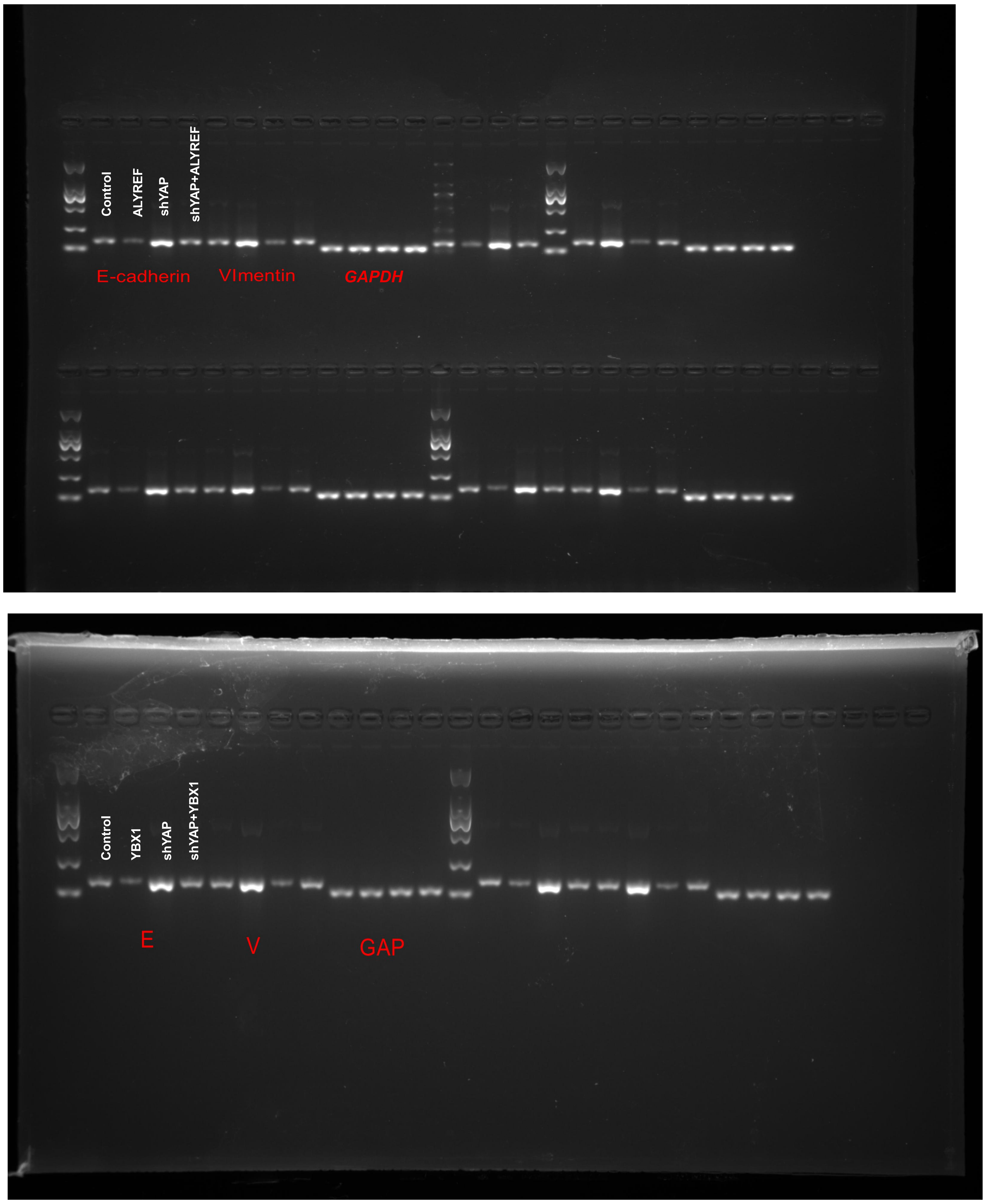

Fig S4e

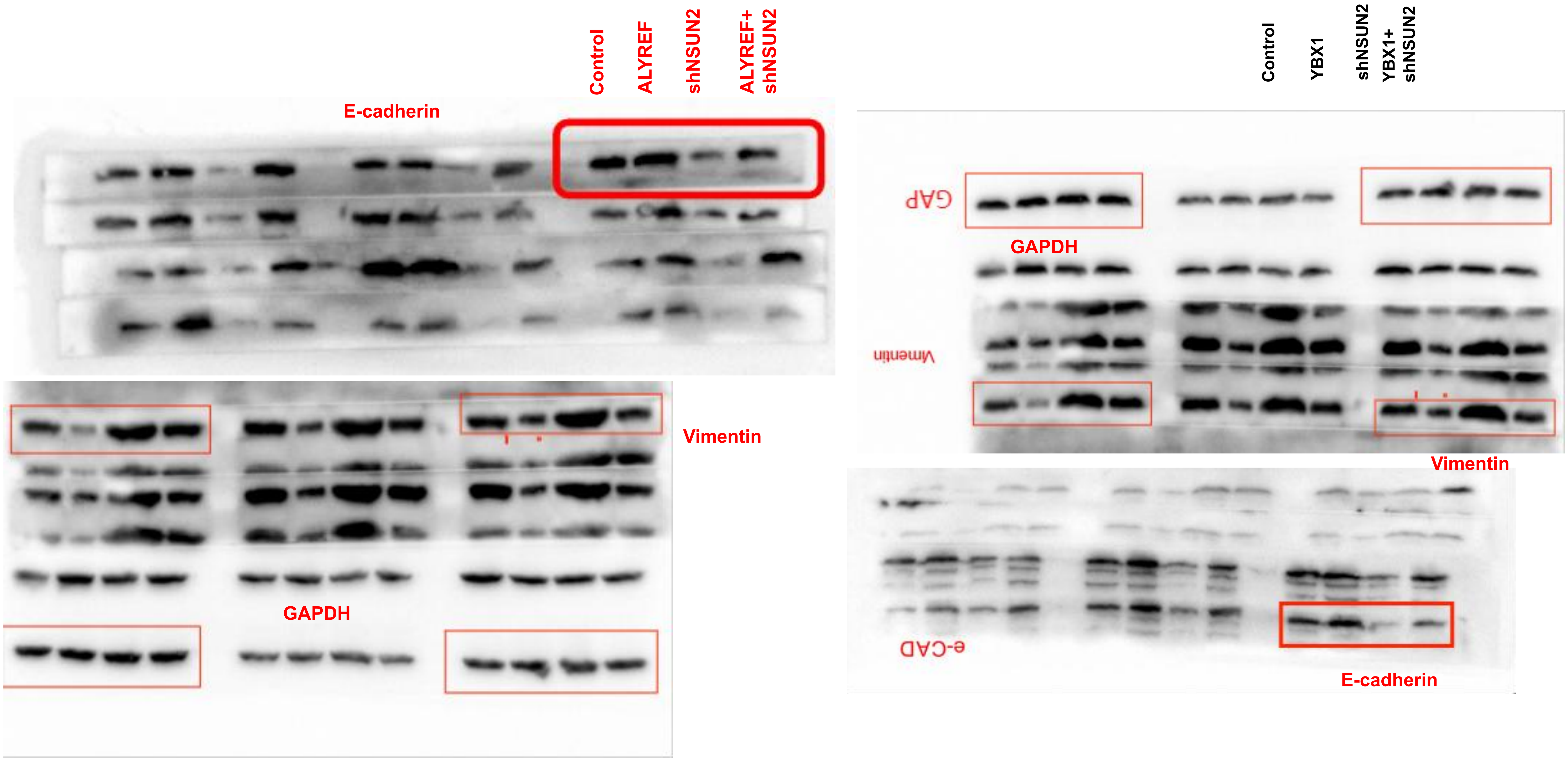

Fig S7b

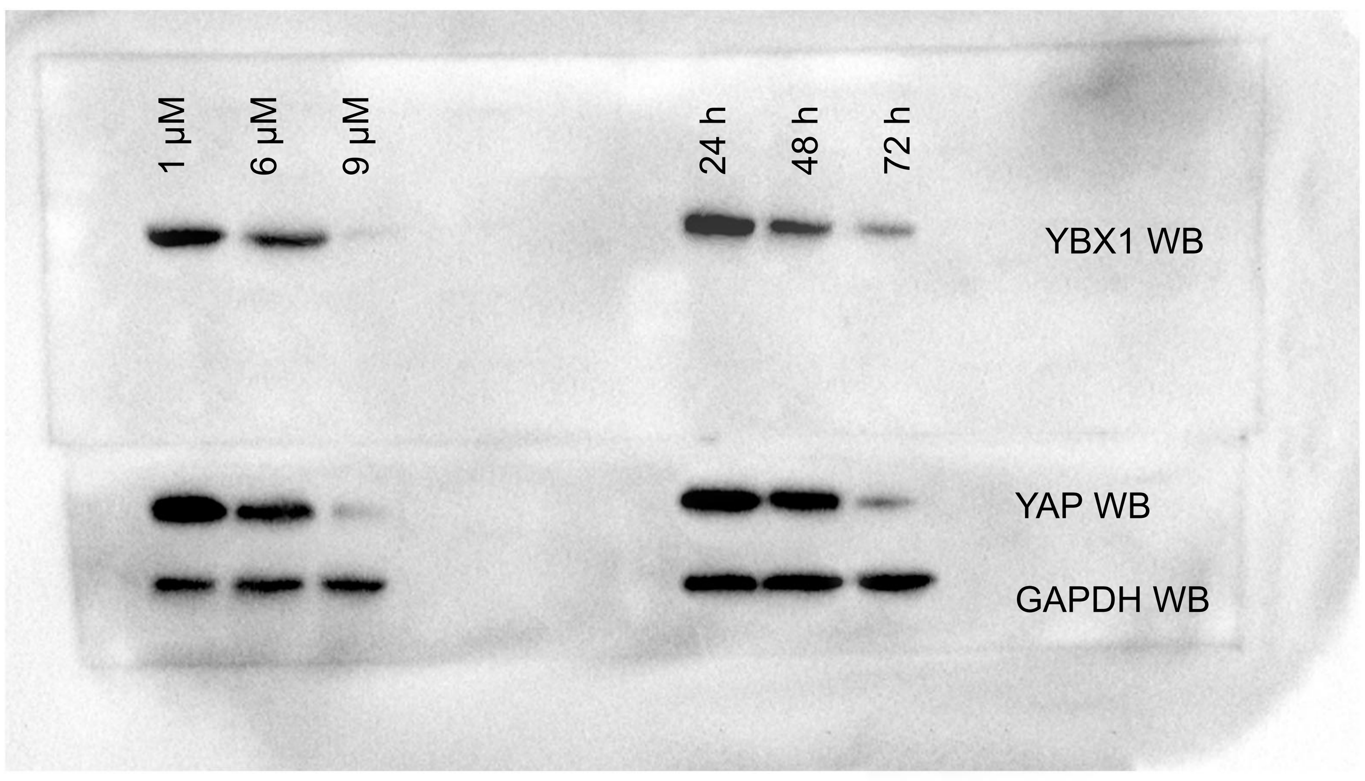

Supplement: Supplementary file 2 — Unedited blot and gel images [file 41419_2025_8353_MOESM2_ESM.pdf]
